# Artificial Intelligence and Innovation in Oral Health Care Sciences: A Conceptual Review

**DOI:** 10.3390/healthcare13243327

**Published:** 2025-12-18

**Authors:** Marco Dettori, Demetrio Lamloum, Peter Lingström, Guglielmo Campus

**Affiliations:** 1 Department of Medicine, Surgery and Pharmacy, University of Sassari, 07100 Sassari, Italy; 2 Department of Restorative, Paediatric and Preventive Dentistry, University of Bern, 3012 Bern, Switzerland; demetrio.lamloum@students.unibe.ch; 3 Department of Public Health, Experimental and Forensic Medicine, University of Pavia, 27100 Pavia, Italy; 4 Graduate School for Health Sciences, University of Bern, 3012 Bern, Switzerland; 5 Heidelberg Institute of Global Health, Section for Oral Health, Heidelberg University, 69117 Heidelberg, Germany; 6 Department of Cariology, Institute of Odontology, Sahlgrenska Academy, University of Gothenburg, 405 30 Gothenburg, Sweden; peter.lingstrom@odontologi.gu.se; 7 Department of Oral and Maxillofacial Sciences, Sapienza University of Rome, 00185 Rome, Italy

**Keywords:** artificial intelligence, oral health, dentistry, dental caries, machine learning, public health, equity, predictive models

## Abstract

**Highlights:**

**What are the main findings?**

- A bibliometric and conceptual review (2020–2025) identified dominant clusters in AI and dentistry: diagnostic imaging, decision-support systems, teledentistry/education, and ethics–governance.
- Research trends show a growing focus on generative and multimodal AI, with increasing attention to transparency, explainability, and fairness in dental applications.

**What are the implications of the main findings?**

- AI is redefining oral healthcare by improving diagnostic accuracy and personalization, yet responsible translation demands validated, interpretable, and equitable systems.
- Collaboration between clinicians, data scientists, and policymakers is essential to ensure ethical integration of AI into dental practice and education.

**Abstract:**

**Background/Objectives**: Artificial intelligence (AI) has rapidly evolved from experimental algorithms to transformative tools in clinical dentistry. Between 2020 and 2025, advances in machine learning (ML) and deep learning (DL) have reshaped diagnostic imaging, caries detection, prosthodontic design, and teledentistry, while raising new ethical and regulatory challenges. This study aimed to provide a comprehensive bibliometric and conceptual review of AI applications in dental care, highlighting research trends, thematic clusters, and future directions for equitable and responsible integration of AI technologies. In addition, the review further considers the implications of AI adoption for patient-centered care, including its potential role in supporting shared decision-making processes in oral healthcare. **Methods**: A comprehensive search was conducted in PubMed, Scopus and Embase for articles published between January 2020 and October 2025 using AI-related keywords in dentistry. Eligible records were analyzed using VOSviewer (v.1.6.20) to map co-occurrence networks of keywords, authors, and citations. A narrative synthesis complemented the bibliometric mapping, emphasizing conceptual and ethical dimensions of AI adoption in oral health care. **Results**: A total of 50 documents met the inclusion criteria. Bibliometric network visualization identified that the largest and most interconnected clusters were centered around the keywords “artificial intelligence,” “machine learning,” and “deep learning,” reflecting the technological backbone of AI-based applications in dentistry. Thematic evolution analysis indicated increasing interest in generative and multimodal AI models, explainability, and fairness in clinical deployment. **Conclusions**: AI has become a core driver of innovation in dentistry, enabling precision diagnostics and personalized care. However, responsible translation requires robust validation, transparency, and ethical oversight. Future research should integrate interdisciplinary approaches linking AI performance, patient outcomes, and equity in oral health.

## 1. Introduction

Artificial intelligence (AI) has evolved from a theoretical construct into one of the most disruptive technologies in the health sciences. In dentistry, AI has undergone a significant transformation, redefining clinical workflows, diagnostic paradigms, and patient engagement [1]. This transformation has been facilitated by the utilization of machine learning (ML), deep learning (DL), and data analytics, which have enabled the processing of complex multimodal data encountered by oral health professionals in their daily practice [2,3]. The period between 2020 and 2025 saw a remarkable surge in the adoption of AI within the field of dentistry [4]. This adoption encompassed a wide range of applications within different dental areas, including diagnostic radiography, caries detection, prosthodontic design, orthodontic simulation, teledentistry, and educational reform [4,5,6,7]. In recent years, the use of AI in dentistry has widened considerably. Several fields now employ these tools, ranging from cephalometric landmark identification to the analysis of radiographic images, predictive approaches for caries assessment, and various elements of the digital workflow [8,9].

The digital transformation of oral health care commenced decades earlier with the introduction of Electronic Health Records (EHRs), digital radiography, and computer-aided design/computer-aided manufacturing (CAD/CAM) systems [10]. However, the capacity of AI to directly “learn” patterns from data distinguishes it fundamentally from earlier technologies. AI models have been shown to identify relationships between clinical, radiographic, and behavioural variables that exceed human cognitive limits [11,12,13]. In diagnostic imaging [14,15], convolutional neural networks (CNNs) have achieved expert-level accuracy in detecting caries, periapical lesions, and periodontal bone loss, while transformer architectures have begun to enhance image–text interpretation by integrating radiographic features with clinical descriptions [16]. It is equally important to note the capacity of AI to promote equitable innovation [17,18,19]. The global disparities in oral health that have been observed are a matter of concern, with the impact being most acutely felt by those in the lowest income brackets and by members of vulnerable populations [20].

The growing presence of AI in dental practice requires a careful and balanced approach. Its adoption raises several questions that go beyond technical performance, touching on issues such as potential biases in algorithms, the protection of sensitive data, the clarity of decision-making processes, and the responsibilities assumed by clinicians [18]. Regulatory bodies such as the European Union (EU) [21] and the U.S. Food and Drug Administration (FDA) classify most diagnostic AI systems as high-risk devices, requiring rigorous validation and post-market monitoring [19]. The forthcoming decade will determine whether AI becomes a sustainable and equitable adjunct to oral health care or remains a fragmented collection of unregulated technologies [22].

Alongside the rapid spread of digital tools, the integration of AI in dentistry also needs to remain consistent with contemporary approaches to patient-centered care. These approaches no longer view clinical decisions as the product of the clinician’s judgement alone; instead, choices are increasingly shaped through a shared and collaborative process [23]. Within this framework, shared decision-making (SDM) has become a key component of patient-centered oral health care, as it promotes partnership, openness, and the combination of clinical evidence with patients’ own priorities and expectations [23,24]. Rather than relying on a purely provider-driven pathway, SDM encourages a more participatory and inclusive way of planning care [25]. As AI systems begin to influence diagnostic interpretation, risk assessment, prognostic reasoning, and treatment planning, clarifying how these technologies may contribute to SDM becomes an important question [26].

Early observations suggest that AI-supported applications could aid SDM by making information more accessible, tailoring explanations to individual patients, and enhancing access through tools such as teledentistry or mobile platforms [27,28,29]. At the same time, limits in transparency, cultural suitability, or in the equitable distribution of AI resources may weaken patient autonomy and widen existing disparities [30,31,32].

For these reasons, incorporating SDM principles into the assessment and use of AI systems is essential for promoting ethical, fair, and trustworthy innovation in dental care [33]. The purpose of this review is therefore to summarize the literature published between 2020 and 2025 and to examine how current developments in dental AI relate to clinical usefulness, ethical issues, equity considerations, and shared decision-making. More specifically, the review explores how AI may support—or, in some circumstances, challenge—patient-centered oral health care and outlines the governance issues that are likely to influence future progress in this field.

## 2. Materials and Methods

### 2.1. Search Strategy and Inclusion/Exclusion Criteria

Two authors performed an electronic literature search from 1 January 2020 to 1 October 2025, using the keywords “Artificial Intelligence” and “Dental Caries”. Dental caries was selected as the index condition for developing the search strategy because it is the area in which AI has been most widely studied within preventive dentistry and offers terminology that is consistent across the major databases. Choosing this anchor condition allowed us to design a search process that was both reproducible and sufficiently broad, without restricting the subsequent bibliometric or conceptual analyses, which considered the wider range of AI applications in oral health. Search strings were adapted for each database, namely PubMed, Scopus and Embase. The search strings are displayed in Table 1.

Manual screening was carried out using a set of predefined inclusion criteria: (i) publications dated between 2020 and 2025; (ii) studies examining the use of artificial intelligence in any aspect of oral health, including diagnostic, predictive, preventive, public-health, educational, or ethical perspectives; (iii) peer-reviewed original research articles, reviews, or conceptual papers; and (iv) works available in English. Items were excluded if they consisted of editorials, letters, conference proceedings, non-dental applications or if the study did not contain a clearly identifiable AI component.

### 2.2. Bibliometrics Network Analysis

A bibliometric network analysis was conducted to identify the main research topics and the conceptual structure of the scientific literature on AI in dental care. The analysis was based on the dataset obtained from the literature selection process described in the previous section.

After manually selecting titles and abstracts to ensure their relevance to the topic, the articles were included. Bibliometric network analysis was performed using VOSviewer (Centre for Science and Technology Studies, Leiden University, The Netherlands; version 1.6.20), a software tool for constructing and visualising bibliometric networks (available online: https://www.vosviewer.com accessed on 18 October 2025) [34]. The metadata of the selected publications, including titles, abstracts and authors’ keywords, were imported into the software (Table S1). A co-occurrence analysis of author keywords was performed to examine the relationships between terms and highlight the most representative research areas within the field. The minimum occurrence threshold was set to one, allowing for the inclusion of all keywords that appeared at least once in the dataset. The graphical abstract is provided. GenAi was used to generate graphics.

## 3. Results

### 3.1. Flowchart

**Figure 1:**
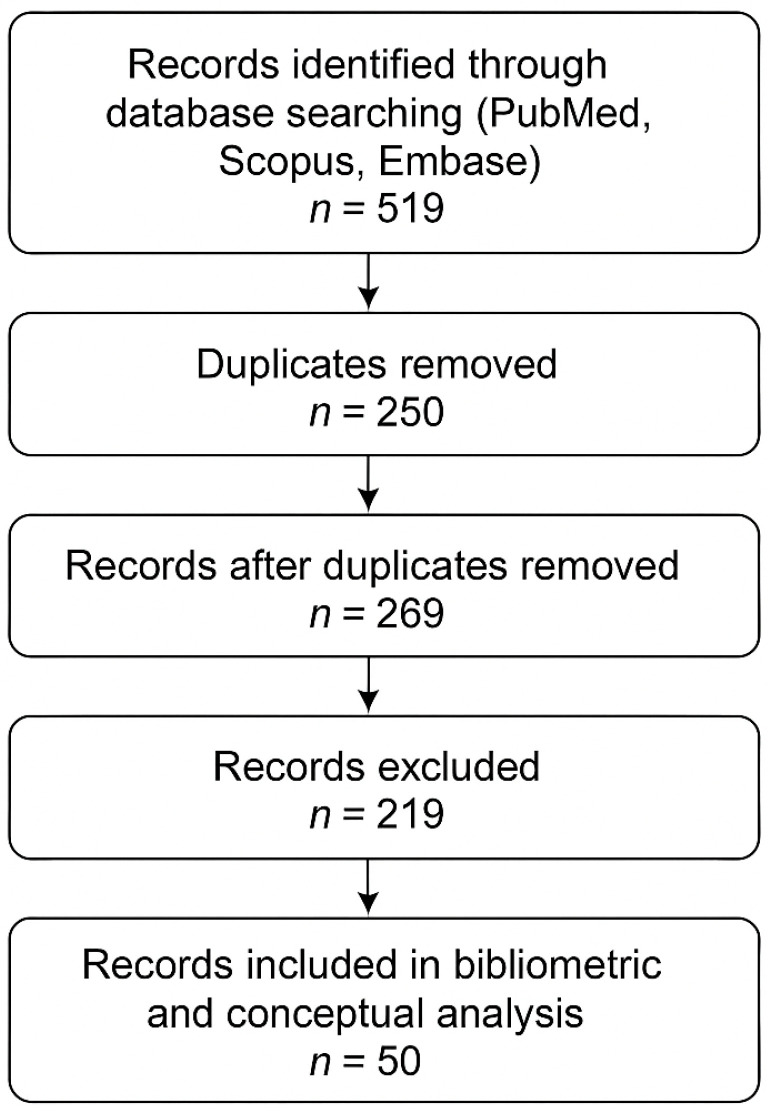
PRISMA-like flow chart of the search procedures.

The search process is displayed in Figure 1. Overall, 519 records were retrieved from PubMed, Scopus, and Embase. After the removal of 250 duplicates, 269 unique records remained. Applying the predefined inclusion and exclusion criteria to titles and abstracts yielded 50 records that were retained for bibliometric mapping and conceptual synthesis, while 219 records were excluded.

### 3.2. Bibliometric Network Analysis

**Figure 2:**
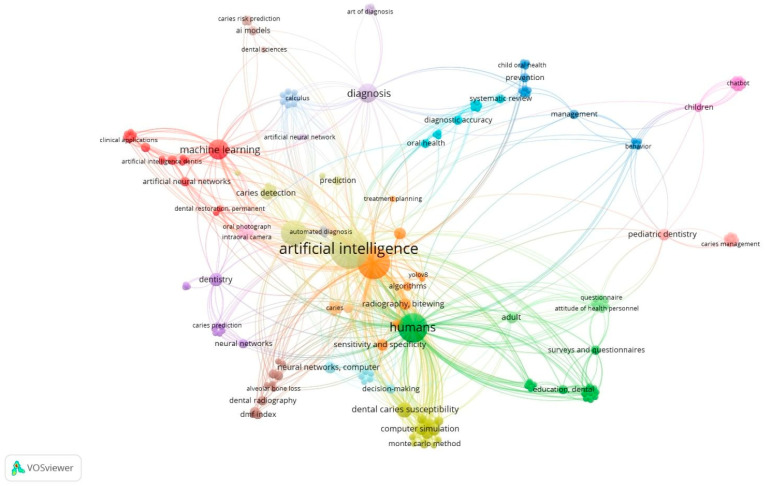
Network visualization map of author keyword co-occurrence generated using VOSviewer (version 1.6.20). Node size represents keyword frequency; link strength indicates co-occurrence relationships.

The bibliometric analysis included a total of 50 articles addressing the use of AI in dental care. The keyword co-occurrence map, generated using VOSviewer (version 1.6.20), revealed the main conceptual relationships and thematic clusters within the selected literature (Figure 2).

In total, 193 unique author keywords were identified. Since the minimum occurrence threshold was set to one, all keywords present in the dataset were included in the analysis. The visualization map showed several clusters that represent different research focuses within the field. The bibliometric mapping yielded four thematic clusters, ranging in size from approximately 12 to 21 publications. The largest cluster was centred on diagnostic imaging and radiograph interpretation. Other clusters reflected applications in caries detection and prediction, pediatric prevention and management, and AI-supported decision-making and clinical planning. These domains represent the most frequent themes in AI-related dental research during the study period.

The largest and most interconnected clusters were centred around the keywords “artificial intelligence”, “machine learning”, and “deep learning”, reflecting the technological backbone of AI-based applications in dentistry. Other clusters were mainly related to “diagnosis,” “cone beam computed tomography (CBCT)”, and “image analysis,” indicating a strong focus on diagnostic imaging and automated detection systems. The other clusters highlighted terms such as “dentistry”, “dental caries”, “caries prediction”, and “caries prevention”, representing the application of AI tools to specific clinical domains. Finally, a small (but noteworthy) cluster included the keyword “decision making,” which appeared to be associated with terms related to diagnostic support and predictive modelling.

## 4. Discussion

The findings obtained from the literature review and the bibliometric network analysis provided a comprehensive overview of current research on AI applications in dental care. The combination of these approaches allowed not only the identification of the most frequently investigated topics but also the recognition of conceptual relationships and emerging trends within the field. The science mapping highlighted several thematic clusters, which reflect distinct yet interconnected research directions, ranging from diagnostic imaging and automated detection systems to clinical decision support and orthodontic applications.

The bibliometric network analysis revealed that “artificial intelligence”, “machine learning”, and “deep learning” form the most interconnected cluster in dental research, underscoring the technological foundations underpinning current innovations. Within this domain, one of the most transformative applications is the integration of AI into teledentistry and digital public health systems.

AI-enabled teledentistry [4,5] represents a paradigm shift from clinic-based to distributed models of oral health care delivery [35]. CNNs embedded in smartphone applications are capable of detecting carious lesions, gingival inflammation, and mucosal abnormalities directly from intraoral photographs, automating the triage process [4,14,15,16,36]. Findings from Azimi et al. [7] show that AI can be used effectively for remote screening activities, offering a practical way to expand access to oral health services. Other umbrella reviews support this perspective, noting that AI systems tend to perform well in identifying caries and various types of oral lesions [14]. The integration of de-identified imaging data into centralized databases enables population-level surveillance, facilitating the identification of caries hotspots and informing public-health interventions such as fluoridation or mobile-clinic deployment. Integrated with mHealth tools, smart toothbrushes, sensors, and reinforcement-learning reminders, these systems enhance prevention [21]. Cloud–edge architectures process images locally for privacy and sync with EHRs via HL7 FHIR, supporting more efficient data management in low-resource settings [37]. Equity depends on bridging digital divides through infrastructure and culturally adapted interfaces.

Beyond improvements in diagnostic accuracy, the integration of AI-based tools in dentistry has important implications for shared decision-making. Appropriately designed AI systems may enhance clinicians’ ability to communicate risk in a transparent and comprehensible manner, support the presentation of individualized treatment alternatives, and assist in identifying patient preferences across preventive and therapeutic options. However, these potential benefits are contingent upon the interpretability and accessibility of AI technologies. Limited transparency in algorithmic decision-making, as well as disparities in access to digital tools, may compromise patient autonomy and erode trust in clinical recommendations. Accordingly, the development and implementation of AI in dental practice should be explicitly aligned with established principles of shared decision-making. Once a branch of telemedicine, teledentistry has become an AI-driven digital ecosystem. Accelerated by COVID-19, it now ensures care continuity when clinics are inaccessible. Between 2020–2025, AI redefined the field by boosting diagnostic accuracy, automating triage, optimizing scheduling, and enabling real-time engagement. Modern systems combine image-based diagnosis, virtual consultation, cloud data exchange, and AI decision support. CNNs analyse intraoral photos to detect lesions, while NLP algorithms triage by urgency, transforming teledentistry from passive communication into an active diagnostic tool. AI’s most validated function is diagnostic triage. Recent work has also explored the use of spectral and multispectral imaging together with deep learning approaches, demonstrating that these techniques can automatically identify caries and calculus and may offer a low-cost option where radiographic equipment is unavailable [38]. At the same time, new in-vivo multispectral datasets that include segmentation have been released, providing researchers with resources that can support both the training and evaluation of these models [39]. Integration with EHRs maintains seamless continuity of care [31]. AI also enables public-health surveillance. Aggregated image data reveal epidemiologic trends and guide interventions [37,40]. AI assists in workforce planning, predicting treatment demand and optimizing appointments [10], while wearable data tracks oral-health behaviours such as brushing frequency and sugar intake [29]. The convergence of AI, teledentistry, and mHealth underpins personalized prevention. Smartphones, intraoral scanners, and smart brushes provide continuous behavioural and imaging data; vision algorithms quantify plaque, accelerometers assess brushing, and AI models deliver tailored feedback [29]. Affordable smartphones make such systems scalable, though hybrid offline–cloud models remain essential where connectivity is limited [38].

Equity remains the central challenge in the digital transformation of oral health. Unequal access to infrastructure, training, and technology risks amplifying existing disparities. The WHO Global Oral Health Report (2025) and the FDI Vision 2030 Implementation Framework (2024) identify digital inclusion as a prerequisite for equitable innovation, emphasizing that AI developed in high-income contexts must be adapted to reflect local epidemiology, culture, and clinical realities [20,41].

Embedding AI within socially and culturally congruent care systems, supported by capacity building and ethical education, ensures that innovation reduces, rather than reinforces, inequality [17,18]. Dental curricula should therefore integrate digital literacy, data ethics, and algorithmic reasoning [18]. Upholding transparency, accountability, privacy, inclusivity, and sustainability will enable AI to serve as an ethical catalyst for global oral health equity [18,31].

Despite strong performance metrics, preventive-AI adoption faces socio-technical hurdles. Data heterogeneity across regions and devices can degrade algorithm accuracy. Overreliance on digital infrastructure risks widening disparities if low-income populations lack connectivity or compatible devices [38]. To counter this, hybrid models combining AI-driven remote screening with community health-worker engagement have proven effective [41]. Ethical transparency, explicitly communicating how risk scores are generated and used, is essential to maintain patient autonomy and trust [25,32].

The extent to which clinicians rely on AI tools still depends heavily on how trustworthy their outputs appear. Factors such as the origin of the datasets used for training, the quality of the annotations, and the degree of transparency in the models all play a central role in shaping that trust. As AI shifts dentistry toward prediction and prevention rather than repair, its success will depend on integration into public-health frameworks and reimbursement systems that value preventive outcomes [10,18].

Expansion depends on secure, interoperable infrastructure. Cloud–edge architectures process data locally and on GPU servers, using TLS 1.3 encryption and metadata stripping. HL7 FHIR standards integrate AI outputs with national EHRs, enabling longitudinal tracking and large-scale research [42]. However, cross-border data transfer raises privacy issues under GDPR and HIPAA, requiring robust ethical governance to balance global scalability with individual rights.

In essence, robust AI adoption in dentistry requires a dual commitment to technical transparency and ethical accountability, ensuring that innovation advances clinical quality without compromising equity or trust [43]. The reliability of AI-driven oral health systems depends on robust data stewardship. Privacy-by-design principles, ethical auditing, and equitable access to secure technologies are essential to ensure that digital innovation strengthens rather than undermines trust, accountability, and inclusivity in oral healthcare.

## 5. Study Limitations, Future Perspectives

This study presents some limitations that should be acknowledged. First, the bibliometric analysis was based on a relatively small dataset of 50 publications, which may not capture the full breadth of the scientific output related to artificial intelligence in dental care. Although the selection process was carefully performed, the inclusion of articles based primarily on title relevance may have introduced a selection bias. Second, the analysis relied exclusively on author-provided keywords, which may vary in accuracy and consistency among different studies. The use of a minimum occurrence threshold of one allowed a comprehensive inclusion of terms, but it may have also increased noise in the network visualization. Additionally, only one bibliometric tool was used, and the analysis did not include citation or co-authorship mapping, which could have provided complementary insights. Finally, the dynamic and rapidly evolving nature of AI research means that newer publications may have emerged after the data collection period, potentially affecting the representativeness of the findings.

Automated text-analysis techniques (such as topic modelling, unsupervised clustering, or NLP-based extraction) were not applied in this study because they did not align with the methodological objectives of the review. The publications included showed marked variation in study design, analytical detail, reporting formats, and the types of AI tools assessed, making it difficult to assemble a coherent textual dataset suitable for computational analysis. Under such circumstances, text-mining procedures would likely have produced unstable clusters and outputs that were difficult to interpret, ultimately reducing rather than improving the robustness of the synthesis. For this reason, and consistent with the conceptual aims of the work, we opted for bibliometric mapping combined with expert-guided qualitative interpretation instead of relying on automated text processing.

Although dental caries terminology served as an operational starting point for structuring the search, this did not narrow the conceptual boundaries of the review. The studies ultimately included covered a wide range of AI applications in oral healthcare, and the resulting cluster configuration confirmed that caries-related themes represented only one of several domains, rather than the dominant focus of the literature examined. Despite these limitations, the integration of bibliometric mapping with a structured literature review offers a robust and transparent overview of current research trends and conceptual developments in the application of AI to dental care. This combined approach allowed us to capture both structural patterns within the literature and the conceptual nuances emerging from qualitative interpretation.

The future of dental AI is predicted to be characterised by a shift towards multimodal integration, which involves the combination of imaging, clinical, and behavioural data through transformer architectures. The utilisation of federated learning networks has the potential to establish interconnected systems among global institutions while ensuring the confidentiality of data. Human-in-the-loop systems will facilitate adaptive reinforcement learning guided by clinician feedback. However, the process of attaining maturity necessitates constant vigilance. The profession is currently at an inflexion point: the capacity of AI to either perpetuate or dismantle existing inequalities is contingent on its integration within a framework of equitable innovation.

## 6. Conclusions

This review examined developments in dental artificial intelligence published between 2020 and 2025, combining bibliometric mapping with a perspective informed by shared decision-making. Taken together, the studies included show that diagnostic, predictive, and preventive applications of AI are gradually moving toward models of care that place greater emphasis on patient needs and participation. Ethical issues and questions of equity increasingly influence this evolution, pointing to the importance of deploying AI in ways that remain transparent, accountable, and sensitive to social context.

While caries-related research continues to provide the most consolidated body of evidence, the scope of AI in oral health now extends to imaging, decision-support tools, mobile health applications, and broader public-health uses. The pace and quality of future progress will depend on strengthening interoperability across systems, developing clear governance arrangements, and ensuring adequate training so that AI enhances, rather than replaces, the clinical relationship. When implemented within care environments that respect ethical norms and cultural differences, AI has the potential to contribute meaningfully to preventive, equitable, and participatory approaches to oral healthcare.

## Figures and Tables

**Table 1:** Databases and related search strings used in the literature review process.

Database	Search String
PubMED	“(artificial intelligence”[MeSH Terms] OR (“artificial”[All Fields] AND “intelligence”[All Fields]) OR “artificial intelligence”[All Fields]) AND (“dental caries”[MeSH Terms] OR (“dental”[All Fields] AND “caries”[All Fields]) OR “dental caries”[All Fields]) AND (“prevent”[All Fields] OR “preventability”[All Fields] OR “preventable”[All Fields] OR “preventative”[All Fields] OR “preventatively”[All Fields] OR “preventatives”[All Fields] OR “prevented”[All Fields] OR “preventing”[All Fields] OR “prevention and control”[MeSH Subheading] OR (“prevention”[All Fields] AND “control”[All Fields]) OR “prevention and control”[All Fields] OR “prevention”[All Fields] OR “prevention s”[All Fields] OR “preventions”[All Fields] OR “preventive”[All Fields] OR “preventively”[All Fields] OR “preventives”[All Fields] OR “prevents”[All Fields])”
Scopus	(TITLE-ABS-KEY (dental caries) AND TITLE-ABS-KEY (ai) AND TITLE-ABS-KEY (oral health))
Embase	((‘artificial and intelligence’) AND (‘dental and caries’) AND (‘oral and health’))

## Data Availability

No new data were created.

## Supplementary Materials

The following supporting information can be downloaded at: https://www.mdpi.com/article/10.3390/healthcare13243327/s1, Table S1: Metadata of the selected publications.

## Abbreviations

The following abbreviations are used in this manuscript:

AI	Artificial Intelligence
ML	Machine Learning
DL	Deep Learning
EHRs	Electronic Health Records
CAD/CAM	Computer-Aided Design/Computer-Aided Manufacturing
FDI	Federation Dentaire Internationelle
WHO	World Health Organization
FDA	Food and Drugs Administration
