# Supplementary material for: Artificial Intelligence and Innovation in Oral Health Care Sciences: A Conceptual Review
Source: Healthcare (Basel). 2025 Dec 18;13(24):3327. doi: 10.3390/healthcare13243327 (PMC12733269; doi:10.3390/healthcare13243327)
Supplement: Supplementary file 1 [file healthcare-13-03327-s001.zip › healthcare-3998025-supplementary.pdf]

Table S1. Metadata of the selected publications, including titles, abstracts and authors' keywords, were imported into the software

PMID- 41008414
OWN - NLM
STAT- PubMed-not-MEDLINE
DCOM- 20250927
LR - 20250930
IS - 2227-9032 (Print)
IS - 2227-9032 (Electronic)
IS - 2227-9032 (Linking)
VI - 13
IP - 18
DP - 2025 Sep 12
TI - Teledentistry Improves Access to Oral Care: A Cluster Randomised Controlled
      Trial.
LID - 10.3390/healthcare13182282 [doi]
LID - 2282
AB - OBJECTIVES: There is a paucity of research evaluating the use of telehealth
      applications for preventive oral care, such as remote dental screening and oral
      health promotion. This study aimed to assess the efficacy of teledentistry in
      improving the oral health of school-aged children. METHODS: In this cluster
      randomised controlled trial, a total of 175 children aged 4-15 years were
      enrolled from six schools across Western Australia. The schools were randomly
      assigned to either the teledentistry or the control group. The intervention
      consisted of dental screening and oral health promotion. Qualified oral health
      therapists (OHTs) performed in-person dental examinations on participants in the
      control group. Different OHTs conducted dental photography-based screenings for
      participants in the teledentistry group. Both groups received screening reports
      and educational leaflets. Nine months later, in-person examinations were
      conducted on all participants in both groups to assess their dental condition and
      adherence to the dental advice provided at baseline. The primary outcomes
      included decay experience (dft/DFT index) and the proportion of children
      converting from a 'caries-free' state to a 'caries-active' state at the

follow-up. RESULTS: A total of 164 children completed the follow-up (mean age,
7.7 ¬± 2.4 years). At baseline, the prevalence of dental caries in the control and
teledentistry groups was 47% and 46%, respectively. The incidence of dental
caries in the teledentistry (10%) and control (12%) groups at follow-up was not
significantly different (p = 0.7). CONCLUSION: The findings suggest that
teledentistry has comparable efficacy to traditional preventive oral care in
maintaining oral health. Teledentistry may offer a viable solution for expanding
access to preventive oral care, especially for disadvantaged communities.
FAU - Azimi, Somayyeh
AU - Azimi S
AUID- ORCID: 0000-0002-1124-3783
AD - School of Health and Clinical Sciences, The University of Western Australia,
Perth 6009, Australia.
FAU - Bennamoun, Basheer
AU - Bennamoun B
AUID- ORCID: 0009-0008-2240-9276
AD - School of Health and Clinical Sciences, The University of Western Australia,
Perth 6009, Australia.
FAU - Mehdizadeh, Maryam
AU - Mehdizadeh M
AUID- ORCID: 0000-0003-2164-828X
AD - The Australian e-Health Research Centre, CSIRO, Perth 6151, Australia.
FAU - Vignarajan, Janardhan
AU - Vignarajan J
AUID- ORCID: 0000-0003-4738-5414
AD - The Australian e-Health Research Centre, CSIRO, Perth 6151, Australia.
FAU - Xiao, Di
AU - Xiao D
AD - TeleMedC Pty Ltd., Darra 4076, Australia.
FAU - Huang, Boyen
AU - Huang B
AUID- ORCID: 0000-0002-1246-7447
AD - School of Dentistry, University of Minnesota, Minneapolis, MN 55455, USA.
FAU - Spallek, Heiko
AU - Spallek H
AUID- ORCID: 0000-0001-6865-4818
AD - Faculty of Medicine and Health, School of Dentistry, The University of Sydney,
Sydney 2050, Australia.
AD - Eyes of AI, Sydney 2000, Australia.
AD - Dentroid, Canberra 2601, Australia.

FAU - Irving, Michelle
AU - Irving M
AD - The Office of Health and Medical Research, NSW Health, Sydney 1590, Australia.
FAU - Kruger, Estie
AU - Kruger E
AUID- ORCID: 0000-0002-4883-6793
AD - School of Health and Clinical Sciences, The University of Western Australia,
      Perth 6009, Australia.
FAU - Tennant, Marc
AU - Tennant M
AD - School of Health and Clinical Sciences, The University of Western Australia,
      Perth 6009, Australia.
FAU - Estai, Mohamed
AU - Estai M
AUID- ORCID: 0000-0001-7109-0267
AD - School of Health and Clinical Sciences, The University of Western Australia,
      Perth 6009, Australia.
LA - eng
GR - 2018-065/Financial Markets Foundation for Children/
PT - Journal Article
DEP - 20250912
PL - Switzerland
TA - Healthcare (Basel)
JT - Healthcare (Basel, Switzerland)
JID - 101666525
PMC - PMC12469317
OTO - NOTNLM
OT - child oral health
OT - dental caries
OT - oral health promotion
OT - prevention
OT - screening
OT - telehealth
COIS- Di Xiao is affiliated with TeleMedC Pty Ltd. Heiko Spallek is the member of the
      Advisory Board of Eyes of AI and the member of the Scientific Advisory Committee
      for Dentroid. All authors declare no conflicts of interest.
EDAT- 2025/09/27 06:33
MHDA- 2025/09/27 06:34
PMCR- 2025/09/12
CRDT- 2025/09/27 01:08
PHST- 2025/08/04 00:00 [received]
PHST- 2025/09/05 00:00 [revised]
PHST- 2025/09/08 00:00 [accepted]
PHST- 2025/09/27 06:34 [medline]
PHST- 2025/09/27 06:33 [pubmed]
PHST- 2025/09/27 01:08 [entrez]
PHST- 2025/09/12 00:00 [pmc-release]
AID - healthcare13182282 [pii]

AID - healthcare-13-02282 [pii]
AID - 10.3390/healthcare13182282 [doi]
PST - epublish
SO - Healthcare (Basel). 2025 Sep 12;13(18):2282. doi:
10.3390/healthcare13182282.

PMID- 40863069
OWN - NLM
STAT- PubMed-not-MEDLINE
DCOM- 20250914
LR - 20250914
IS - 2304-6767 (Electronic)
IS - 2304-6767 (Linking)
VI - 13
IP - 8
DP - 2025 Aug 12
TI - Charting New Territory: AI Applications in Dental Caries Detection from Panoramic
      Imaging.
LID - 10.3390/dj13080366 [doi]
LID - 366
AB - Introduction: Dental caries remains a public health concern, and early detection
      prevents its progression and complications. Panoramic radiographs are essential
      diagnostic tools, yet the interpretation of panoramic X-rays varies among
      practitioners. Artificial intelligence (AI) presents a promising approach to
      enhance diagnostic accuracy in detecting dental caries. This scoping review
      examines the current literature on the use of AI programs to analyze panoramic
      radiographs for the diagnosis of dental caries. Methods: This scoping review
      searched PubMed, Scopus, Web of Science, and Dentistry and Oral Sciences Source,
      adhering to PRISMA guidelines. The review included peer-reviewed, original
      research published in English that investigated the use of AI to diagnose dental
      caries. Data were extracted on the AI model characteristics, advantages,
      disadvantages, and diagnostic performance. Results: Seven studies met the
      inclusion criteria. The Deep Learning Model achieved the highest performance
      (specificity 0.9487, accuracy 0.9789, F1 score 0.9245), followed by Diagnocat and
      Tooth Type Enhanced Transformer. Models such as CranioCatch and CariSeg showed
      moderate performance, while the Dental Caries Detection Network demonstrated the

lowest. Benefits included improved diagnostic support and workflow efficiency,
while limitations involved dataset biases, interpretability challenges, and
computational demands. Conclusions: Applying AI technologies to panoramic X-rays
demonstrates the potential for enhancing caries diagnosis, with some models
achieving near-expert performance. However, future research must address the
generalizability, transparency, and integration of AI models into clinical
practice. Future research should focus on diverse training datasets, explainable
AI development, clinical validation, and incorporating AI training into dental
education and training.
FAU - Hung, Man
AU - Hung M
AUID- ORCID: 0000-0003-2827-3740
AD - College of Dental Medicine, Roseman University of Health Sciences, South Jordan,
UT 84095, USA.
AD - Division of Public Health, University of Utah, Salt Lake City, UT 84108, USA.
AD - School of Business, University of Utah, Salt Lake City, UT 84112, USA.
AD - Primary Children's Hospital, Salt Lake City, UT 84113, USA.
FAU - Yevseyevich, Daniel
AU - Yevseyevich D
AD - College of Dental Medicine, Roseman University of Health Sciences, South Jordan,
UT 84095, USA.
FAU - Khazana, Milan
AU - Khazana M
AD - College of Dental Medicine, Roseman University of Health Sciences, South Jordan,
UT 84095, USA.
FAU - Schwartz, Connor
AU - Schwartz C
AUID- ORCID: 0009-0005-3954-582X
AD - College of Dental Medicine, Roseman University of Health Sciences, South Jordan,
UT 84095, USA.
AD - Library, Roseman University of Health Sciences, South Jordan, UT 84095, USA.
AD - Library, Noorda College of Osteopathic Medicine, Provo, UT 84606, USA.
FAU - Lipsky, Martin S
AU - Lipsky MS
AUID- ORCID: 0000-0003-1749-8477
AD - College of Dental Medicine, Roseman University of Health Sciences, South Jordan,
UT 84095, USA.

AD - Institute on Aging, Portland State University, Portland, OR 97207, USA.
LA - eng
PT - Journal Article
PT - Review
DEP - 20250812
PL - Switzerland
TA - Dent J (Basel)
JT - Dentistry journal
JID - 101716125
PMC - PMC12385533
OTO - NOTNLM
OT - AI programs
OT - dentistry
OT - healthcare
OT - panoramic radiograph
COIS- The authors declare no conflicts of interest.
EDAT- 2025/08/28 20:48
MHDA- 2025/08/28 20:49
PMCR- 2025/08/12
CRDT- 2025/08/27 10:05
PHST- 2025/06/19 00:00 [received]
PHST- 2025/08/07 00:00 [revised]
PHST- 2025/08/11 00:00 [accepted]
PHST- 2025/08/28 20:49 [medline]
PHST- 2025/08/28 20:48 [pubmed]
PHST- 2025/08/27 10:05 [entrez]
PHST- 2025/08/12 00:00 [pmc-release]
AID - dj13080366 [pii]
AID - dentistry-13-00366 [pii]
AID - 10.3390/dj13080366 [doi]
PST - epublish
SO - Dent J (Basel). 2025 Aug 12;13(8):366. doi: 10.3390/dj13080366.

PMID- 40802701
OWN - NLM
STAT- MEDLINE
DCOM- 20250826
LR - 20250829
IS - 1932-6203 (Electronic)
IS - 1932-6203 (Linking)
VI - 20
IP - 8
DP - 2025
TI - Examining the diagnostic accuracy of artificial intelligence for detecting dental
      caries across a range of imaging modalities: An umbrella review with
      meta-analysis.
PG - e0329986
LID - 10.1371/journal.pone.0329986 [doi]
LID - e0329986
AB - The objective of this systematic review was to systematically collect and analyze

multiple published systematic reviews to address the following research question
"Are artificial intelligence (AI) algorithms effective for the detection of
dental caries?". A systematic search of five electronic databases, including the
Cochrane Library, Embase, PubMed, Scopus, and Web of Science, was conducted until
October 15, 2024, with a language restriction to English. All fourteen systematic
reviews which assessed the performance of AI algorithms for the detection of
dental caries were included. From 137 primary original research studies within
the systematic reviews, only 20 reported the data necessary for inclusion in the
meta-analysis. Pooled sensitivity was 0.85 (95% Confidence Interval (CI): 0.83 to
0.93), specificity was 0.90 (95% CI: 0.85 to 0.95), and log diagnostic odds ratio
was 4.37 (95% CI: 3.16 to 6.27). Area under the summary ROC curve was 0.86.
Positive post-test probability was 79% and negative post-test probability was 6%.
In conclusion, this meta-analysis has revealed that caries diagnosis using AI is
accurate and its use in clinical practice is justified. Future studies should
focus on specific¬†subpopulations, depth of caries, and real-world performance
validation to further improve the accuracy of AI in caries diagnosis.
CI - Copyright: ¬© 2025 Arzani et al. This is an open access article distributed under
the terms of the Creative Commons Attribution License, which permits unrestricted
use, distribution, and reproduction in any medium, provided the original author
and source are credited.
FAU - Arzani, Sarah
AU - Arzani S
AUID- ORCID: 0000-0003-0014-471X
AD - Child Growth and Development Research Center, Research Institute for Primordial
Prevention of Non-Communicable Disease, Isfahan University of Medical Sciences,
Isfahan, Iran.
FAU - Karimi, Ali
AU - Karimi A
AD - Maxillogram Maxillofacial Surgery, Implantology and Biomaterial Research
Foundation, Istanbul, Turkey.
FAU - Iranmanesh, Pedram
AU - Iranmanesh P

AD - Department of Endodontics, Dental Research Center, Dental Research Institute,
      School of Dentistry, Isfahan University of Medical Sciences, Isfahan, Iran.
FAU - Yazdi, Maryam
AU - Yazdi M
AUID- ORCID: 0000-0003-3961-6702
AD - Child Growth and Development Research Center, Research Institute for Primordial
      Prevention of Non-Communicable Disease, Isfahan University of Medical Sciences,
      Isfahan, Iran.
AD - Diabetes Research Centre, College of Life Sciences, University of Leicester,
      Leicester, United Kingdom.
AD - Leicester Diabetes Centre, Leicester General Hospital, University Hospitals of
      Leicester NHS Trust, Leicester, United Kingdom.
FAU - Sabeti, Mohammad A
AU - Sabeti MA
AD - Advanced Specialty Program in Endodontics, UCSF School of Dentistry, University
      of California, San Francisco, California, United States of America.
FAU - Nekoofar, Mohammad Hossein
AU - Nekoofar MH
AD - Department of Endodontics, School of Dentistry, Tehran University of Medical
      Sciences, Tehran, Iran.
FAU - Kolahi, Jafar
AU - Kolahi J
AD - Independent Research Scientist, Founder of Dental Hypotheses, Isfahan, Iran.
FAU - Bang, Heejung
AU - Bang H
AD - Division of Biostatistics, Department of Public Health Sciences, School of
      Medicine, University of California, Davis, California, United States of America.
FAU - Dummer, Paul M H
AU - Dummer PMH
AD - Emeritus Professor of Restorative Dentistry, School of Dentistry, College of
      Biomedical and Life Sciences, Cardiff University, Cardiff, United Kingdom.
LA - eng
PT - Journal Article
PT - Meta-Analysis
PT - Systematic Review
DEP - 20250813
PL - United States
TA - PLoS One
JT - PloS one
JID - 101285081
SB - IM

MH - *Dental Caries/diagnosis/diagnostic imaging
MH - Humans
MH - *Artificial Intelligence
MH - Algorithms
MH - Sensitivity and Specificity
MH - ROC Curve
PMC - PMC12349118
COIS- The authors have declared that no competing interests exist.
EDAT- 2025/08/13 18:27
MHDA- 2025/08/31 20:07
PMCR- 2025/08/13
CRDT- 2025/08/13 13:53
PHST- 2025/02/09 00:00 [received]
PHST- 2025/07/24 00:00 [accepted]
PHST- 2025/08/31 20:07 [medline]
PHST- 2025/08/13 18:27 [pubmed]
PHST- 2025/08/13 13:53 [entrez]
PHST- 2025/08/13 00:00 [pmc-release]
AID - PONE-D-25-05467 [pii]
AID - 10.1371/journal.pone.0329986 [doi]
PST - epublish
SO - PLoS One. 2025 Aug 13;20(8):e0329986. doi:
10.1371/journal.pone.0329986.
      eCollection 2025.

PMID- 40786136
OWN - NLM
STAT- PubMed-not-MEDLINE
LR - 20250813
IS - 2212-4268 (Print)
IS - 2212-4276 (Electronic)
IS - 2212-4268 (Linking)
VI - 15
IP - 5
DP - 2025 Sep-Oct
TI - AI-driven approaches in the management of early childhood caries: A path toward
      global oral health.
PG - 1134-1140
LID - 10.1016/j.jobcr.2025.07.022 [doi]
AB - BACKGROUND: The integration of Artificial Intelligence (AI) with paediatric
      dentistry has unveiled transformative possibilities, particularly in mitigating
      the global burden of a prevalent yet preventable oral health issue, namely early
      childhood caries (ECC). ECC affects millions of children worldwide, leading to
      significant health, developmental, and economic challenges. This paper explores
      the application of AI-driven technologies, including machine learning and deep
      learning, in the detection, diagnosis, risk assessment, and management of ECC.

BRIEF SUMMARY: AI models leveraging dental radiographs and intraoral photographs
have demonstrated high accuracy in caries detection, while predictive algorithms
facilitate the identification of high-risk groups using patient demographics,
behavioural data, and even genetic markers. Smartphone applications equipped with
AI capabilities, such as AICaries, empower caregivers with tools for at-home
caries screening, enhancing accessibility and fostering preventive care.Today,
AI's role extends to optimizing healthcare utilization patterns and advancing
personalized treatment strategies, particularly in underserved regions where
traditional resources are scarce. Efforts to develop diverse training datasets
have not eliminated biases leading to concerns about fairness, discrimination and
privacy. Further, unregulated AI applications may worsen rather than reduce
health disparities. IMPLICATIONS FOR FUTURE RESEARCH: This review underscores the
potential of AI to revolutionize ECC prevention and management, paving the way
for equitable oral healthcare globally. It advocates for further
interdisciplinary research to refine AI tools, address practical challenges, and
support the development of evidence-based policies for widespread implementation.
Ultimately, AI emerges as a pivotal advancement in transitioning from disease
management to proactive oral health care strategies.
CI - ¬© 2025 The Authors.
FAU - Nayak, Prajna P
AU - Nayak PP
AD - Nitte (Deemed to Be University), AB Shetty Memorial Institute of Dental Sciences
(ABSMIDS), Department of Pediatric and Preventive Dentistry, Deralakatte,
Mangalore, 575018, Karnataka, India.
FAU - Shetty, Vabitha
AU - Shetty V
AD - Nitte (Deemed to Be University), AB Shetty Memorial Institute of Dental Sciences
(ABSMIDS), Department of Pediatric and Preventive Dentistry, Deralakatte,
Mangalore, 575018, Karnataka, India.
FAU - S, Shreya
AU - S S
AD - Nitte (Deemed to Be University), AB Shetty Memorial Institute of Dental Sciences

(ABSMIDS), Department of Pediatric and Preventive Dentistry, Deralakatte,
      Mangalore, 575018, Karnataka, India.
FAU - Zacharias, Liza
AU - Zacharias L
AD - Nitte (Deemed to Be University), AB Shetty Memorial Institute of Dental Sciences
      (ABSMIDS), Department of Pediatric and Preventive Dentistry, Deralakatte,
      Mangalore, 575018, Karnataka, India.
FAU - Gore, Isha
AU - Gore I
AD - Nitte (Deemed to Be University), AB Shetty Memorial Institute of Dental Sciences
      (ABSMIDS), Department of Pediatric and Preventive Dentistry, Deralakatte,
      Mangalore, 575018, Karnataka, India.
LA - eng
PT - Journal Article
PT - Review
DEP - 20250729
PL - Netherlands
TA - J Oral Biol Craniofac Res
JT - Journal of oral biology and craniofacial research
JID - 101619156
PMC - PMC12332940
OTO - NOTNLM
OT - AI models
OT - Artificial intelligence
OT - Caries diagnosis
OT - Caries risk prediction
OT - Early childhood caries
OT - Tele dentistry
COIS- The authors declare that they have no known competing financial interests or
      personal relationships that could have appeared to influence the work reported in
      this paper.
EDAT- 2025/08/11 17:20
MHDA- 2025/08/11 17:21
PMCR- 2025/07/29
CRDT- 2025/08/11 06:14
PHST- 2024/12/05 00:00 [received]
PHST- 2025/05/29 00:00 [revised]
PHST- 2025/07/24 00:00 [accepted]
PHST- 2025/08/11 17:21 [medline]
PHST- 2025/08/11 17:20 [pubmed]
PHST- 2025/08/11 06:14 [entrez]
PHST- 2025/07/29 00:00 [pmc-release]
AID - S2212-4268(25)00166-6 [pii]
AID - 10.1016/j.jobcr.2025.07.022 [doi]
PST - ppublish
SO - J Oral Biol Craniofac Res. 2025 Sep-Oct;15(5):1134-1140. doi:
      10.1016/j.jobcr.2025.07.022. Epub 2025 Jul 29.

PMID- 40772222
OWN - NLM
STAT- PubMed-not-MEDLINE
LR - 20250809
IS - 2168-8184 (Print)
IS - 2168-8184 (Electronic)
IS - 2168-8184 (Linking)
VI - 17
IP - 7
DP - 2025 Jul
TI - Minimally Invasive Techniques for Managing Dental Caries in
Children: Efficacy,
      Applications, and Future Directions.
PG - e87450
LID - 10.7759/cureus.87450 [doi]
LID - e87450
AB - Minimally Invasive Dentistry (MID) represents a transformative
approach in
      pediatric caries management, emphasizing tooth preservation,
patient comfort, and
      preventive care over conventional invasive methods. The aim of this
narrative
      review is to evaluate¬†the efficacy, clinical applications, and
future directions
      of various MID techniques in children, including silver diamine
fluoride (SDF),
      atraumatic restorative treatment (ART), the Hall Technique, resin
infiltration,
      and bioactive restorative materials. These methods have shown
considerable
      success in reducing treatment complexity and improving outcomes,
especially for
      high-risk and special healthcare needs (SHCN) populations. A
structured
      literature search was conducted across PubMed, Scopus, and Google
Scholar
      databases for studies published between 2000 and 2025. Search terms
included
      "minimally invasive dentistry in children," "silver diamine
fluoride pediatric
      caries," "atraumatic restorative treatment," and related
techniques. Inclusion
      criteria encompassed clinical trials, systematic reviews, and
expert consensus
      articles, while animal studies and those lacking rigorous
methodology were
      excluded. Results indicate that MID techniques such as SDF can
arrest caries in
      80-90% of cases, while ART and the Hall Technique provide minimally
invasive,
      cost-effective alternatives to conventional restorations. Resin
infiltration

improves esthetics and arrests lesion progression in non-cavitated teeth, while bioactive materials promote remineralization and pulp preservation. Emerging technologies, including laser-assisted caries removal, air abrasion, and AI-driven diagnostics, further enhance MID accessibility and precision. Despite promising outcomes, challenges such as clinician training deficits, parental concerns, and lack of long-term evidence persist. Future efforts should focus on addressing these barriers through research, education, and policy reform to facilitate the integration of MID into routine pediatric dental practice.

CI - Copyright ¬© 2025, AL-Kaff et al.

FAU - Al-Kaff, Arwa A
AU - Al-Kaff AA
AD - Pediatric Dentistry, Ministry of Health, Riyadh First Health Cluster, Riyadh, SAU.
FAU - Alshehri, Abdulrahman Z
AU - Alshehri AZ
AD - Dentistry, College of Dentistry, King Saud University, Riyadh, SAU.
FAU - Alasmari, Rana A
AU - Alasmari RA
AD - Dentistry, Princess Nourah bint Abdulrahman University, Riyadh, SAU.
FAU - Alsubaie, Najd
AU - Alsubaie N
AD - Dentistry, Princess Nourah bint Abdulrahman University, Riyadh, SAU.
FAU - Aldaws, Albandari
AU - Aldaws A
AD - Dentistry, Princess Nourah Bint Abdulrahman University, Riyadh , SAU.
FAU - Althaqeel, Ameera
AU - Althaqeel A
AD - Dentistry, Riyadh Elm University, Riyadh , SAU.
FAU - Alshehri, Rola S
AU - Alshehri RS
AD - General Dentistry, Princess Nourah bint Abdulrahman University, Riyadh, SAU.
FAU - Alawaji, Yasmin M
AU - Alawaji YM
AD - Dentistry, Princess Nourah bint Abdulrahman University, Riyadh, SAU.

LA - eng
PT - Journal Article
PT - Review
DEP - 20250707
PL - United States
TA - Cureus

JT - Cureus
JID - 101596737
PMC - PMC12327548
OTO - NOTNLM
OT - atraumatic restorative treatment
OT - bioactive materials
OT - caries management
OT - hall technique
OT - minimally invasive dentistry
OT - pediatric dentistry
OT - resin infiltration
OT - silver diamine fluoride (sdf)
OT - teledentistry
COIS- Conflicts of interest: In compliance with the ICMJE uniform disclosure form, all
      authors declare the following: Payment/services info: All authors have declared
      that no financial support was received from any organization for the submitted
      work. Financial relationships: All authors have declared that they have no
      financial relationships at present or within the previous three years with any
      organizations that might have an interest in the submitted work. Other
      relationships: All authors have declared that there are no other relationships or
      activities that could appear to have influenced the submitted work.
EDAT- 2025/08/07 11:43
MHDA- 2025/08/07 11:44
PMCR- 2025/07/07
CRDT- 2025/08/07 05:15
PHST- 2025/07/06 00:00 [accepted]
PHST- 2025/08/07 11:44 [medline]
PHST- 2025/08/07 11:43 [pubmed]
PHST- 2025/08/07 05:15 [entrez]
PHST- 2025/07/07 00:00 [pmc-release]
AID - 10.7759/cureus.87450 [doi]
PST - epublish
SO - Cureus. 2025 Jul 7;17(7):e87450. doi: 10.7759/cureus.87450. eCollection 2025 Jul.

PMID- 40618744
OWN - NLM
STAT- Publisher
LR - 20250827
IS - 1421-976X (Electronic)
IS - 0008-6568 (Linking)
DP - 2025 Jul 4
TI - Reliability of an Artificial Intelligence Software in the Detection of Approximal
      Caries Lesions Using Bitewing Radiographs.
PG - 1-8
LID - 10.1159/000547245 [doi]

AB - INTRODUCTION: This study evaluates the reliability of an artificial intelligence
      (AI) software for detecting approximal caries lesions of different depth on
      bitewing radiographs. METHODS: A total of 40 bitewing radiographs consisting of
      288 teeth (576 approximal surfaces) were selected for analysis. Utilising the
      International Caries Detection and Assessment System radiographic scoring system,
      five dentists established a consensus on the assessment of all radiographs,
      serving as the gold standard of this study. These radiographs were later analysed
      using an AI software (Nostic software¬Æ), and the detection results were compared
      to the established ground truth. RESULTS: The area under the curve (AUC),
      accuracy, sensitivity, specificity, positive predictive values, negative
      predictive values, and F1 scores were computed. A total of 246 surfaces were
      included for the detection of enamel lesions (D (1-2)) while 341 surfaces were
      assed for dentinal lesions (D (3-4)) and for both enamel and dentinal lesions (D
      (1-4)). The accuracy (95% confidence interval) for detecting enamel lesions (D
      (1-2)) was 0.78 (0.72-0.83), for dentinal lesions (D (3-4)) was 0.85 (0.80-0.88)
      and for both enamel and dentinal lesions (D (1-4)) was 0.77 (0.73-0.81).
      Correspondingly, the AUC (95% confidence interval) values for detecting enamel
      lesions (D (1-2)), dentinal lesion (D (3-4)) and both enamel and dentinal lesions
      (D (1-4)) were 0.70 (0.65-0.76), 0.81 (0.75-0.87), 0.75 (0.71-0.80),
      respectively. CONCLUSION: In conclusion, the performance of the AI software in
      detecting proximal caries lesions of varying depths on bitewing radiographs was
      found to be decent when compared to the gold standard. This AI software has the
      potential to serve as an effective tool to support diagnosing initial caries in
      bitewing images for dental practitioners.
CI - ¬© 2025 S. Karger AG, Basel.
FAU - Piipari, Liina
AU - Piipari L
AD - University of Oulu, Research Unit of Population Health, Oulu, Finland.
FAU - Anttonen, Vuokko
AU - Anttonen V

AD - University of Oulu, Research Unit of Population Health, Oulu, Finland.
FAU - Lussi, Adrian
AU - Lussi A
AD - University Hospital for Conservative Dentistry and Periodontology, Medical
      University of Innsbruck, Innsbruck, Austria.
AD - Department of Restorative, Preventive and Pediatric Dentistry, School of Dental
      Medicine, Bern, Switzerland.
FAU - Laitala, Marja-Liisa
AU - Laitala ML
AD - University of Oulu, Research Unit of Population Health, Oulu, Finland.
AD - University of Oulu, Medical Research Center, Oulu, Finland.
FAU - Tanner, Tarja
AU - Tanner T
AD - University of Oulu, Research Unit of Population Health, Oulu, Finland.
FAU - Karki, Saujanya
AU - Karki S
AD - University of Oulu, Research Unit of Population Health, Oulu, Finland.
LA - eng
PT - Journal Article
DEP - 20250704
PL - Switzerland
TA - Caries Res
JT - Caries research
JID - 0103374
SB - IM
OTO - NOTNLM
OT - Artificial intelligence
OT - Bitewing radiograph
OT - Caries detection
OT - Deep learning
OT - Enamel caries
EDAT- 2025/07/07 00:26
MHDA- 2025/07/07 00:26
CRDT- 2025/07/06 18:23
PHST- 2024/10/06 00:00 [received]
PHST- 2025/06/16 00:00 [accepted]
PHST- 2025/07/07 00:26 [pubmed]
PHST- 2025/07/07 00:26 [medline]
PHST- 2025/07/06 18:23 [entrez]
AID - 000547245 [pii]
AID - 10.1159/000547245 [doi]
PST - aheadofprint
SO - Caries Res. 2025 Jul 4:1-8. doi: 10.1159/000547245.

PMID- 40565492
OWN - NLM
STAT- PubMed-not-MEDLINE
LR - 20250628

IS - 2227-9032 (Print)
IS - 2227-9032 (Electronic)
IS - 2227-9032 (Linking)
VI - 13
IP - 12
DP - 2025 Jun 18
TI - Deep Learning Applications in Dental Image-Based Diagnostics: A Systematic
      Review.
LID - 10.3390/healthcare13121466 [doi]
LID - 1466
AB - Background: AI has been adopted in dentistry for diagnosis, decision making, and
      therapy prognosis prediction. This systematic review aimed to identify AI models
      in dentistry, assess their performance, identify their shortcomings, and discuss
      their potential for adoption and integration in dental practice in the future.
      Methodology: The sources of the papers were the following electronic databases:
      PubMed, Scopus, and Cochrane Library. A total of 20 out of 947 needed further
      studies, and this was encompassed in the present meta-analysis. It identified
      diagnostic accuracy, predictive performance, and potential biases. Results: AI
      models demonstrated an overall diagnostic accuracy of 82%, primarily leveraging
      artificial neural networks (ANNs) and convolutional neural networks (CNNs). These
      models have significantly improved the diagnostic precision for dental caries
      compared with traditional methods. Moreover, they have shown potential in
      detecting and managing conditions such as bone loss, malignant lesions, vertical
      root fractures, apical lesions, salivary gland disorders, and maxillofacial
      cysts, as well as in performing orthodontic assessments. However, the integration
      of AI systems into dentistry poses challenges, including potential data biases,
      cost implications, technical requirements, and ethical concerns such as patient
      data security and informed consent. AI models may also underperform when faced
      with limited or skewed datasets, thus underscoring the importance of robust
      training and validation procedures. Conclusions: AI has the potential to
      revolutionize dentistry by significantly improving diagnostic accuracy and

treatment planning. However, before integrating this tool into clinical practice, a critical assessment of its advantages, disadvantages, and utility or ethical issues must be established. Future studies should aim to eradicate existing barriers and enhance the model's ease of understanding and challenges regarding expense and data protection, to ensure the effective utilization of AI in dental healthcare.

FAU - Khattak, Osama
AU - Khattak O
AUID- ORCID: 0000-0003-3502-3555
AD - Department of Restorative Dentistry, College of Dentistry, Jouf University, Sakaka 72311, Saudi Arabia.
FAU - Hashem, Ahmed Shawkat
AU - Hashem AS
AD - Oral Medicine and Periodontology, Faculty of Dentistry, Damanhour University, Damanhur 22522, Egypt.
FAU - Alqarni, Mohammed Saad
AU - Alqarni MS
AUID- ORCID: 0000-0002-6368-0774
AD - Department of Oral & Maxillofacial Surgery and Diagnostic Sciences, College of Dentistry, Jouf University, Sakaka 72311, Saudi Arabia.
FAU - Almufarrij, Raha Ahmed Shamikh
AU - Almufarrij RAS
AUID- ORCID: 0009-0002-3152-7270
AD - College of Dentistry, Jouf University, Sakaka 72311, Saudi Arabia.
FAU - Siddiqui, Amna Yusuf
AU - Siddiqui AY
AUID- ORCID: 0000-0003-0817-7129
AD - Department of Endodontics, Faculty of Dentistry, King Abdulaziz University, Jeddah 22230, Saudi Arabia.
FAU - Anis, Rabia
AU - Anis R
AD - Department of Health Professions Education, Isra Dental College, Isra University, Hydrabad 73000, Pakistan.
FAU - Ahmad, Shahzad
AU - Ahmad S
AD - Faculty of Medicine and Health Science, The University of Buckingham, Buckingham MK18 1EG, UK.
FAU - Fareed, Muhammad Amber
AU - Fareed MA
AUID- ORCID: 0000-0001-8440-0092
AD - Clinical Sciences Department, College of Dentistry, Ajman University, Ajman 346, United Arab Emirates.

AD - Centre of Medical and Bio-Allied Health Sciences Research, Ajman University,
      Ajman 346, United Arab Emirates.
FAU - Alothmani, Osama Shujaa
AU - Alothmani OS
AUID- ORCID: 0000-0002-9552-1305
AD - Department of Endodontics, Faculty of Dentistry, King Abdulaziz University,
      Jeddah 22230, Saudi Arabia.
FAU - Alkhershawy, Lama Habis Samah
AU - Alkhershawy LHS
AUID- ORCID: 0009-0001-8335-1062
AD - College of Dentistry, Jouf University, Sakaka 72311, Saudi Arabia.
FAU - Alabidin, Wesam Waleed Zain
AU - Alabidin WWZ
AD - College of Dentistry, Jouf University, Sakaka 72311, Saudi Arabia.
FAU - Issrani, Rakhi
AU - Issrani R
AD - Department of Preventive Dentistry, College of Dentistry, Jouf University, Sakaka
      72311, Saudi Arabia.
FAU - Agarwal, Anshoo
AU - Agarwal A
AD - Department of Pathology, Northern Border University, Arar 91431, Saudi Arabia.
LA - eng
GR - This research is funded by the Deanship of Graduate Studies and Scientific
      Research at Jouf Uni-versity by the funding of Fast-Track Research./Jouf
      University/
PT - Journal Article
PT - Review
DEP - 20250618
PL - Switzerland
TA - Healthcare (Basel)
JT - Healthcare (Basel, Switzerland)
JID - 101666525
PMC - PMC12193449
OTO - NOTNLM
OT - AI models
OT - artificial intelligence
OT - dental sciences
OT - diagnosis
OT - machine learning
COIS- The authors declare no conflicts of interest.
EDAT- 2025/06/26 06:27
MHDA- 2025/06/26 06:28
PMCR- 2025/06/18
CRDT- 2025/06/26 01:14
PHST- 2025/05/05 00:00 [received]
PHST- 2025/06/06 00:00 [revised]
PHST- 2025/06/09 00:00 [accepted]
PHST- 2025/06/26 06:28 [medline]

PHST- 2025/06/26 06:27 [pubmed]
PHST- 2025/06/26 01:14 [entrez]
PHST- 2025/06/18 00:00 [pmc-release]
AID - healthcare13121466 [pii]
AID - healthcare-13-01466 [pii]
AID - 10.3390/healthcare13121466 [doi]
PST - epublish
SO - Healthcare (Basel). 2025 Jun 18;13(12):1466. doi:
10.3390/healthcare13121466.

PMID- 40560354
OWN - NLM
STAT- Publisher
LR - 20250625
IS - 1996-9805 (Electronic)
IS - 1818-6300 (Linking)
DP - 2025 Jun 25
TI - Are mobile health applications for traumatic dental injuries
effective? A
systematic review of their impact on diagnosis, prevention,
management, and
education.
LID - 10.1007/s40368-025-01071-0 [doi]
AB - PURPOSE: Mobile health (mHealth) applications (apps) have emerged
as powerful
tools for enhancing clinical decision-making, knowledge
dissemination, and
proactive care in traumatic dental injuries (TDIs). This systematic
review aims
to collate and assess the available evidence on the usefulness of
mobile apps in
(1) diagnosis, (2) prevention, (3) management, and (4) education of
TDIs.
METHODS: This review examined studies published between January
2012 and August
2024, focusing on randomised controlled trials (RCTs), clinical
trials, cohort
studies, and cross-sectional studies that assessed the
effectiveness of mobile
health apps in the management of TDI care. Study quality was
assessed using the
Cochrane Risk of Bias Tool 2.0 for RCTs and the modified Newcastle-
Ottawa Scale
for cross-sectional studies. The GRADE approach was used to assess
the certainty
of the evidence. RESULTS: Sixteen studies met the inclusion
criteria, including
12 cross-sectional studies and 4 RCTs. The cross-sectional studies
primarily
assessed knowledge improvement, while the RCTs focused on skill
development and
practical applications. Quality assessments indicated notable
methodological

concerns. All four RCTs were rated as high risk of bias due to inadequate
randomisation, lack of blinding, and participant attrition. According to the
GRADE approach, the overall certainty of the evidence was judged to be very low.
CONCLUSION: While mobile health applications such as ToothSOS, Dental Trauma, and
Injured Tooth show promise in improving knowledge and decision-making related to
TDIs, the evidence is limited by high risk of bias and low quality. Future
studies should focus on rigorous RCTs and explore AI integration to enhance
effectiveness.
CI - ¬© 2025. The Author(s), under exclusive licence to European Academy of Paediatric
Dentistry.
FAU - Walia, T
AU - Walia T
AD - College of Dentistry, Ajman University, Ajman, United Arab Emirates.
ttww@yahoo.com.
FAU - Muthu, M S
AU - Muthu MS
AD - Centre for Early Childhood Caries Research (CECCRe), Department of Pediatric and
Preventive Dentistry, Sri Ramachandra Faculty of Dental Sciences, Sri Ramachandra
Institute of Higher Education and Research (SRIHER), Porur, Chennai, 600116,
India.
FAU - Saikia, A
AU - Saikia A
AD - Department of Paediatric Dentistry, UWA Dental School, The University of Western
Australia, 17 Monash Ave, Nedlands, Perth, 6009, Australia.
FAU - Shetty, R M
AU - Shetty RM
AD - College of Dentistry, Ajman University, Ajman, United Arab Emirates.
AD - Department of Pediatric and Preventive Dentistry, Sharad Pawar Dental College and
Hospital, Datta Meghe Institute of Higher Education and Research (Declared as
Deemed-to-be University), Wardha, Maharashtra, India.
FAU - Anthonappa, R P
AU - Anthonappa RP
AD - Department of Paediatric Dentistry, UWA Dental School, The University of Western
Australia, 17 Monash Ave, Nedlands, Perth, 6009, Australia.
LA - eng
PT - Journal Article
PT - Systematic Review

DEP - 20250625
PL - England
TA - Eur Arch Paediatr Dent
JT - European archives of paediatric dentistry : official journal of the European
      Academy of Paediatric Dentistry
JID - 101277157
SB - IM
OTO - NOTNLM
OT - Diagnosis
OT - Education
OT - Healthcare professionals
OT - Management
OT - Mobile applications
OT - Prevention
OT - Systematic review
OT - Traumatic dental injuries
OT - mHealth
COIS- Declarations. Conflict of interest: The authors declare no competing interests.
      Ethical approval: This is a systematic review. Hence, ethical approval was not
      required.
EDAT- 2025/06/25 12:28
MHDA- 2025/06/25 12:28
CRDT- 2025/06/25 11:10
PHST- 2025/03/12 00:00 [received]
PHST- 2025/05/28 00:00 [accepted]
PHST- 2025/06/25 12:28 [medline]
PHST- 2025/06/25 12:28 [pubmed]
PHST- 2025/06/25 11:10 [entrez]
AID - 10.1007/s40368-025-01071-0 [pii]
AID - 10.1007/s40368-025-01071-0 [doi]
PST - aheadofprint
SO - Eur Arch Paediatr Dent. 2025 Jun 25. doi: 10.1007/s40368-025-01071-0.

PMID- 40507004
OWN - NLM
STAT- PubMed-not-MEDLINE
LR - 20250615
IS - 2075-4418 (Print)
IS - 2075-4418 (Electronic)
IS - 2075-4418 (Linking)
VI - 15
IP - 11
DP - 2025 Jun 4
TI - Assessment of the Diagnostic Accuracy of Artificial Intelligence Software in
      Identifying Common Periodontal and Restorative Dental Conditions (Marginal Bone
      Loss, Periapical Lesion, Crown, Restoration, Dental Caries) in Intraoral
      Periapical Radiographs.

LID - 10.3390/diagnostics15111432 [doi]
LID - 1432
AB - Objectives: The purpose of the study is to evaluate the diagnostic accuracy of
artificial intelligence (AI) software in detecting a common set of periodontal
and restorative conditions, including marginal bone loss, dental caries,
periapical lesions, calculus, endodontic treatment, crowns, restorations, and
open crown margins, using intraoral periapical radiographs. Additionally, the
study will assess how this AI software influences the diagnostic accuracy of
dentists with varying levels of experience in identifying these conditions.
Methods: A total of three hundred digital IOPARs representing 1030 teeth were
selected based on predetermined selection criteria. The parameters assessed
included (a) calculus, (b) periapical radiolucency, (c) caries, (d) marginal bone
loss, (e) type of restorative (filling) material, (f) type of crown retainer
material, and (g) detection of open crown margins. Two oral radiologists
performed the initial diagnosis of the selected radiographs and independently
labeled all the predefined parameters for the provided IOPARs under standardized
conditions. This data served as reference data. A pre-trained AI-based
computer-aided detection ("CADe") software (Second Opinion(¬Æ), version 1.1) was
used for the detection of the predefined features. The reports generated by the
AI software were compared with the reference data to evaluate the diagnostic
accuracy of the AI software. In the second phase of the study, thirty dental
interns and thirty dental specialists were randomly selected. Each participant
was randomly assigned five IOPARs and was asked to detect and diagnose the
predefined conditions. Subsequently, all the participants were requested to
reassess the IOPARs, this time with the assistance of the AI software. All the
data was recorded using a self-designed Performa. Results: The sensitivity of the
AI software in detecting caries, periapical lesions, crowns, open crown margins,
restoration, endodontic treatment, calculus, and marginal bone loss was 91.0%,

86.6%, 97.1%, 82.6%, 89.3%, 93.4%, 80.2%, and 91.1%, respectively. The
specificity of the AI software in detected caries, periapical lesions, crowns,
open crown margins, restoration, endodontic treatment, calculus, and marginal
bone loss was 87%, 98.3%, 99.6%, 91.9%, 96.4%, 99.3%, 97.8%, and 93.1%,
respectively. The differences between the AI software and radiologist diagnoses
of caries, periapical lesions, crowns, open crown margins, restoration,
endodontic treatment, calculus, and marginal bone loss were statistically
significant (all p values < 0.0001). The results showed that the diagnostic
accuracy of operators (interns and specialists) with AI software revealed higher
accuracy, sensitivity, and specificity in detecting caries, PA lesions,
restoration, endodontic treatment, calculus, and marginal bone loss compared to
that without using AI software. There were variations in the improvements in the
diagnostic accuracy of interns and dental specialists. Conclusions: Within the
limitations of the study, it can be concluded that the tested AI software has
high accuracy in detecting the tested dental conditions in IOPARs. The use of AI
software enhanced the diagnostic capabilities of dental operators. The present
study used AI software to detect a clinically useful set of periodontal and
restorative conditions, which can help dental operators in fast and accurate
diagnosis and provide high-quality treatment to their patients.
FAU - Ibraheem, Wael I
AU - Ibraheem WI
AUID- ORCID: 0000-0003-0933-5459
AD - Department of Preventive Dental Sciences, College of Dentistry, Jazan University,
Jazan 45142, Saudi Arabia.
FAU - Jain, Saurabh
AU - Jain S
AUID- ORCID: 0000-0002-0071-4068
AD - Department of Prosthetic Dental Sciences, College of Dentistry, Jazan University,
Jazan 45142, Saudi Arabia.
FAU - Ayoub, Mohammed Naji
AU - Ayoub MN
AUID- ORCID: 0009-0007-6118-4834
AD - Intern Clinic, College of Dentistry, Jazan University, Jazan 45142, Saudi Arabia.

FAU - Namazi, Mohammed Ahmed
AU - Namazi MA
AUID- ORCID: 0009-0009-8764-5402
AD - Intern Clinic, College of Dentistry, Jazan University, Jazan 45142, Saudi Arabia.
FAU - Alfaqih, Amjad Ismail
AU - Alfaqih AI
AUID- ORCID: 0009-0007-3932-4263
AD - Intern Clinic, College of Dentistry, Jazan University, Jazan 45142, Saudi Arabia.
FAU - Aggarwal, Aparna
AU - Aggarwal A
AUID- ORCID: 0000-0003-0358-9474
AD - Vitaldent Advanced Dental Clinic, Faridabad 121002, India.
FAU - Meshni, Abdullah A
AU - Meshni AA
AD - Department of Prosthetic Dental Sciences, College of Dentistry, Jazan University,
      Jazan 45142, Saudi Arabia.
FAU - Almarghlani, Ammar
AU - Almarghlani A
AD - Department of Periodontics, Faculty of Dentistry, King Abdulaziz University,
      Jeddah 21589, Saudi Arabia.
FAU - Alhumaidan, Abdulkareem Abdullah
AU - Alhumaidan AA
AD - Department of Preventive Dental Sciences, College of Dentistry, Imam Abdulrahman
      Bin Faisal University, Dammam 31441, Saudi Arabia.
LA - eng
PT - Journal Article
DEP - 20250604
PL - Switzerland
TA - Diagnostics (Basel)
JT - Diagnostics (Basel, Switzerland)
JID - 101658402
PMC - PMC12154273
OTO - NOTNLM
OT - artificial intelligence
OT - calculus
OT - convolutional neural network (CNN)
OT - deep learning
OT - dental caries
OT - dental crown
OT - diagnosis
OT - diagnostic imaging
OT - digital imaging/radiology
OT - intraoral radiographs
OT - machine learning
OT - marginal bone loss
OT - periapical lesion
OT - restoration
COIS- The authors declare no conflicts of interest.
EDAT- 2025/06/13 06:35

MHDA- 2025/06/13 06:36
PMCR- 2025/06/04
CRDT- 2025/06/13 01:01
PHST- 2025/05/15 00:00 [received]
PHST- 2025/05/30 00:00 [revised]
PHST- 2025/06/04 00:00 [accepted]
PHST- 2025/06/13 06:36 [medline]
PHST- 2025/06/13 06:35 [pubmed]
PHST- 2025/06/13 01:01 [entrez]
PHST- 2025/06/04 00:00 [pmc-release]
AID - diagnostics15111432 [pii]
AID - diagnostics-15-01432 [pii]
AID - 10.3390/diagnostics15111432 [doi]
PST - epublish
SO - Diagnostics (Basel). 2025 Jun 4;15(11):1432. doi:
10.3390/diagnostics15111432.

PMID- 40463673
OWN - NLM
STAT- PubMed-not-MEDLINE
LR - 20250606
IS - 2950-4708 (Electronic)
IS - 2950-4716 (Print)
IS - 2950-4708 (Linking)
VI - 28
IP - 5
DP - 2025 May
TI - Revolutionizing the diagnosis of dental caries using artificial
      intelligence-based methods.
PG - 401-405
LID - 10.4103/JCDE.JCDE_172_25 [doi]
AB - Early diagnosis and carious lesion detection through artificial intelligence (AI)
      have transformed current standard methodologies because it generates precise
      results which work more efficiently and dependably. AI uses machine learning and
      deep learning technologies with computer-aided diagnostic systems to accomplish
      exceptional image evaluation of radiographic data and clinical records in dental
      caries detection through intraoral scans. This review discusses both prevailing
      challenges which limit and potential future uses of AI in dental diagnosis
      together with its ability to become a part of standard clinical work routines.
      Various researchers confirmed that AI works as a helpful tool which supports
      dental experts by improving diagnosis and minimizing human biases to enhance
      preventive care effects for patients.
CI - Copyright: ¬© 2025 Journal of Conservative Dentistry and Endodontics.

FAU - Marwaha, Jasmine
AU - Marwaha J
AD - Department of Conservative Dentistry and Endodontics, SGT Dental College,
      Hospital and Research Institute, SGT University, Gurugram, Haryana, India.
FAU - Singla, Mamta
AU - Singla M
AD - Department of Conservative Dentistry and Endodontics, SGT Dental College,
      Hospital and Research Institute, SGT University, Gurugram, Haryana, India.
FAU - Nath, Amar
AU - Nath A
AD - Department of Computer Sciences and Engineering, Sant Longowal Institute of
      Engineering and Technology, Sangrur, Punjab, India.
FAU - Arya, Ashtha
AU - Arya A
AD - Department of Conservative Dentistry and Endodontics, SGT Dental College,
      Hospital and Research Institute, SGT University, Gurugram, Haryana, India.
LA - eng
PT - Journal Article
PT - Review
DEP - 20250506
PL - India
TA - J Conserv Dent Endod
JT - Journal of conservative dentistry and endodontics
JID - 9918697474706676
PMC - PMC12129285
OTO - NOTNLM
OT - Artificial intelligence in dentistry
OT - artificial intelligence
OT - computer-aided diagnosis
OT - deep learning
OT - dental caries detection
OT - machine learning
OT - preventive dentistry
COIS- There are no conflicts of interest.
EDAT- 2025/06/04 06:27
MHDA- 2025/06/04 06:28
PMCR- 2025/05/01
CRDT- 2025/06/04 05:04
PHST- 2025/03/13 00:00 [received]
PHST- 2025/03/24 00:00 [revised]
PHST- 2025/04/10 00:00 [accepted]
PHST- 2025/06/04 06:28 [medline]
PHST- 2025/06/04 06:27 [pubmed]
PHST- 2025/06/04 05:04 [entrez]
PHST- 2025/05/01 00:00 [pmc-release]
AID - JCDE-28-401 [pii]
AID - 10.4103/JCDE.JCDE_172_25 [doi]

PST - ppublish
SO - J Conserv Dent Endod. 2025 May;28(5):401-405. doi:
10.4103/JCDE.JCDE_172_25. Epub
      2025 May 6.

PMID- 40434067
OWN - NLM
STAT- Publisher
LR - 20250528
IS - 2035-648X (Electronic)
IS - 1591-996X (Linking)
DP - 2025 May 1
TI - Application of machine learning for data analysis in paediatric
dentistry: a
      systematic review.
PG - 1
LID - 10.23804/ejpd.2025.2288 [doi]
AB - AIM: The study aims to assess whether the application of machine
learning (ML)
      for database analysis enhances the approach to oral diseases in the
paediatric
      population. MATERIALS: Dental caries affects 514 million children
worldwide.
      Artificial intelligence (AI), particularly ML, has seen increased
utilisation in
      medicine and dentistry, handling data beyond human capacity to
discern patterns
      and make predictions. PubMed, Web of Science, Scopus, and Lilacs
databases were
      searched. Topics covered include the impact of oral health on
adolescents'
      quality of life, predictors of early childhood caries and of the
need of second
      treatment under deep sedation, and the effectiveness of preventive
dental
      services. METHODS: Twenty articles meeting eligibility criteria
were analyzed for
      quality using the QUADAS-2 scale. The systematic review adhered to
the PRISMA
      statement, yielding 20 articles out of 1945 initially screened.
Fourteen articles
      focused on caries prediction, highlighting socio-demographic,
behavioural, and
      biological predictors. ML analysis revealed that children with
early caries
      lesions incur higher costs for insurers, with those receiving
sealants and
      fluoride demonstrating greater cost savings. CONCLUSION: ML
algorithms can
      identify patterns in large datasets, enhancing approaches to
paediatric oral
      diseases. Their integration into research and educational programs
is

recommended. Methodological guidelines and quality scales specific to such
studies are necessary for improved scientific evidence.
FAU - G√≥mez-R√≠os, I
AU - G√≥mez-R√≠os I
AD - Universidad de Murcia.
FAU - Saura-L√≥pez, V
AU - Saura-L√≥pez V
AD - Universidad de Murcia.
FAU - P√©rez-Silva, A
AU - P√©rez-Silva A
AD - Universidad de Murcia.
FAU - Serna-Mu√±oz, C
AU - Serna-Mu√±oz C
AD - Universidad de Murcia.
FAU - Ortiz-Ruiz, A J
AU - Ortiz-Ruiz AJ
AD - Universidad de Murcia.
LA - eng
PT - Journal Article
DEP - 20250501
PL - Italy
TA - Eur J Paediatr Dent
JT - European journal of paediatric dentistry
JID - 101121881
SB - IM
EDAT- 2025/05/28 12:29
MHDA- 2025/05/28 12:29
CRDT- 2025/05/28 08:56
PHST- 2025/05/28 12:29 [medline]
PHST- 2025/05/28 12:29 [pubmed]
PHST- 2025/05/28 08:56 [entrez]
AID - 10.23804/ejpd.2025.2288 [doi]
PST - aheadofprint
SO - Eur J Paediatr Dent. 2025 May 1:1. doi: 10.23804/ejpd.2025.2288.

PMID- 40354695
OWN - NLM
STAT- MEDLINE
DCOM- 20250716
LR - 20250716
IS - 1875-595X (Electronic)
IS - 0020-6539 (Print)
IS - 0020-6539 (Linking)
VI - 75
IP - 4
DP - 2025 Aug
TI - AI-Driven Dental Caries Management Strategies: From Clinical Practice to
Professional Education and Public Self Care.
PG - 100827
LID - S0020-6539(25)00116-9 [pii]
LID - 10.1016/j.identj.2025.04.007 [doi]
LID - 100827

AB - Dental caries is one of the most prevalent chronic diseases among both children
      and adults, despite being largely preventable. This condition has significant
      negative impacts on human health and imposes a substantial economic burden. In
      recent years, scientists and dentists have increasingly started to utilize
      artificial intelligence (AI), particularly machine learning, to improve the
      efficiency of dental caries management. This study aims to provide an overview of
      the current knowledge about the AI-enabled approaches for dental caries
      management within the framework of personalized patient care. Generally, AI works
      as a promising tool that can be used by both dental professionals and patients.
      For dental professionals, it predicts the risk of dental caries by analyzing
      dental caries risk and protective factors, enabling to formulate personalized
      preventive measures. AI, especially those based on machine learning and deep
      learning, can also analyze images to detect signs of dental caries, assist in
      developing treatment plans, and help to make a risk assessment for pulp exposure
      during treatment. AI-powered tools can also be used to train dental students
      through simulations and virtual case studies, allowing them to practice and
      refine their clinical skills in a risk-free environment. Additionally, AI tracks
      brushing patterns and provides feedback to improve oral hygiene practices of the
      patients and the general population, thereby improving their understanding and
      compliance. This capability of AI can inform future research and the development
      of new strategies for dental caries management and control.
CI - Copyright ¬© 2025 The Authors. Published by Elsevier Inc. All rights reserved.
FAU - Liang, Yutong
AU - Liang Y
AD - State Key Laboratory of Oral Diseases & National Center for Stomatology &
      National Clinical Research Center for Oral Diseases, West China Hospital of
      Stomatology, Sichuan University, Chengdu, Sichuan, China; Department of Cariology
      and Endodontics, West China Hospital of Stomatology, Sichuan University, Chengdu,
      China.

FAU - Li, Dongling
AU - Li D
AD - State Key Laboratory of Oral Diseases & National Center for Stomatology &
      National Clinical Research Center for Oral Diseases, West China Hospital of
      Stomatology, Sichuan University, Chengdu, Sichuan, China; Department of Cariology
      and Endodontics, West China Hospital of Stomatology, Sichuan University, Chengdu,
      China.
FAU - Deng, Dongmei
AU - Deng D
AD - Department of Preventive Dentistry, Academic Center for Dentistry Amsterdam
      (ACTA), University of Amsterdam and Vrije Universiteit Amsterdam, Amsterdam, The
      Netherlands.
FAU - Chu, Chun Hung
AU - Chu CH
AD - Faculty of Dentistry, University of Hong Kong, Hong Kong, China.
FAU - Mei, May Lei
AU - Mei ML
AD - Sir John Walsh Research Institute, Faculty of Dentistry, University of Otago,
      Dunedin, New Zealand.
FAU - Li, Yunpeng
AU - Li Y
AD - Centre for Oral, Clinical and Translational Sciences, Faculty of Dental, Oral and
      Craniofacial Sciences, King's College London, London, United Kingdom.
FAU - Yu, Na
AU - Yu N
AD - National Dental Centre Singapore, Singapore.
FAU - He, Jinzhi
AU - He J
AD - State Key Laboratory of Oral Diseases & National Center for Stomatology &
      National Clinical Research Center for Oral Diseases, West China Hospital of
      Stomatology, Sichuan University, Chengdu, Sichuan, China; Department of Cariology
      and Endodontics, West China Hospital of Stomatology, Sichuan University, Chengdu,
      China. Electronic address: hejinzhi@scu.edu.cn.
FAU - Cheng, Lei
AU - Cheng L
AD - State Key Laboratory of Oral Diseases & National Center for Stomatology &
      National Clinical Research Center for Oral Diseases, West China Hospital of
      Stomatology, Sichuan University, Chengdu, Sichuan, China; Department of Cariology

```
      and Endodontics, West China Hospital of Stomatology, Sichuan
University, Chengdu,
      China. Electronic address: chenglei@scu.edu.cn.
LA  - eng
PT  - Journal Article
PT  - Review
DEP - 20250510
PL  - England
TA  - Int Dent J
JT  - International dental journal
JID - 0374714
SB  - IM
MH  - Humans
MH  - *Dental Caries/therapy/prevention & control
MH  - *Artificial Intelligence
MH  - *Self Care
MH  - *Education, Dental
MH  - Oral Hygiene
PMC - PMC12138923
OTO - NOTNLM
OT  - Artificial intelligence
OT  - Dental caries
OT  - Diagnosis and therapeutics
OT  - Education, Dental
OT  - Risk assessment
OT  - Self care
COIS- Declaration of competing interest The authors declare that they
have no known
      competing financial interests or personal relationships that could
have appeared
      to influence the work reported in this paper.
EDAT- 2025/05/12 18:40
MHDA- 2025/07/17 04:41
PMCR- 2025/05/10
CRDT- 2025/05/12 17:59
PHST- 2025/01/11 00:00 [received]
PHST- 2025/04/13 00:00 [revised]
PHST- 2025/04/14 00:00 [accepted]
PHST- 2025/07/17 04:41 [medline]
PHST- 2025/05/12 18:40 [pubmed]
PHST- 2025/05/12 17:59 [entrez]
PHST- 2025/05/10 00:00 [pmc-release]
AID - S0020-6539(25)00116-9 [pii]
AID - 100827 [pii]
AID - 10.1016/j.identj.2025.04.007 [doi]
PST - ppublish
SO  - Int Dent J. 2025 Aug;75(4):100827. doi:
10.1016/j.identj.2025.04.007. Epub 2025
      May 10.

PMID- 40417449
OWN - NLM
STAT- PubMed-not-MEDLINE
LR  - 20250527
```

IS - 0974-7052 (Print)
IS - 0975-1904 (Electronic)
IS - 0974-7052 (Linking)
VI - 18
IP - 2
DP - 2025 Feb
TI - Assessment of Compliance and Validity of Digital Application-based Diet Diary
with Suggestive Local Food Habits for Prevention of Dental Caries in Children.
PG - 191-197
LID - 10.5005/jp-journals-10005-3047 [doi]
AB - AIM: The purpose of the study is to use a community-based participatory research
strategy to refine and test the usability of an artificial intelligence
(AI)-powered smartphone app, Sugar Smart, to be used by children's
parents/caregivers for the maintenance of dietary records and oral hygiene habits
of children in Maharashtra, India, through a single-arm parallel randomized
control trial. MATERIALS AND METHODS: The app, developed based on validated
research on local dietary habits, was utilized by 100 children. To evaluate the
app, two validated questionnaires and a 7-day diet diary format were employed.
The first questionnaire assessed ease of use and compliance using a Chi-squared
test, while the second examined improvements in dietary awareness before and
after app usage through a paired t-test. RESULTS: The findings indicate that a
significant majority of users positively perceived the mobile app. The first
questionnaire showed high ease of use and positive feedback (87% found it easy to
use, 99% reported it helped regulate sugar intake). The Chi-squared test
confirmed the statistical significance ($p < 0.05$). The second questionnaire
revealed significant improvements in oral health awareness and diet (correct
brushing: 64-98%, reduced sugar usage: 28-91%). The paired t-test confirmed these
improvements ($p < 0.05$), validating the app's effectiveness in enhancing dietary
habits and oral health awareness. CONCLUSION: The study provides strong evidence
for the effectiveness of the digital app in improving parental awareness and
management of children's sugar intake. The significant improvements observed

underscore the app's potential as a valuable tool in pediatric dentistry and public health. CLINICAL SIGNIFICANCE: This app demonstrates the potential to revolutionize dietary monitoring and behavior change in pediatric populations, promoting healthier dietary behaviors and thus being an effective digital tool in the reduction of caries burden. HOW TO CITE THIS ARTICLE: Agrawal PV, Rathi N, Sinnarkar SE, et al. Assessment of Compliance and Validity of Digital Application-based Diet Diary with Suggestive Local Food Habits for Prevention of Dental Caries in Children. Int J Clin Pediatr Dent 2025;18(2):191-197.
CI - Copyright ¬© 2025; The Author(s).
FAU - Agrawal, Priyanka V
AU - Agrawal PV
AUID- ORCID: 0000-0002-7660-0550
AD - Department of Pedodontics and Preventive Dentistry, Dr D Y Patil Dental College and Hospital, Dr D Y Patil Vidyapeeth, Pune, Maharashtra, India.
FAU - Rathi, Nilesh
AU - Rathi N
AUID- ORCID: 0000-0003-0595-5191
AD - Department of Pedodontics and Preventive Dentistry, Dr D Y Patil Dental College and Hospital, Dr D Y Patil Vidyapeeth, Pune, Maharashtra, India.
FAU - Sinnarkar, Surabhi E
AU - Sinnarkar SE
AD - Department of Pedodontics and Preventive Dentistry, Dr D Y Patil Dental College and Hospital, Dr D Y Patil Vidyapeeth, Pune, Maharashtra, India.
FAU - Tasgaonkar, Aditi
AU - Tasgaonkar A
AUID- ORCID: 0009-0001-2522-3871
AD - Department of Pedodontics and Preventive Dentistry, Dr D Y Patil Dental College and Hospital, Dr D Y Patil Vidyapeeth, Pune, Maharashtra, India.
FAU - Kotnis, Ritu
AU - Kotnis R
AD - Department of Pedodontics and Preventive Dentistry, Dr D Y Patil Dental College and Hospital, Dr D Y Patil Vidyapeeth, Pune, Maharashtra, India.
FAU - Joshi, Pawan R
AU - Joshi PR
AD - Department of Pediatric and Preventive Dentistry, VYWS Dental College and Hospital, Amravati, Maharashtra, INDIA.
LA - eng
PT - Journal Article
DEP - 20250320
PL - India

TA - Int J Clin Pediatr Dent
JT - International journal of clinical pediatric dentistry
JID - 101585405
PMC - PMC12099229
OTO - NOTNLM
OT - Dental caries prevention
OT - Diet counseling
OT - Digital dentistry
OT - Pediatric nutrition
OT - Preventive dental care
COIS- Source of support: Nil Conflict of interest: NoneConflict of interest: None
EDAT- 2025/05/26 12:37
MHDA- 2025/05/26 12:38
PMCR- 2025/03/01
CRDT- 2025/05/26 06:15
PHST- 2024/08/27 00:00 [received]
PHST- 2024/10/29 00:00 [accepted]
PHST- 2025/05/26 12:38 [medline]
PHST- 2025/05/26 12:37 [pubmed]
PHST- 2025/05/26 06:15 [entrez]
PHST- 2025/03/01 00:00 [pmc-release]
AID - 10.5005/jp-journals-10005-3047 [doi]
PST - ppublish
SO - Int J Clin Pediatr Dent. 2025 Feb;18(2):191-197. doi: 10.5005/jp-journals-10005-3047. Epub 2025 Mar 20.

PMID- 40251567
OWN - NLM
STAT- MEDLINE
DCOM- 20250419
LR - 20250421
IS - 1472-6831 (Electronic)
IS - 1472-6831 (Linking)
VI - 25
IP - 1
DP - 2025 Apr 18
TI - Artificial intelligence (AI) in restorative dentistry: current trends and future prospects.
PG - 592
LID - 10.1186/s12903-025-05989-1 [doi]
LID - 592
AB - BACKGROUND: Artificial intelligence (AI) holds immense potential in revolutionizing restorative dentistry, offering transformative solutions for diagnostic, prognostic, and treatment planning tasks. Traditional restorative dentistry faces challenges such as clinical variability, resource limitations, and the need for data-driven diagnostic accuracy. AI's ability to address these issues by providing consistent, precise, and data-driven solutions is gaining

significant attention. This comprehensive literature review explores AI applications in caries detection, endodontics, dental restorations, tooth surface loss, tooth shade determination, and regenerative dentistry. While this review focuses on restorative dentistry, AI's transformative impact extends to orthodontics, prosthodontics, implantology, and dental biomaterials, showcasing its versatility across various dental specialties. Emerging trends such as AI-powered robotic systems, virtual assistants, and multi-modal data integration are paving the way for groundbreaking innovations in restorative dentistry.
METHODS: Methodologically, a systematic approach was employed, focusing on English-language studies published between 2020-2025(January), resulting in 63 peer-reviewed publications for analysis. Studies in caries detection, pedodontics, dental restorations, endodontics, tooth surface loss, and tooth shade determination highlighted AI trends and advancements. Inclusion criteria focused on AI applications in restorative dentistry, and publication timeframe.
PRISMA guidelines were followed to ensure transparency in study selection, emphasizing on accuracy metrics and clinical relevance. The study selection process was carefully documented, and a flowchart of the stages, including identification, screening, eligibility, and inclusion, is shown in Fig.¬†1 to provide further clarity and reproducibility in the selection process. RESULTS: The review identified significant advancements in AI-driven solutions across multiple domains of restorative dentistry. Notable studies demonstrated AI's ability to achieve high diagnostic accuracy, such as up to 95% accuracy in caries detection, and its capacity to improve treatment planning efficiency, thus reducing patient chair time. Predictive analytics for personalized treatments was another area where AI has shown substantial promise. CONCLUSION: The review discussed trends, challenges, and future research directions in AI-driven dentistry, highlighting the transformative potential of AI in optimizing dental

care. Key challenges include data privacy concerns, algorithmic bias,
interpretability of AI decision-making processes, and the need for standardized
AI training programs in dental education. Further research should focus on
integrating AI with emerging technologies like 3D printing for personalized
restorations, and developing AI training programs for dental professionals.
CLINICAL SIGNIFICANCE: The integration of AI into restorative dentistry offers
precision-driven solutions for improved patient outcomes. By enabling faster
diagnostics, personalized treatment approaches, and preventive care strategies,
AI can significantly enhance patient-centered care and clinical efficiency. This
review contributes to advancing the understanding and implementation of AI in
dental practice by synthesizing key findings, identifying trends, and addressing
challenges.
CI - ¬© 2025. The Author(s).
FAU - Najeeb, Mariya
AU - Najeeb M
AD - Department of Operative Dentistry and Endodontics, Fatima Jinnah Dental College
Hospital, 100 Feet Road, Azam Town Near DHA Phase 1, Karachi, Pakistan.
FAU - Islam, Shahid
AU - Islam S
AD - Department of Operative Dentistry and Endodontics, Fatima Jinnah Dental College
Hospital, 100 Feet Road, Azam Town Near DHA Phase 1, Karachi, Pakistan.
shahidislam14@gmail.com.
LA - eng
PT - Journal Article
PT - Review
DEP - 20250418
PL - England
TA - BMC Oral Health
JT - BMC oral health
JID - 101088684
SB - IM
MH - *Artificial Intelligence/trends
MH - Humans
MH - *Dental Restoration, Permanent/trends
MH - Dental Caries/diagnosis/therapy
MH - *Dentistry/trends
PMC - PMC12008862
OTO - NOTNLM
OT - Artificial intelligence

OT - Artificial neural networks
OT - Caries detection
OT - Deep learning
OT - Machine learning
OT - Restorative dentistry
COIS- Declarations. Ethics approval and consent to participate: Not applicable. Consent
      for publication: Not applicable. Competing interests: The authors declare no
      competing interests.
EDAT- 2025/04/19 00:56
MHDA- 2025/04/19 00:57
PMCR- 2025/04/18
CRDT- 2025/04/18 23:36
PHST- 2024/12/07 00:00 [received]
PHST- 2025/04/11 00:00 [accepted]
PHST- 2025/04/19 00:57 [medline]
PHST- 2025/04/19 00:56 [pubmed]
PHST- 2025/04/18 23:36 [entrez]
PHST- 2025/04/18 00:00 [pmc-release]
AID - 10.1186/s12903-025-05989-1 [pii]
AID - 5989 [pii]
AID - 10.1186/s12903-025-05989-1 [doi]
PST - epublish
SO - BMC Oral Health. 2025 Apr 18;25(1):592. doi: 10.1186/s12903-025-05989-1.

PMID- 39968964
OWN - NLM
STAT- MEDLINE
DCOM- 20250822
LR - 20250822
IS - 2300-9020 (Electronic)
IS - 1644-387X (Linking)
VI - 62
IP - 4
DP - 2025 Jul-Aug
TI - Knowledge, perception and attitude of dentists regarding the role of artificial
      intelligence in the field of pediatric dentistry: An online questionnaire study.
PG - 645-655
LID - 10.17219/dmp/183901 [doi]
AB - BACKGROUND: Knowledge on the potential applications of artificial intelligence
      (AI) as a diagnostic instrument in the domain of pediatric dentistry is
      imperative, as AI may significantly influence present and future dental practice.
      OBJECTIVES: The present study aimed to evaluate the knowledge, perception and
      attitude of pediatric dentists and postgraduate students in the pediatric

specialty with regard to the employment of AI in pediatric dental practice.
MATERIAL AND METHODS: An online questionnaire survey was conducted among 375 participants (92 postgraduates, 203 faculty members and 80 private practitioners), who were provided with 19 closeended questions through the Google Forms link sent via email. The questions referred to the knowledge, perception and attitude of the participants, with 17 questions answered using a three-point Likert scale and 2 of them being multiple-choice questions. The responses were analyzed using the œá2, Kruskal-Wallis and Mann-Whitney U tests. RESULTS: A total of 62% of the participants were familiar with the term 'artificial intelligence', and the majority confirmed that AI could be used for the identification of plaque (57%) and supernumerary teeth (52%), the detection of early childhood caries (ECC) (68%) and the ectopic eruption of first permanent molars (67%), the assessment of child psychology (82%), and the estimation of chronological age (67%). Most participants felt that AI training should be incorporated into the postgraduate curriculum (82%) and were willing to introduce AI to clinical practice (87%). The barriers related to the use of AI were high costs (83%), the lack of training after graduation (78%), the lack of technical knowledge (77%), the fear of misdiagnosis (73%), and the lack of awareness (71%). CONCLUSIONS: The present study concluded that although most pedodontists and postgraduate students had knowledge on AI, there were many obstacles connected with the use of AI in the field of pediatric dentistry. Therefore, the basic training of AI should be included in the curriculum of postgraduate studies.
FAU - Razdan, Priyanka
AU - Razdan P
AD - Department of Pediatric and Preventive Dentistry, Yogita Dental College and Hospital, Khed, India.
FAU - Das, Anirban
AU - Das A
AD - Department of Dentistry, Jagannath Gupta Institute of Medical Sciences and Hospital, Kolkata, India.
FAU - Habiba, Syeda
AU - Habiba S

AD - Department of Pediatric and Preventive Dentistry, Jal Mahal Dental Hospital,
      Jaipur, India.
FAU - Doley, Sulekha
AU - Doley S
AD - Department of Pediatric and Preventive Dentistry, Regional Dental College and
      Hospital, Guwahati, India.
FAU - Tiwari, Durgesh Ashok
AU - Tiwari DA
AD - Department of Pediatric and Preventive Dentistry, Yogita Dental College and
      Hospital, Khed, India.
FAU - Hazari, Prachi
AU - Hazari P
AD - Department of Pediatric and Preventive Dentistry, Nanded Rural Dental College and
      Research Center, India.
LA - eng
PT - Journal Article
PL - Poland
TA - Dent Med Probl
JT - Dental and medical problems
JID - 101205669
SB - IM
MH - Humans
MH - *Pediatric Dentistry/education
MH - Surveys and Questionnaires
MH - *Artificial Intelligence
MH - Male
MH - Female
MH - *Attitude of Health Personnel
MH - *Health Knowledge, Attitudes, Practice
MH - *Dentists/psychology
MH - Adult
OTO - NOTNLM
OT - artificial intelligence
OT - knowledge
OT - pediatric dentistry
OT - questionnaire
OT - survey
EDAT- 2025/02/19 12:25
MHDA- 2025/09/08 07:49
CRDT- 2025/02/19 07:53
PHST- 2023/12/09 00:00 [received]
PHST- 2024/02/09 00:00 [accepted]
PHST- 2025/09/08 07:49 [medline]
PHST- 2025/02/19 12:25 [pubmed]
PHST- 2025/02/19 07:53 [entrez]
AID - 10.17219/dmp/183901 [doi]
PST - ppublish
SO - Dent Med Probl. 2025 Jul-Aug;62(4):645-655. doi:
10.17219/dmp/183901.

PMID- 39899732
OWN - NLM
STAT- PubMed-not-MEDLINE
LR - 20250212
IS - 2561-6722 (Electronic)
IS - 2561-6722 (Linking)
VI - 8
DP - 2025 Feb 3
TI - Development of Chatbot-Based Oral Health Care for Young Children and Evaluation
of its Effectiveness, Usability, and Acceptability: Mixed Methods Study.
PG - e62738
LID - 10.2196/62738 [doi]
LID - e62738
AB - BACKGROUND: Chatbots are increasingly accepted in public health for their ability
to replicate human-like communication and provide scalable, 24/7 services. The
high prevalence of dental caries in children underscores the need for early and
effective intervention. OBJECTIVE: This study aimed to develop the 30-Day FunDee
chatbot and evaluate its effectiveness, usability, and acceptability in
delivering oral health education to caregivers of children aged 6 to 36 months.
METHODS: The chatbot was created using the artificial intelligence (AI) chatbot
behavior change model, integrating behavioral change theories into content
designed for 3-5 minutes of daily use over 30 days. A pre-post experimental study
was conducted from December 2021 to February 2022 in Hat Yai District, Songkhla
Province, and Maelan District, Pattani Province, Thailand. Fifty-eight caregivers
completed a web-based structured questionnaire at baseline and 2 months post
baseline to evaluate knowledge, protection motivation theory-based perceptions,
and tooth-brushing practices. Usability was assessed via chatbot logfiles and a
web-based questionnaire at 2 months post baseline. Acceptability was evaluated
through three methods: (1) open-ended chatbot interactions on day 30, (2) a
web-based structured questionnaire at 2 months post baseline, and (3)
semistructured telephone interviews with 15 participants 2 weeks post
intervention. Participants for interviews were stratified by adherence levels and

randomly selected from Hatyai and Maelan districts. All self-reported variables
were measured on a 5-point Likert scale (1=lowest, 5=highest). RESULTS: The
chatbot was successfully developed based on the 4 components of the AI chatbot
behavior change model. Participants had a mean age of 34.5 (SD 8.6) years. The
frequency of tooth brushing among caregivers significantly improved, increasing
from 72.4% at baseline to 93.1% two months post baseline (P=.006). Protection
motivation theory-based perceptions also showed significant improvement, with
mean scores rising from 4.0 (SD 0.6) at baseline to 4.5 (SD 0.6) two months post
baseline (P<.001). The chatbot received high ratings for satisfaction (4.7/5, SD
0.6) and usability (4.7/5, SD 0.5). Participants engaged with the chatbot for an
average of 24.7 (SD 7.2) days out of 30. Caregivers praised the chatbot's content
quality, empathetic communication, and multimedia design, but noted the
intervention's lengthy duration and messaging system as limitations. CONCLUSIONS:
The 30-Day FunDee chatbot effectively enhanced caregivers' perceptions of oral
health care and improved tooth-brushing practices for children aged 6-36 months.
High user satisfaction and engagement demonstrate its potential as an innovative
tool for oral health education. These findings warrant further validation through
large-scale, randomized controlled trials.
CI - ¬© Kittiwara Pupong, Jaranya Hunsrisakhun, Samerchit Pithpornchaiyakul, Supawadee
Naorungroj. Originally published in JMIR Pediatrics and Parenting
(https://pediatrics.jmir.org).
FAU - Pupong, Kittiwara
AU - Pupong K
AUID- ORCID: 0000-0001-5046-3933
AD - Dental Public Health Division, Maelan Hospital, Pattani, Thailand.
FAU - Hunsrisakhun, Jaranya
AU - Hunsrisakhun J
AUID- ORCID: 0000-0002-0427-7213
AD - Department of Preventive Dentistry, Faculty of Dentistry, Prince of Songkla
University, 15 Kanjanavanich Rd, Hatyai, Songkhla, 90112, Thailand, 66 74429875,
66 74429875.
AD - Improvement of Oral Health Care Research Unit, Faculty of Dentistry, Prince of
Songkla University, Hatyai, Songkhla, Thailand.

FAU - Pithpornchaiyakul, Samerchit
AU - Pithpornchaiyakul S
AUID- ORCID: 0000-0002-6697-4751
AD - Department of Preventive Dentistry, Faculty of Dentistry, Prince of Songkla
      University, 15 Kanjanavanich Rd, Hatyai, Songkhla, 90112, Thailand, 66 74429875,
      66 74429875.
AD - Improvement of Oral Health Care Research Unit, Faculty of Dentistry, Prince of
      Songkla University, Hatyai, Songkhla, Thailand.
FAU - Naorungroj, Supawadee
AU - Naorungroj S
AUID- ORCID: 0000-0002-2840-327X
AD - Department of Conservative Dentistry, Faculty of Dentistry, Prince of Songkla
      University, Hatyai, Songkhla, Thailand.
LA - eng
PT - Journal Article
DEP - 20250203
PL - Canada
TA - JMIR Pediatr Parent
JT - JMIR pediatrics and parenting
JID - 101727244
PMC - PMC11809939
OTO - NOTNLM
OT - chatbot
OT - children
OT - conversational agents
OT - covid-19
OT - development.
OT - hands-on
OT - in-person toothbrushing
OT - oral health behavior
OT - oral health education
OT - tele-dentistry
COIS- Conflicts of Interest: None declared.
EDAT- 2025/02/03 18:20
MHDA- 2025/02/03 18:21
PMCR- 2025/02/03
CRDT- 2025/02/03 15:23
PHST- 2024/05/30 00:00 [received]
PHST- 2024/11/24 00:00 [revised]
PHST- 2024/11/26 00:00 [accepted]
PHST- 2025/02/03 18:21 [medline]
PHST- 2025/02/03 18:20 [pubmed]
PHST- 2025/02/03 15:23 [entrez]
PHST- 2025/02/03 00:00 [pmc-release]
AID - v8i1e62738 [pii]
AID - 62738 [pii]
AID - 10.2196/62738 [doi]
PST - epublish
SO - JMIR Pediatr Parent. 2025 Feb 3;8:e62738. doi: 10.2196/62738.

PMID- 39781392
OWN - NLM
STAT- PubMed-not-MEDLINE
LR - 20250130
IS - 0974-7052 (Print)
IS - 0975-1904 (Electronic)
IS - 0974-7052 (Linking)
VI - 17
IP - 11
DP - 2024 Nov
TI - Tech Bytes-Harnessing Artificial Intelligence for Pediatric Oral Health: A
      Scoping Review.
PG - 1289-1295
LID - 10.5005/jp-journals-10005-2971 [doi]
AB - AIM AND BACKGROUND: The applications of artificial intelligence (AI) are
      escalating in all frontiers, specifically healthcare. It constitutes the umbrella
      term for a number of technologies that enable machines to independently solve
      problems they have not been programmed to address. With its aid, patient
      management, diagnostics, treatment planning, and interventions can be
      significantly improved. The aim of this review is to analyze the current data to
      assess the applications of artificial intelligence in pediatric dentistry and
      determine their clinical effectiveness. MATERIALS AND METHODS: A search of
      published studies in PubMed, Web of Science, Scopus, and Google Scholar databases
      was included till January 2024. RESULTS: This review consisted of 30 published
      studies in the English language. The use of AI has been employed in the detection
      of dental caries, dental plaque, behavioral science, interceptive orthodontics,
      predicting the dental age, and identification of teeth which can enhance patient
      care. CONCLUSION: Artificial intelligence models can be used as an aid to the
      clinician as they are of significant help at individual and community levels in
      identifying an increased risk to dental diseases. CLINICAL SIGNIFICANCE:
      Artificial intelligence can be used as an asset in preventive school health
      programs, dental education for students and parents, and to assist the clinician
      in the dental practice. Further advancements in technology will give rise to

newer potential innovations and applications. HOW TO CITE THIS ARTICLE: Tanna DA, Bhandary S, Hegde SK. Tech Bytes-Harnessing Artificial Intelligence for Pediatric Oral Health: A Scoping Review. Int J Clin Pediatr Dent 2024;17(11):1289-1295.
CI - Copyright ¬© 2024; The Author(s).
FAU - Tanna, Dhvani A
AU - Tanna DA
AUID- ORCID: 0009-0008-4999-5818
AD - Department of Pediatric and Preventive Dentistry, AB Shetty Memorial Institute of Dental Sciences (ABSMIDS), NITTE (Deemed to be University), Mangaluru, Karnataka, India.
FAU - Bhandary, Srikala
AU - Bhandary S
AUID- ORCID: 0000-0001-7682-0345
AD - Department of Pediatric and Preventive Dentistry, AB Shetty Memorial Institute of Dental Sciences (ABSMIDS), NITTE (Deemed to be University), Mangaluru, Karnataka, India.
FAU - Hegde, K Sundeep
AU - Hegde KS
AUID- ORCID: 0000-0003-0385-7539
AD - Department of Pediatric and Preventive Dentistry, Yenepoya Dental College, Mangaluru, Karnataka, India.
LA - eng
PT - Journal Article
PT - Scoping Review
DEP - 20241219
PL - India
TA - Int J Clin Pediatr Dent
JT - International journal of clinical pediatric dentistry
JID - 101585405
PMC - PMC11703760
OTO - NOTNLM
OT - Artificial intelligence
OT - Deep learning
OT - Machine learning
OT - Patient satisfaction
COIS- Source of support: Nil Conflict of interest: NoneConflict of interest: None
EDAT- 2025/01/09 06:22
MHDA- 2025/01/09 06:23
PMCR- 2024/12/01
CRDT- 2025/01/09 04:19
PHST- 2025/01/09 06:23 [medline]
PHST- 2025/01/09 06:22 [pubmed]
PHST- 2025/01/09 04:19 [entrez]
PHST- 2024/12/01 00:00 [pmc-release]
AID - 10.5005/jp-journals-10005-2971 [doi]

PST - ppublish
SO - Int J Clin Pediatr Dent. 2024 Nov;17(11):1289-1295. doi:
      10.5005/jp-journals-10005-2971. Epub 2024 Dec 19.

PMID- 39777218
OWN - NLM
STAT- PubMed-not-MEDLINE
LR - 20250108
IS - 2673-4842 (Electronic)
IS - 2673-4842 (Linking)
VI - 5
DP - 2024
TI - An overview of artificial intelligence based automated diagnosis in paediatric
      dentistry.
PG - 1482334
LID - 10.3389/froh.2024.1482334 [doi]
LID - 1482334
AB - Artificial intelligence (AI) is a subfield of computer science with the goal of
      creating intelligent machines (1) Machine learning is a branch of artificial
      intelligence. In machine learning a datasets are used for training diagnostic
      algorithms. This review comprehensively explains the applications of AI in the
      diagnosis in paediatric dentistry. The online database searches were performed
      between 25th May 2024 to 1st July 2024. Original research studies that focus on
      the automated diagnosis or predicted the outcome in Paediatric dentistry using AI
      were included in this review. AI is being used in varied domains of paediatric
      dentistry like diagnosis of supernumerary and submerged teeth, early diagnosis of
      dental caries, diagnosis of dental plaques, assessment of bone age, forensic
      dentistry and preventive oral dental healthcare kit. The field of AI, deep
      machine learning and CNN's is an upcoming and newer area, with new developments
      this will open up areas for more sophisticated algorithms in multiple layers to
      predict accurately, when compared to experienced Paediatric dentists.
CI - ¬© 2024 Rajinikanth, Rajkumar, Rajinikanth, Anandhapandian and J.
FAU - Rajinikanth, Suba B
AU - Rajinikanth SB
AD - Faculty of Medicine, Srilalithambigai Medical College and Hospital, DR MGR
      Educational and Research Institute, Chennai, India.
FAU - Rajkumar, Densingh Samuel Raj
AU - Rajkumar DSR

AD - Department of Traumotology & Orthopedics, Kursk State Medical University, Kursk,
      Russia.
FAU - Rajinikanth, Akshay
AU - Rajinikanth A
AD - School of Computer Science and Engineering, VIT University, Vellore, India.
FAU - Anandhapandian, Ponsekar Abraham
AU - Anandhapandian PA
AD - Department of Prosthodontics, Thai Moogambigai Dental College and Hospital, Dr.
      M.G.R. Educational and Research Institute, Chennai, India.
FAU - J, Bhuvaneswarri
AU - J B
AD - Department of Periodontics, Sri Balaji Dental College and Hospital, BIHER,
      Chennai, India.
LA - eng
PT - Journal Article
PT - Review
DEP - 20241224
PL - Switzerland
TA - Front Oral Health
JT - Frontiers in oral health
JID - 9918227262706676
PMC - PMC11703950
OTO - NOTNLM
OT - artificial intelligence
OT - automated diagnosis
OT - convoluted neural network
OT - deep learning
OT - dental caries
OT - dental plaques
OT - paediatric dentistry
COIS- The authors declare that the research was conducted in the absence of any
      commercial or financial relationships that could be construed as a potential
      conflict of interest.
EDAT- 2025/01/09 00:23
MHDA- 2025/01/09 00:24
PMCR- 2024/12/24
CRDT- 2025/01/08 18:03
PHST- 2024/08/18 00:00 [received]
PHST- 2024/11/29 00:00 [accepted]
PHST- 2025/01/09 00:24 [medline]
PHST- 2025/01/09 00:23 [pubmed]
PHST- 2025/01/08 18:03 [entrez]
PHST- 2024/12/24 00:00 [pmc-release]
AID - 10.3389/froh.2024.1482334 [doi]
PST - epublish
SO - Front Oral Health. 2024 Dec 24;5:1482334. doi: 10.3389/froh.2024.1482334.
      eCollection 2024.

PMID- 39768085
OWN - NLM
STAT- PubMed-not-MEDLINE
LR - 20250108
IS - 2306-5354 (Print)
IS - 2306-5354 (Electronic)
IS - 2306-5354 (Linking)
VI - 11
IP - 12
DP - 2024 Dec 13
TI - Artificial Intelligence in Dentistry: A Descriptive Review.
LID - 10.3390/bioengineering11121267 [doi]
LID - 1267
AB - Artificial intelligence (AI) is an area of computer science that focuses on
      designing machines or systems that can perform operations that would typically
      need human intelligence. AI is a rapidly developing technology that has grabbed
      the interest of researchers from all across the globe in the healthcare industry.
      Advancements in machine learning and data analysis have revolutionized oral
      health diagnosis, treatment, and management, making it a transformative force in
      healthcare, particularly in dentistry. Particularly in dentistry, AI is becoming
      increasingly prevalent as it contributes to the diagnosis of oro-facial diseases,
      offers treatment modalities, and manages practice in the dental operatory. All
      dental disciplines, including oral medicine, operative dentistry, pediatric
      dentistry, periodontology, orthodontics, oral and maxillofacial surgery,
      prosthodontics, and forensic odontology, have adopted AI. The majority of AI
      applications in dentistry are for diagnoses based on radiographic or optical
      images, while other tasks are less applicable due to constraints such as data
      availability, uniformity, and computational power. Evidence-based dentistry is
      considered the gold standard for decision making by dental professionals, while
      AI machine learning models learn from human expertise. Dentistry AI and
      technology systems can provide numerous benefits, such as improved diagnosis
      accuracy and increased administrative task efficiency. Dental practices are
      already implementing various AI applications, such as imaging and diagnosis,

treatment planning, robotics and automation, augmented and virtual reality, data
analysis and predictive analytics, and administrative support. The dentistry
field has extensively used artificial intelligence to assist less-skilled
practitioners in reaching a more precise diagnosis. These AI models effectively
recognize and classify patients with various oro-facial problems into different
risk categories, both individually and on a group basis. The objective of this
descriptive review is to review the most recent developments of AI in the field
of dentistry.
FAU - Mallineni, Sreekanth Kumar
AU - Mallineni SK
AUID- ORCID: 0000-0002-9432-2590
AD - Pediatric Dentistry, Dr. Sulaiman Alhabib Medical Group, Rayyan, Riyadh 14212,
Saudi Arabia.
AD - Division for Globalization Initiative, Liaison Center for Innovative Dentistry,
Graduate School of Dentistry, Tohoku University, Sendai 980-8575, Japan.
FAU - Sethi, Mallika
AU - Sethi M
AD - Department of Periodontics, Inderprastha Dental College and Hospital, Ghaziabad
201010, Uttar Pradesh, India.
FAU - Punugoti, Dedeepya
AU - Punugoti D
AD - Pediatric Dentistry, Sri Vydya Dental Hospital, Ongole 52300, Andhra Pradesh,
India.
FAU - Kotha, Sunil Babu
AU - Kotha SB
AUID- ORCID: 0000-0001-5561-6206
AD - Preventive Dentistry Department, Pediatric Dentistry Division, College of
Dentistry, Riyadh Elm University, Riyadh 13244, Saudi Arabia.
AD - Department of Pediatric and Preventive Dentistry, Datta Meghe Institute of
Medical Sciences, Wardha 442004, Maharashtra, India.
FAU - Alkhayal, Zikra
AU - Alkhayal Z
AD - Therapeutics & Biomarker Discovery for Clinical Applications, Cell Therapy &
Immunobiology Department, King Faisal Specialist Hospital & Research Centre, P.O.
Box 3354, Riyadh 11211, Saudi Arabia.
AD - Department of Dentistry, King Faisal Specialist Hospital & Research Centre, P.O.
Box 3354, Riyadh 11211, Saudi Arabia.

FAU - Mubaraki, Sarah
AU - Mubaraki S
AUID- ORCID: 0000-0003-3044-6794
AD - Preventive Dentistry Department, Pediatric Dentistry Division, College of
      Dentistry, Riyadh Elm University, Riyadh 13244, Saudi Arabia.
FAU - Almotawah, Fatmah Nasser
AU - Almotawah FN
AD - Preventive Dentistry Department, Pediatric Dentistry Division, College of
      Dentistry, Riyadh Elm University, Riyadh 13244, Saudi Arabia.
FAU - Kotha, Sree Lalita
AU - Kotha SL
AUID- ORCID: 0000-0001-5764-8308
AD - Department of Basic Dental Sciences, College of Dentistry, Princess Nourah bint
      Abdulrahman University, P.O. Box 84428, Riyadh 11671, Saudi Arabia.
FAU - Sajja, Rishitha
AU - Sajja R
AUID- ORCID: 0000-0003-1501-5726
AD - Clinical Data Management, Global Data Management and Centralized Monitoring,
      Global Development Operations, Bristol Myers Squibb, Pennington, NJ 07922, USA.
FAU - Nettam, Venkatesh
AU - Nettam V
AD - Department of Orthodontics, Narayana Dental College and Hospital, Nellore 523004,
      Andhra Pradesh, India.
FAU - Thakare, Amar Ashok
AU - Thakare AA
AD - Department of Restorative Dentistry and Prosthodontics, College of Dentistry,
      Majmaah University, Al-Zulfi 11952, Saudi Arabia.
FAU - Sakhamuri, Srinivasulu
AU - Sakhamuri S
AUID- ORCID: 0000-0001-8615-3312
AD - Department of Conservative Dentistry & Endodontics, Narayana Dental College and
      Hospital, Nellore 523004, Andhra Pradesh, India.
LA - eng
PT - Journal Article
PT - Review
DEP - 20241213
PL - Switzerland
TA - Bioengineering (Basel)
JT - Bioengineering (Basel, Switzerland)
JID - 101676056
PMC - PMC11673909
OTO - NOTNLM
OT - artificial intelligence
OT - dental caries
OT - dentistry
OT - oral health

COIS- The authors declare no conflicts of interest.
EDAT- 2025/01/08 06:21
MHDA- 2025/01/08 06:22
PMCR- 2024/12/13
CRDT- 2025/01/08 01:16
PHST- 2024/11/15 00:00 [received]
PHST- 2024/12/09 00:00 [revised]
PHST- 2024/12/11 00:00 [accepted]
PHST- 2025/01/08 06:22 [medline]
PHST- 2025/01/08 06:21 [pubmed]
PHST- 2025/01/08 01:16 [entrez]
PHST- 2024/12/13 00:00 [pmc-release]
AID - bioengineering11121267 [pii]
AID - bioengineering-11-01267 [pii]
AID - 10.3390/bioengineering11121267 [doi]
PST - epublish
SO - Bioengineering (Basel). 2024 Dec 13;11(12):1267. doi:
      10.3390/bioengineering11121267.

PMID- 39650341
OWN - NLM
STAT- PubMed-not-MEDLINE
LR - 20241211
IS - 2376-5992 (Electronic)
IS - 2376-5992 (Linking)
VI - 10
DP - 2024
TI - Comparative analysis of deep learning algorithms for dental caries detection and
      prediction from radiographic images: a comprehensive umbrella review.
PG - e2371
LID - 10.7717/peerj-cs.2371 [doi]
LID - e2371
AB - BACKGROUND: In recent years, artificial intelligence (AI) and deep learning (DL)
      have made a considerable impact in dentistry, specifically in advancing image
      processing algorithms for detecting caries from radiographical images. Despite
      this progress, there is still a lack of data on the effectiveness of these
      algorithms in accurately identifying caries. This study provides an overview
      aimed at evaluating and comparing reviews that focus on the detection of dental
      caries (DC) using DL algorithms from 2D radiographs. MATERIALS AND METHODS: This
      comprehensive umbrella review adhered to the "Reporting guideline for overviews
      of reviews of healthcare interventions" (PRIOR). Specific keywords were generated
      to assess the accuracy of AI and DL algorithms in detecting DC from

radiographical images. To ensure the highest quality of research, thorough
searches were performed on PubMed/Medline, Web of Science, Scopus, and Embase.
Additionally, bias in the selected articles was rigorously assessed using the
Joanna Briggs Institute (JBI) tool. RESULTS: In this umbrella review, seven
systematic reviews (SRs) were assessed from a total of 77 studies included.
Various DL algorithms were used across these studies, with conventional neural
networks and other techniques being the predominant methods for detecting DC. The
SRs included in the study examined 24 original articles that used 2D
radiographical images for caries detection. Accuracy rates varied between 0.733
and 0.986 across datasets ranging in size from 15 to 2,500 images. CONCLUSION:
The advancement of DL algorithms in detecting and predicting DC through
radiographic imaging is a significant breakthrough. These algorithms excel in
extracting subtle features from radiographic images and applying machine learning
techniques to achieve highly accurate predictions, often outperforming human
experts. This advancement holds immense potential to transform diagnostic
processes in dentistry, promising to considerably improve patient outcomes.
CI - ¬© 2024 Dashti et al.
FAU - Dashti, Mahmood
AU - Dashti M
AD - Dentofacial Deformities Research Center, Research Institute of Dental Sciences,
Shahid Beheshti University of Medical Sciences, Tehran, Iran.
FAU - Londono, Jimmy
AU - Londono J
AD - Department of Prosthodontics, Dental College of Georgia at Augusta University,
Augusta, Georgia, United States.
FAU - Ghasemi, Shohreh
AU - Ghasemi S
AD - Department of Oral and Maxillofacial Surgery, Queen Mary College of Medicine and
Dentistry, London, United Kingdom.
FAU - Zare, Niusha
AU - Zare N
AD - Department of Oral and Maxillofacial Radiology, Islamic Azad University Tehran
Dental Branch, Tehran, Iran.
FAU - Samman, Meyassara

AU - Samman M
AUID- ORCID: 0000-0003-2256-8601
AD - Department of Dental Public Health, College of Dentistry, King Abdulaziz
      University, Jeddah, Saudi Arabia.
FAU - Ashi, Heba
AU - Ashi H
AD - Department of Dental Public Health, College of Dentistry, King Abdulaziz
      University, Jeddah, Saudi Arabia.
FAU - Amirzade-Iranaq, Mohammad Hosein
AU - Amirzade-Iranaq MH
AD - Faculty of Dentistry, Universal Scientific Education and Research Network
      (USERN), Tehran University of Medical Sciences, Tehran, Iran.
FAU - Khosraviani, Farshad
AU - Khosraviani F
AD - UCLA School of Dentistry, Los Angeles, CA, United States.
FAU - Sabeti, Mohammad
AU - Sabeti M
AUID- ORCID: 0000-0003-0252-9584
AD - Department of Preventive and Restorative Dental Sciences, San Francisco School of
      Dentistry, San Francisco, CA, United States.
FAU - Khurshid, Zohaib
AU - Khurshid Z
AUID- ORCID: 0000-0001-7998-7335
AD - Department of Prosthodontics and Dental Implantology, King Faisal University, Al
      Hofuf, Saudi Arabia.
LA - eng
PT - Journal Article
PT - Review
DEP - 20241112
PL - United States
TA - PeerJ Comput Sci
JT - PeerJ. Computer science
JID - 101660598
PMC - PMC11622875
OTO - NOTNLM
OT - Artificial intelligence
OT - Caries detection
OT - Deep learning
OT - Dental caries
OT - Dental radiograph
OT - Diagnosis
OT - Prediction
COIS- The authors declare that they have no competing interests.
EDAT- 2024/12/09 17:33
MHDA- 2024/12/09 17:34
PMCR- 2024/11/12
CRDT- 2024/12/09 06:14
PHST- 2024/02/05 00:00 [received]
PHST- 2024/09/09 00:00 [accepted]

PHST- 2024/12/09 17:34 [medline]
PHST- 2024/12/09 17:33 [pubmed]
PHST- 2024/12/09 06:14 [entrez]
PHST- 2024/11/12 00:00 [pmc-release]
AID - cs-2371 [pii]
AID - 10.7717/peerj-cs.2371 [doi]
PST - epublish
SO - PeerJ Comput Sci. 2024 Nov 12;10:e2371. doi: 10.7717/peerj-cs.2371. eCollection 2024.

PMID- 39565341
OWN - NLM
STAT- Publisher
LR - 20241120
IS - 2724-6337 (Electronic)
IS - 2724-6329 (Linking)
DP - 2024 Nov 20
TI - AI-based prediction and classification of root caries using radiographic images.
LID - 10.23736/S2724-6329.24.04967-2 [doi]
AB - BACKGROUND: Root surface caries, commonly known as root decay, is a common dental disorder that affects tooth roots. Like enamel-based tooth decay, root caries attack exposed root surfaces caused by gum recession or periodontal disease. Older persons with gum recession, tooth loss, or poor oral hygiene may be more likely to develop this disorder. Dental root caries must be diagnosed early to improve treatment and prevention. This research will examine radiographic image-based AI-based root caries prediction algorithms. METHODS: Saveetha Dental College supplied 200 root surface radiographs. An expert dentist and dental radiologist confirmed one hundred teeth with root caries and 100 without. Edited and segmented radiographic images. Orange, a machine learning squeeze net embedding model with Naive Bayes, Logistic Regression, and neural networks, was used to assess prediction accuracy. Training and test data were split 80/20. Cross-validation, confusion matrix, and ROC analysis assessed model performance. This study examined precision and recall. RESULTS: Na√Øve bayes and logistic regression have 96% and 100% accuracy, but class accuracy is -94% and 100% in image classification of root caries was seen. CONCLUSIONS: AI-based root caries

prediction utilizing radiographic images would improve dental care by diagnosing and treating early, accurately, and personalized. With appropriate deployment, research, and ethics, AI integration in dentistry could benefit practitioners and patients. Dental professionals and AI experts must work together to maximize this new technology.AI integration in dentistry can significantly improve root caries diagnosis and treatment by predicting root caries using radiographic images. This early detection reduces treatment need and time. Collaboration between dental professionals and AI experts is crucial for maximizing benefits.

FAU - Yadalam, Pradeep K
AU - Yadalam PK
AD - Department of Periodontics, Saveetha Dental College and Hospitals, Saveetha Institute of Medical and Technical Sciences, Saveetha University, Chennai, India - pradeepkumar.sdc@saveetha.com.
FAU - Manickavasagam, Jeevitha
AU - Manickavasagam J
AD - Department of Periodontics, Saveetha Dental College and Hospitals, Saveetha Institute of Medical and Technical Sciences, Saveetha University, Chennai, India.
FAU - Sasikumar, Trisha
AU - Sasikumar T
AD - Saveetha Dental College and Hospitals, Saveetha Institute of Medical and Technological Science (SIMATS), Saveetha University, Chennai, India.
FAU - Marrapodi, Maria M
AU - Marrapodi MM
AD - Department of Woman, Child and General and Specialist Surgery, Luigi Vanvitelli University of Campania, Naples, Italy.
FAU - Ronsivalle, Vincenzo
AU - Ronsivalle V
AD - Department of Biomedical and Surgical and Biomedical Sciences, Catania University, Catania, Italy.
FAU - Cicci√π, Marco
AU - Cicci√π M
AD - Department of Biomedical and Surgical and Biomedical Sciences, Catania University, Catania, Italy.
FAU - Minervini, Giuseppe
AU - Minervini G
AD - Saveetha Dental College and Hospitals, Saveetha Institute of Medical and

Technological Science (SIMATS), Saveetha University, Chennai, India.
AD - Multidisciplinary Department of Medical-Surgical and Odontostomatological
      Specialties, Luigi Vanvitelli University of Campania, Naples, Italy.
LA - eng
PT - Journal Article
DEP - 20241120
PL - Italy
TA - Minerva Dent Oral Sci
JT - Minerva dental and oral science
JID - 101778009
SB - IM
EDAT- 2024/11/20 12:24
MHDA- 2024/11/20 12:24
CRDT- 2024/11/20 10:48
PHST- 2024/11/20 12:24 [medline]
PHST- 2024/11/20 12:24 [pubmed]
PHST- 2024/11/20 10:48 [entrez]
AID - S2724-6329.24.04967-2 [pii]
AID - 10.23736/S2724-6329.24.04967-2 [doi]
PST - aheadofprint
SO - Minerva Dent Oral Sci. 2024 Nov 20. doi: 10.23736/S2724-6329.24.04967-2.

PMID- 39436248
OWN - NLM
STAT- MEDLINE
DCOM- 20241022
LR - 20241107
IS - 0039-1735 (Print)
IS - 0039-1735 (Linking)
VI - 103
IP - 5
DP - 2024
TI - [The use of artificial intelligence in modern dentistry in the Russian
      Federation].
PG - 42-45
LID - 10.17116/stomat202410305142 [doi]
AB - OBJECTIVE: The aim of the study is to identify the prospects and possibilities of
      using artificial intelligence (AI) and domestic software in the provision of
      organization dental care in the Russian Federation. MATERIALS AND METHODS: An
      analysis of the actual situation on the use of domestic computer programs using
      AI for the provision of medical care in the Russian Federation was carried out
      based on information presented on the official websites of software developers,

in scientific sources of information and analytical systems of the Higher Attestation Commission, eLibrary, PubMed, Scopus, Google Scholar. Content analysis and analytical method were used, followed by interpretation of the data and conclusions obtained. RESULTS: To date, information technologies and domestic software aimed at automation, improving safety and quality of medical care are being actively introduced. The requirements for AI-based programs are justified, namely: safety, accessibility for medical professionals and patients, as well as high competitiveness among developers. AI helps to process large amounts of medical data, contributes to the creation of a personalized approach to patient treatment, automates and optimizes administrative processes in healthcare, increases the accuracy of diagnosis, identification of early signs of the disease, predicts the outcome of treatment, neural networks accelerate the development of remote healthcare and telemedicine. However, it is the doctor who should be responsible for the decisions made based on the "tips" and recommendations of artificial intelligence. CONCLUSION: The use of computer programs using artificial intelligence in dentistry opens up significant prospects for improving the quality of medical care, interaction with patients and improving the level of education of doctors. The most popular programs will be in therapeutic and pediatric dentistry, aimed, among other things, at the prevention of dental diseases.
FAU - Losev, F F
AU - Losev FF
AUID- ORCID: 0000-0002-9448-9614
AD - National Medical Research Center of Dental and Maxillofacial Surgery, Moscow, Russia.
FAU - Sorokina, A A
AU - Sorokina AA
AUID- ORCID: 0000-0003-0411-9837
AD - Central State Medical Academy of Presidential Affairs, Moscow, Russia.
FAU - Salakhov, A K
AU - Salakhov AK
AUID- ORCID: 0000-0002-0791-1363
AD - Kazan State Medical University, Kazan, Russia.

FAU - Dokin, S P
AU - Dokin SP
AUID- ORCID: 0009-0007-9871-5280
AD - Central State Medical Academy of Presidential Affairs, Moscow, Russia.
LA - rus
PT - English Abstract
PT - Journal Article
PT - Review
TT - Ispol'zovanie iskusstvennogo intellekta v sovremennoi stomatologii v Rossiiskoi
      Federatsii.
PL - Russia (Federation)
TA - Stomatologiia (Mosk)
JT - Stomatologiia
JID - 0412072
SB - IM
MH - Russia
MH - *Artificial Intelligence
MH - Humans
MH - *Dentistry
MH - Software
MH - Telemedicine
MH - Dental Care
OTO - NOTNLM
OT - artificial intelligence
OT - caries prediction
OT - dental care
OT - diagnostics
OT - neural networks
EDAT- 2024/10/22 16:24
MHDA- 2024/10/22 16:25
CRDT- 2024/10/22 09:44
PHST- 2024/10/22 16:25 [medline]
PHST- 2024/10/22 16:24 [pubmed]
PHST- 2024/10/22 09:44 [entrez]
AID - 10.17116/stomat202410305142 [doi]
PST - ppublish
SO - Stomatologiia (Mosk). 2024;103(5):42-45. doi: 10.17116/stomat202410305142.

PMID- 39396775
OWN - NLM
STAT- MEDLINE
DCOM- 20241123
LR - 20241125
IS - 1879-176X (Electronic)
IS - 0300-5712 (Linking)
VI - 151
DP - 2024 Dec
TI - Diagnostic accuracy of artificial intelligence for approximal caries on bitewing
      radiographs: A systematic review and meta-analysis.
PG - 105388

LID - S0300-5712(24)00558-X [pii]
LID - 10.1016/j.jdent.2024.105388 [doi]
AB - OBJECTIVES: This systematic review and meta-analysis aimed to investigate the
      diagnostic accuracy of Artificial Intelligence (AI) for approximal carious
      lesions on bitewing radiographs. METHODS: This study included randomized
      controlled trials (RCTs) and non-randomized controlled trials (non-RCTs)
      reporting on the diagnostic accuracy of AI for approximal carious lesions on
      bitewing radiographs. The risk of bias was assessed using the Quality Assessment
      of Diagnostic Accuracy Studies (QUADAS-2) tool. A systematic search was conducted
      on November 4, 2023, in PubMed, Cochrane, and Embase databases and an updated
      search was performed on August 28, 2024. The primary outcomes assessed were
      sensitivity, specificity, and overall accuracy. Sensitivity and specificity were
      pooled using a bivariate model. RESULTS: Of the 2,442 studies identified, 21 met
      the inclusion criteria. The pooled sensitivity and specificity of AI were 0.94
      (confidence interval (CI): ¬± 0.78-0.99) and 0.91 (CI: ¬± 0.84-0.95), respectively.
      The positive predictive value (PPV) ranged from 0.15 to 0.87, indicating a
      moderate capacity for identifying true positives among decayed teeth. The
      negative predictive value (NPV) ranged from 0.79 to 1.00, demonstrating a high
      ability to exclude healthy teeth. The diagnostic odds ratio was high, indicating
      strong overall diagnostic performance. CONCLUSIONS: AI models demonstrate
      clinically acceptable diagnostic accuracy for approximal caries on bitewing
      radiographs. Although AI can be valuable for preliminary screening, positive
      findings should be verified by dental experts to prevent unnecessary treatments
      and ensure timely diagnosis. AI models are highly reliable in excluding healthy
      approximal surfaces. CLINICAL SIGNIFICANCE: AI can assist dentists in detecting
      approximal caries on bitewing radiographs. However, expert supervision is
      required to prevent iatrogenic damage and ensure timely diagnosis.
CI - Copyright ¬© 2024 The Author(s). Published by Elsevier Ltd.. All rights reserved.
FAU - Carvalho, Bruna Katherine Guimar√£es

AU - Carvalho BKG
AD - Centre for Translational Medicine, Semmelweis University, T≈±zolt√≥ utca 37-47
      1072, Budapest, Hungary.
FAU - Nolden, Elias-Leon
AU - Nolden EL
AD - Centre for Translational Medicine, Semmelweis University, T≈±zolt√≥ utca 37-47
      1072, Budapest, Hungary.
FAU - Wenning, Alexander Schulze
AU - Wenning AS
AD - Centre for Translational Medicine, Semmelweis University, T≈±zolt√≥ utca 37-47
      1072, Budapest, Hungary.
FAU - Kiss-Dala, Szilvia
AU - Kiss-Dala S
AD - Centre for Translational Medicine, Semmelweis University, T≈±zolt√≥ utca 37-47
      1072, Budapest, Hungary.
FAU - Ag√≥cs, Gergely
AU - Ag√≥cs G
AD - Centre for Translational Medicine, Semmelweis University, T≈±zolt√≥ utca 37-47
      1072, Budapest, Hungary; Department of Biophysics and Radiation Biology,
      Semmelweis University, T≈±zolt√≥ utca 37-47, 1072, Budapest, Hungary.
FAU - R√≥th, Ivett
AU - R√≥th I
AD - Centre for Translational Medicine, Semmelweis University, T≈±zolt√≥ utca 37-47
      1072, Budapest, Hungary; Department of Prosthodontics, Semmelweis University,
      Szentkir√°lyi utca 47 1088, Budapest, Hungary.
FAU - Ker√©mi, Be√°ta
AU - Ker√©mi B
AD - Centre for Translational Medicine, Semmelweis University, T≈±zolt√≥ utca 37-47
      1072, Budapest, Hungary; Department of Restorative Dentistry and Endodontics,
      Semmelweis University, Szentkir√°lyi utca 47, 1088, Budapest, Hungary.
FAU - G√©czi, Zolt√°n
AU - G√©czi Z
AD - Centre for Translational Medicine, Semmelweis University, T≈±zolt√≥ utca 37-47
      1072, Budapest, Hungary; Department of Prosthodontics, Semmelweis University,
      Szentkir√°lyi utca 47 1088, Budapest, Hungary.
FAU - Hegyi, P√©ter
AU - Hegyi P
AD - Centre for Translational Medicine, Semmelweis University, T≈±zolt√≥ utca 37-47

      1072, Budapest, Hungary; Institute of Pancreatic Diseases, Semmelweis University,
      T√∂m≈ë utca 25-29 1083, Budapest, Hungary; Institute for Translational Medicine,
      Medical School, University of P√©cs, Szigeti utca 12 7624, P√©cs, Hungary.
FAU - Kivovics, M√°rton
AU - Kivovics M
AD - Centre for Translational Medicine, Semmelweis University, T≈±zolt√≥ utca 37-47
      1072, Budapest, Hungary; Department of Community Dentistry, Semmelweis
      University, Szentkir√°lyi utca 40 1088, Budapest, Hungary. Electronic address:
      kivovics.marton@semmelweis.hu.
LA - eng
PT - Journal Article
PT - Meta-Analysis
PT - Systematic Review
DEP - 20241011
PL - England
TA - J Dent
JT - Journal of dentistry
JID - 0354422
SB - IM
MH - Humans
MH - *Artificial Intelligence
MH - *Dental Caries/diagnostic imaging
MH - *Radiography, Bitewing
MH - Sensitivity and Specificity
OTO - NOTNLM
OT - Approximal caries
OT - Artificial intelligence
OT - Bitewing radiographs
OT - Convolutional neural network
COIS- Declaration of competing interest The authors declare that they have no known
      competing financial interests or personal relationships that could have appeared
      to influence the work reported in this paper.
EDAT- 2024/10/14 04:18
MHDA- 2024/11/24 00:44
CRDT- 2024/10/13 19:19
PHST- 2024/06/25 00:00 [received]
PHST- 2024/09/13 00:00 [revised]
PHST- 2024/10/01 00:00 [accepted]
PHST- 2024/11/24 00:44 [medline]
PHST- 2024/10/14 04:18 [pubmed]
PHST- 2024/10/13 19:19 [entrez]
AID - S0300-5712(24)00558-X [pii]
AID - 10.1016/j.jdent.2024.105388 [doi]
PST - ppublish
SO - J Dent. 2024 Dec;151:105388. doi: 10.1016/j.jdent.2024.105388. Epub 2024 Oct 11.

PMID- 39373739
OWN - NLM
STAT- MEDLINE
DCOM- 20241007
LR - 20241117
IS - 1436-3771 (Electronic)
IS - 1432-6981 (Print)
IS - 1432-6981 (Linking)
VI - 28
IP - 11
DP - 2024 Oct 7
TI - Performance of large language artificial intelligence models on solving
      restorative dentistry and endodontics student assessments.
PG - 575
LID - 10.1007/s00784-024-05968-w [doi]
LID - 575
AB - OBJECTIVES: The advent of artificial intelligence (AI) and large language model
      (LLM)-based AI applications (LLMAs) has tremendous implications for our society.
      This study analyzed the performance of LLMAs on solving restorative dentistry and
      endodontics (RDE) student assessment questions. MATERIALS AND METHODS: 151
      questions from a RDE question pool were prepared for prompting using LLMAs from
      OpenAI (ChatGPT-3.5,-4.0 and -4.0o) and Google (Gemini 1.0). Multiple-choice
      questions were sorted into four question subcategories, entered into LLMAs and
      answers recorded for analysis. P-value and chi-square statistical analyses were
      performed using Python 3.9.16. RESULTS: The total answer accuracy of ChatGPT-4.0o
      was the highest, followed by ChatGPT-4.0, Gemini 1.0 and ChatGPT-3.5 (72%, 62%,
      44% and 25%, respectively) with significant differences between all LLMAs except
      GPT-4.0 models. The performance on subcategories direct restorations and caries
      was the highest, followed by indirect restorations and endodontics. CONCLUSIONS:
      Overall, there are large performance differences among LLMAs. Only the ChatGPT-4
      models achieved a success ratio that could be used with caution to support the
      dental academic curriculum. CLINICAL RELEVANCE: While LLMAs could support
      clinicians to answer dental field-related questions, this capacity depends
      strongly on the employed model. The most performant model ChatGPT-4.0o achieved

acceptable accuracy rates in some subject sub-categories analyzed.
CI - ¬© 2024. The Author(s).
FAU - K√°nzle, Paul
AU - K√°nzle P
AD - Department of Operative, Preventive and Pediatric Dentistry, Charit√© -
      Universit√§tsmedizin Berlin, A√ümannshauser Str. 4-6, Berlin, 14197, Germany.
      paul.kuenzle@charite.de.
FAU - Paris, Sebastian
AU - Paris S
AD - Department of Operative, Preventive and Pediatric Dentistry, Charit√© -
      Universit√§tsmedizin Berlin, A√ümannshauser Str. 4-6, Berlin, 14197, Germany.
LA - eng
PT - Journal Article
DEP - 20241007
PL - Germany
TA - Clin Oral Investig
JT - Clinical oral investigations
JID - 9707115
SB - IM
MH - Humans
MH - *Artificial Intelligence
MH - *Endodontics/education
MH - Education, Dental/methods
MH - Educational Measurement/methods
MH - Students, Dental
MH - Dentistry, Operative/education
MH - Clinical Competence
MH - Surveys and Questionnaires
PMC - PMC11458639
OTO - NOTNLM
OT - Artificial intelligence
OT - ChatGPT
OT - Gemini
OT - GenAI
OT - Natural language processing
COIS- No declared conflicts of interest exist among all authors of this study neither
      regarding authorship nor publication of this manuscript.
EDAT- 2024/10/07 12:57
MHDA- 2024/10/07 12:58
PMCR- 2024/10/07
CRDT- 2024/10/07 11:03
PHST- 2024/07/11 00:00 [received]
PHST- 2024/09/24 00:00 [accepted]
PHST- 2024/10/07 12:58 [medline]
PHST- 2024/10/07 12:57 [pubmed]
PHST- 2024/10/07 11:03 [entrez]
PHST- 2024/10/07 00:00 [pmc-release]
AID - 10.1007/s00784-024-05968-w [pii]
AID - 5968 [pii]

AID - 10.1007/s00784-024-05968-w [doi]
PST - epublish
SO - Clin Oral Investig. 2024 Oct 7;28(11):575. doi: 10.1007/s00784-024-05968-w.

PMID- 39329679
OWN - NLM
STAT- PubMed-not-MEDLINE
LR - 20240929
IS - 2306-5354 (Print)
IS - 2306-5354 (Electronic)
IS - 2306-5354 (Linking)
VI - 11
IP - 9
DP - 2024 Sep 18
TI - The Use of Artificial Intelligence in Caries Detection: A Review.
LID - 10.3390/bioengineering11090936 [doi]
LID - 936
AB - Advancements in artificial intelligence (AI) have significantly impacted the field of dentistry, particularly in diagnostic imaging for caries detection. This review critically examines the current state of AI applications in caries detection, focusing on the performance and accuracy of various AI techniques. We evaluated 40 studies from the past 23 years, carefully selected for their relevance and quality. Our analysis highlights the potential of AI, especially convolutional neural networks (CNNs), to improve diagnostic accuracy and efficiency in detecting dental caries. The findings underscore the transformative potential of AI in clinical dental practice.
FAU - Al-Khalifa, Khalifa S
AU - Al-Khalifa KS
AUID- ORCID: 0000-0001-8160-9288
AD - Department of Preventive Dental Sciences, College of Dentistry, Imam Abdulrahman Bin Faisal University, Dammam 31441, Saudi Arabia.
FAU - Ahmed, Walaa Magdy
AU - Ahmed WM
AUID- ORCID: 0000-0003-1810-8733
AD - Department of Restorative Dentistry, Faculty of Dentistry, King Abdulaziz University, Jeddah 21589, Saudi Arabia.
FAU - Azhari, Amr Ahmed
AU - Azhari AA
AUID- ORCID: 0000-0002-8749-4714
AD - Department of Restorative Dentistry, Faculty of Dentistry, King Abdulaziz University, Jeddah 21589, Saudi Arabia.
FAU - Qaw, Masoumah

AU - Qaw M
AUID- ORCID: 0000-0001-6047-9031
AD - Department of Restorative Dental Sciences, College of Dentistry, Imam Abdulrahman
      Bin Faisal University, Dammam 31441, Saudi Arabia.
FAU - Alsheikh, Rasha
AU - Alsheikh R
AUID- ORCID: 0000-0003-2910-5511
AD - Department of Restorative Dental Sciences, College of Dentistry, Imam Abdulrahman
      Bin Faisal University, Dammam 31441, Saudi Arabia.
FAU - Alqudaihi, Fatema
AU - Alqudaihi F
AUID- ORCID: 0000-0003-3161-9482
AD - Department of Restorative Dentistry, Khobar Dental Complex, Eastern Health
      Cluster, Dammam 32253, Saudi Arabia.
FAU - Alfaraj, Amal
AU - Alfaraj A
AD - Department of Prosthodontics and Dental Implantology, College of Dentistry, King
      Faisal University, Al-Ahsa 31982, Saudi Arabia.
LA - eng
PT - Journal Article
PT - Review
DEP - 20240918
PL - Switzerland
TA - Bioengineering (Basel)
JT - Bioengineering (Basel, Switzerland)
JID - 101676056
PMC - PMC11428802
OTO - NOTNLM
OT - artificial intelligence
OT - dental caries
OT - detection
OT - diagnosis
OT - treatment planning
COIS- The authors declare no conflicts of interest.
EDAT- 2024/09/27 16:17
MHDA- 2024/09/27 16:18
PMCR- 2024/09/18
CRDT- 2024/09/27 09:23
PHST- 2024/07/07 00:00 [received]
PHST- 2024/08/20 00:00 [revised]
PHST- 2024/09/11 00:00 [accepted]
PHST- 2024/09/27 16:18 [medline]
PHST- 2024/09/27 16:17 [pubmed]
PHST- 2024/09/27 09:23 [entrez]
PHST- 2024/09/18 00:00 [pmc-release]
AID - bioengineering11090936 [pii]
AID - bioengineering-11-00936 [pii]
AID - 10.3390/bioengineering11090936 [doi]
PST - epublish
SO - Bioengineering (Basel). 2024 Sep 18;11(9):936. doi:

10.3390/bioengineering11090936.

PMID- 39311330
OWN - NLM
STAT- PubMed-not-MEDLINE
LR - 20240925
IS - 2036-749X (Print)
IS - 2036-7503 (Electronic)
IS - 2036-749X (Linking)
VI - 16
IP - 3
DP - 2024 Sep 11
TI - Artificial Intelligence (AI) Assessment of Pediatric Dental Panoramic Radiographs (DPRs): A Clinical Study.
PG - 794-805
LID - 10.3390/pediatric16030067 [doi]
AB - This clinical study aimed to evaluate the sensitivity, specificity, accuracy, and precision of artificial intelligence (AI) in assessing permanent teeth in pediatric patients. Over one thousand consecutive DPRs taken in Kielce, Poland, with the Carestream CS9600 device were screened. In the study material, 35 dental panoramic radiographs (DPRs) of patients of developmental age were identified and included. They were automatically evaluated with an AI algorithm. The DPRs were then analyzed by researchers. The status of the following dichotomous variables was assessed: (1) decay, (2) missing tooth, (3) filled tooth, (4) root canal filling, and (5) endodontic lesion. The results showed high specificity and accuracy (all above 85%) in detecting caries, dental fillings, and missing teeth but low precision. This study provided a detailed assessment of AI performance in a previously neglected age group. In conclusion, the overall accuracy of AI algorithms for evaluating permanent dentition in dental panoramic radiographs is lower for pediatric patients than adults or the entire population. Hence, identifying primary teeth should be implemented in AI-driven software, at least so as to ignore them when assessing mixed dentition (ClinicalTrials.gov registration number: NCT06258798).
FAU - Turosz, Natalia
AU - Turosz N
AUID- ORCID: 0000-0001-8075-9989

AD - Department of Maxillofacial Surgery, Hospital of the Ministry of Interior, Wojska
      Polskiego 51, 25-375 Kielce, Poland.
FAU - Chƒôci≈Ñska, Kamila
AU - Chƒôci≈Ñska K
AUID- ORCID: 0000-0002-5113-9817
AD - Department of Glass Technology and Amorphous Coatings, Faculty of Materials
      Science and Ceramics, AGH University of Science and Technology, Mickiewicza 30,
      30-059 Krak√≥w, Poland.
FAU - Chƒôci≈Ñski, Maciej
AU - Chƒôci≈Ñski M
AUID- ORCID: 0000-0002-6199-4753
AD - Department of Oral Surgery, Preventive Medicine Center, Komorowskiego 12, 30-106
      Krak√≥w, Poland.
FAU - Lubecka, Karolina
AU - Lubecka K
AUID- ORCID: 0009-0004-6758-5450
AD - Department of Oral Surgery, Preventive Medicine Center, Komorowskiego 12, 30-106
      Krak√≥w, Poland.
FAU - Bli≈∫niak, Filip
AU - Bli≈∫niak F
AUID- ORCID: 0009-0009-4985-7113
AD - Department of Oral Surgery, Preventive Medicine Center, Komorowskiego 12, 30-106
      Krak√≥w, Poland.
FAU - Sikora, Maciej
AU - Sikora M
AUID- ORCID: 0000-0002-3348-1950
AD - Department of Maxillofacial Surgery, Hospital of the Ministry of Interior, Wojska
      Polskiego 51, 25-375 Kielce, Poland.
AD - Department of Biochemistry and Medical Chemistry, Pomeranian Medical University,
      Powsta≈Ñc√≥w Wielkopolskich 72, 70-111 Szczecin, Poland.
LA - eng
SI - ClinicalTrials.gov/NCT06258798
PT - Journal Article
DEP - 20240911
PL - Switzerland
TA - Pediatr Rep
JT - Pediatric reports
JID - 101551542
PMC - PMC11417896
OTO - NOTNLM
OT - DMF index
OT - artificial intelligence
OT - dental caries
OT - panoramic radiography
COIS- The authors declare no conflicts of interest.
EDAT- 2024/09/23 12:46

MHDA- 2024/09/23 12:47
PMCR- 2024/09/11
CRDT- 2024/09/23 07:25
PHST- 2024/07/08 00:00 [received]
PHST- 2024/08/26 00:00 [revised]
PHST- 2024/09/04 00:00 [accepted]
PHST- 2024/09/23 12:47 [medline]
PHST- 2024/09/23 12:46 [pubmed]
PHST- 2024/09/23 07:25 [entrez]
PHST- 2024/09/11 00:00 [pmc-release]
AID - pediatric16030067 [pii]
AID - pediatrrep-16-00067 [pii]
AID - 10.3390/pediatric16030067 [doi]
PST - epublish
SO - Pediatr Rep. 2024 Sep 11;16(3):794-805. doi: 10.3390/pediatric16030067.

PMID- 39206581
OWN - NLM
STAT- MEDLINE
DCOM- 20240829
LR - 20240916
IS - 2057-4347 (Electronic)
IS - 2057-4347 (Linking)
VI - 10
IP - 4
DP - 2024 Aug
TI - Artificial Intelligence in Dental Caries Diagnosis and Detection: An Umbrella Review.
PG - e70004
LID - 10.1002/cre2.70004 [doi]
LID - e70004
AB - BACKGROUND AND AIM: Dental caries is largely preventable, yet an important global health issue. Numerous systematic reviews have summarized the efficacy of artificial intelligence (AI) models for the diagnosis and detection of dental caries. Therefore, this umbrella review aimed to synthesize the results of systematic reviews on the application and effectiveness of AI models in diagnosing and detecting dental caries. METHODS: MEDLINE/PubMed, IEEE Explore, Embase, and Cochrane Database of Systematic Reviews were searched to retrieve studies. Two authors independently screened the articles based on eligibility criteria and then, appraised the included articles. The findings are summarized in tabulation form and discussed using the narrative method. RESULT: A total of

1249 entries were identified out of which 7 were finally included. The most often
employed AI algorithms were the multilayer perceptron, support vector machine
(SVM), and neural networks. The algorithms were built to perform the
segmentation, classification, caries detection, diagnosis, and caries prediction
from several sources, including periapical radiographs, panoramic radiographs,
smartphone images, bitewing radiographs, near-infrared light transillumination
images, and so forth. Convoluted neural networks (CNN) demonstrated high
sensitivity, specificity, and area under the curve in the caries detection,
segmentation, and classification tests. Notably, AI in conjunction with
periapical and panoramic radiography images yielded better accuracy in detecting
and diagnosing dental caries. CONCLUSION: AI models, especially convolutional
neural network (CNN)-based models, have an enormous amount of potential for
accurate, objective dental caries diagnosis and detection. However, ethical
considerations and cautious adoption remain critical to its successful
integration into routine practice.
CI - ¬© 2024 The Author(s). Clinical and Experimental Dental Research published by John
Wiley & Sons Ltd.
FAU - Negi, Sapna
AU - Negi S
AD - Department of Dental Research Cell, Dr. D. Y. Patil Dental College and Hospital,
Dr. D. Y. Patil Vidyapeeth, Pune, Maharashtra, India.
FAU - Mathur, Ankita
AU - Mathur A
AD - Department of Dental Research Cell, Dr. D. Y. Patil Dental College and Hospital,
Dr. D. Y. Patil Vidyapeeth, Pune, Maharashtra, India.
FAU - Tripathy, Snehasish
AU - Tripathy S
AD - Department of Dental Research Cell, Dr. D. Y. Patil Dental College and Hospital,
Dr. D. Y. Patil Vidyapeeth, Pune, Maharashtra, India.
FAU - Mehta, Vini
AU - Mehta V
AD - Department of Dental Research Cell, Dr. D. Y. Patil Dental College and Hospital,
Dr. D. Y. Patil Vidyapeeth, Pune, Maharashtra, India.
FAU - Snigdha, Niher Tabassum
AU - Snigdha NT

AD - Department of Dental Research, Saveetha Medical College and Hospitals, Saveetha
      Institute of Medical and Technical Sciences, Saveetha University, Chennai, Tamil
      Nadu, India.
FAU - Adil, Abdul Habeeb
AU - Adil AH
AD - Department of Dental Research, Saveetha Medical College and Hospitals, Saveetha
      Institute of Medical and Technical Sciences, Saveetha University, Chennai, Tamil
      Nadu, India.
FAU - Karobari, Mohmed Isaqali
AU - Karobari MI
AUID- ORCID: 0000-0002-0313-9695
AD - Department of Dental Research, Saveetha Medical College and Hospitals, Saveetha
      Institute of Medical and Technical Sciences, Saveetha University, Chennai, Tamil
      Nadu, India.
AD - Department of Restorative Dentistry & Endodontics, Faculty of Dentistry,
      University of Puthisastra, Phnom Penh, Cambodia.
LA - eng
GR - The authors received no specific funding for this work./
PT - Journal Article
PT - Systematic Review
PL - United States
TA - Clin Exp Dent Res
JT - Clinical and experimental dental research
JID - 101692332
SB - IM
MH - Humans
MH - *Artificial Intelligence
MH - *Dental Caries/diagnostic imaging
MH - Neural Networks, Computer
MH - Radiography, Panoramic/methods
MH - Support Vector Machine
MH - Systematic Reviews as Topic
PMC - PMC11358700
OTO - NOTNLM
OT - artificial intelligence
OT - dental caries
OT - neural networks
OT - umbrella review
COIS- The authors declare no conflicts of interest.
EDAT- 2024/08/31 09:48
MHDA- 2024/08/31 09:49
PMCR- 2024/08/29
CRDT- 2024/08/29 05:23
PHST- 2024/04/29 00:00 [revised]
PHST- 2023/10/18 00:00 [received]
PHST- 2024/08/16 00:00 [accepted]
PHST- 2024/08/31 09:49 [medline]

PHST- 2024/08/31 09:48 [pubmed]
PHST- 2024/08/29 05:23 [entrez]
PHST- 2024/08/29 00:00 [pmc-release]
AID - CRE270004 [pii]
AID - 10.1002/cre2.70004 [doi]
PST - ppublish
SO - Clin Exp Dent Res. 2024 Aug;10(4):e70004. doi: 10.1002/cre2.70004.

PMID- 39068241
OWN - NLM
STAT- PubMed-not-MEDLINE
LR - 20240730
IS - 2398-6352 (Electronic)
IS - 2398-6352 (Linking)
VI - 7
IP - 1
DP - 2024 Jul 27
TI - Expert gaze as a usability indicator of medical AI decision support systems: a
      preliminary study.
PG - 199
LID - 10.1038/s41746-024-01192-8 [doi]
LID - 199
AB - Given the current state of medical artificial intelligence (AI) and perceptions
      towards it, collaborative systems are becoming the preferred choice for clinical
      workflows. This work aims to address expert interaction with medical AI support
      systems to gain insight towards how these systems can be better designed with the
      user in mind. As eye tracking metrics have been shown to be robust indicators of
      usability, we employ them for evaluating the usability and user interaction with
      medical AI support systems. We use expert gaze to assess experts' interaction
      with an AI software for caries detection in bitewing x-ray images. We compared
      standard viewing of bitewing images without AI support versus viewing where AI
      support could be freely toggled on and off. We found that experts turned the AI
      on for roughly 25% of the total inspection task, and generally turned it on
      halfway through the course of the inspection. Gaze behavior showed that when
      supported by AI, more attention was dedicated to user interface elements related
      to the AI support, with more frequent transitions from the image itself to these
      elements. When considering that expert visual strategy is already optimized for

fast and effective image inspection, such interruptions in attention can lead to increased time needed for the overall assessment. Gaze analysis provided valuable insights into an AI's usability for medical image inspection. Further analyses of these tools and how to delineate metrical measures of usability should be developed.
CI - ¬© 2024. The Author(s).
FAU - Castner, Nora
AU - Castner N
AUID- ORCID: 0000-0002-6771-7693
AD - Carl Zeiss Vision International GmbH, T√°bingen, Germany. nora.castner@zeiss.com.
AD - University of T√°bingen, T√°bingen, Germany. nora.castner@zeiss.com.
FAU - Arsiwala-Scheppach, Lubaina
AU - Arsiwala-Scheppach L
AD - Charit√© - Univesit√§tsmedizin, Oral Diagnostics, Digital Health and Services Research, Berlin, Germany.
FAU - Mertens, Sarah
AU - Mertens S
AD - Charit√© - Univesit√§tsmedizin, Oral Diagnostics, Digital Health and Services Research, Berlin, Germany.
FAU - Krois, Joachim
AU - Krois J
AUID- ORCID: 0000-0002-6010-8940
AD - Charit√© - Univesit√§tsmedizin, Oral Diagnostics, Digital Health and Services Research, Berlin, Germany.
FAU - Thaqi, Enkeleda
AU - Thaqi E
AD - Technical University of Munich, Human-Centered Technologies for Learning, Munich, Germany.
FAU - Kasneci, Enkelejda
AU - Kasneci E
AD - Technical University of Munich, Human-Centered Technologies for Learning, Munich, Germany.
FAU - Wahl, Siegfried
AU - Wahl S
AUID- ORCID: 0000-0003-3437-6711
AD - Carl Zeiss Vision International GmbH, T√°bingen, Germany.
AD - Institute for Ophthalmic Research, University of T√°bingen, T√°bingen, Germany.
FAU - Schwendicke, Falk
AU - Schwendicke F
AD - Ludwig Maximilian University, Operative, Preventative and Pediatric Dentistry and Periodontology, Munich, Germany.

LA - eng
PT - Journal Article
DEP - 20240727
PL - England
TA - NPJ Digit Med
JT - NPJ digital medicine
JID - 101731738
PMC - PMC11283514
COIS- F.S. and J.K. are co-founders of the startup dentalXrai GmbH. dentalXrai GmbH had
      no role in the design of the study; in the collection, analyses, or
      interpretation of data; in the writing of the manuscript; or in the decision to
      publish the results. The remaining authors declare no competing interests.
EDAT- 2024/07/28 14:48
MHDA- 2024/07/28 14:49
PMCR- 2024/07/27
CRDT- 2024/07/27 23:20
PHST- 2023/11/10 00:00 [received]
PHST- 2024/07/12 00:00 [accepted]
PHST- 2024/07/28 14:49 [medline]
PHST- 2024/07/28 14:48 [pubmed]
PHST- 2024/07/27 23:20 [entrez]
PHST- 2024/07/27 00:00 [pmc-release]
AID - 10.1038/s41746-024-01192-8 [pii]
AID - 1192 [pii]
AID - 10.1038/s41746-024-01192-8 [doi]
PST - epublish
SO - NPJ Digit Med. 2024 Jul 27;7(1):199. doi: 10.1038/s41746-024-01192-8.

PMID- 38999252
OWN - NLM
STAT- PubMed-not-MEDLINE
LR - 20240715
IS - 2077-0383 (Print)
IS - 2077-0383 (Electronic)
IS - 2077-0383 (Linking)
VI - 13
IP - 13
DP - 2024 Jun 25
TI - Oral Health Status and Treatment Needs Based on Artificial Intelligence (AI)
      Dental Panoramic Radiograph (DPR) Analysis: A Cross-Sectional Study.
LID - 10.3390/jcm13133686 [doi]
LID - 3686
AB - Background: The application of artificial intelligence (AI) is gaining popularity
      in modern dentistry. AI has been successfully used to interpret dental panoramic
      radiographs (DPRs) and quickly screen large groups of patients. This

cross-sectional study aimed to perform a population-based assessment of the oral health status and treatment needs of the residents of Kielce, Poland, and the surrounding area based on DPR analysis performed by a high-accuracy AI algorithm trained with over 250,000 radiographs. Methods: This study included adults who had a panoramic radiograph performed, regardless of indications. The following diagnoses were used for analysis: (1) dental caries, (2) missing tooth, (3) dental filling, (4) root canal filling, (5) endodontic lesion, (6) implant, (7) implant abutment crown, (8) pontic crown, (9) dental abutment crown, and (10) sound tooth. The study sample included 980 subjects. Results: The patients had an average of 15 sound teeth, with the domination of the lower dental arch over the upper one. The most commonly identified pathology was dental caries, which affected 99% of participants. A total of 67% of patients underwent root canal treatment. Every fifth endodontically treated tooth presented a periapical lesion. Of study group members, 82% lost at least one tooth. Pontics were identified more often (9%) than implants (2%) in replacing missing teeth. Conclusions: DPR assessment by AI has proven to be an efficient method for population analysis. Despite recent improvements in the oral health status of Polish residents, its level is still unsatisfactory and suggests the need to improve oral health. However, due to some limitations of this study, the results should be interpreted with caution.
FAU - Turosz, Natalia
AU - Turosz N
AUID- ORCID: 0000-0001-8075-9989
AD - Department of Maxillofacial Surgery, Hospital of the Ministry of Interior, Wojska Polskiego 51, 25-375 Kielce, Poland.
FAU - Chƒôci≈Ñska, Kamila
AU - Chƒôci≈Ñska K
AUID- ORCID: 0000-0002-5113-9817
AD - Department of Glass Technology and Amorphous Coatings, Faculty of Materials Science and Ceramics, AGH University of Science and Technology, Mickiewicza 30, 30-059 Cracow, Poland.
FAU - Chƒôci≈Ñski, Maciej

AU - Chƒôci≈Ñski M
AUID- ORCID: 0000-0002-6199-4753
AD - Department of Oral Surgery, Preventive Medicine Center, Komorowskiego 12, 30-106
      Cracow, Poland.
FAU - Ruta≈Ñski, Iwo
AU - Ruta≈Ñski I
AUID- ORCID: 0009-0005-4548-5819
AD - Optident sp. z o.o., ul. Eugeniusza Kwiatkowskiego 4, 52-326 Wroclaw, Poland.
FAU - Sielski, Marcin
AU - Sielski M
AUID- ORCID: 0000-0002-7130-5480
AD - Department of Maxillofacial Surgery, Hospital of the Ministry of Interior, Wojska
      Polskiego 51, 25-375 Kielce, Poland.
FAU - Sikora, Maciej
AU - Sikora M
AUID- ORCID: 0000-0002-3348-1950
AD - Department of Maxillofacial Surgery, Hospital of the Ministry of Interior, Wojska
      Polskiego 51, 25-375 Kielce, Poland.
AD - Department of Biochemistry and Medical Chemistry, Pomeranian Medical University,
      Powsta≈Ñc√≥w Wielkopolskich 72, 70-111 Szczecin, Poland.
LA - eng
PT - Journal Article
DEP - 20240625
PL - Switzerland
TA - J Clin Med
JT - Journal of clinical medicine
JID - 101606588
PMC - PMC11242788
OTO - NOTNLM
OT - DMF Index
OT - artificial intelligence
OT - dental radiography
OT - panoramic radiography
OT - public health dentistry
COIS- One of the authors, Iwo Ruta≈Ñski, is a service manager and the head of training
      at a company that offers X-ray equipment and software, which we used in this
      population-based study. The other authors declare no conflicts of interest.
EDAT- 2024/07/13 07:45
MHDA- 2024/07/13 07:46
PMCR- 2024/06/25
CRDT- 2024/07/13 01:09
PHST- 2024/05/30 00:00 [received]
PHST- 2024/06/20 00:00 [revised]
PHST- 2024/06/21 00:00 [accepted]
PHST- 2024/07/13 07:46 [medline]
PHST- 2024/07/13 07:45 [pubmed]

PHST- 2024/07/13 01:09 [entrez]
PHST- 2024/06/25 00:00 [pmc-release]
AID - jcm13133686 [pii]
AID - jcm-13-03686 [pii]
AID - 10.3390/jcm13133686 [doi]
PST - epublish
SO - J Clin Med. 2024 Jun 25;13(13):3686. doi: 10.3390/jcm13133686.

PMID- 38755978
OWN - NLM
STAT- MEDLINE
DCOM- 20240517
LR - 20240529
IS - 1557-5268 (Electronic)
IS - 1053-4628 (Linking)
VI - 48
IP - 3
DP - 2024 May
TI - Role of artificial intelligence in behavior management of pediatric dental
      patients-a mini review.
PG - 24-30
LID - 10.22514/jocpd.2024.055 [doi]
AB - The influence of behavioral science on various organizations has been
      experiencing remarkable growth worldwide. With the integration of recent
      technological advancements, behavioral science's impact has expanded into diverse
      fields such as finance and policy. The term "artificial intelligence" (AI) has
      become increasingly prevalent, but it is essential to provide clarity before
      proceeding. AI pertains to the theory and creation of systems capable of
      executing tasks that typically necessitate human intelligence. Integrating
      artificial intelligence (AI) in pediatric dentistry has emerged as a promising
      avenue to enhance patient care, improve diagnostic accuracy, streamline treatment
      planning, and augment patient engagement. AI-driven tools such as image analysis,
      natural language processing, and machine learning algorithms assist in early
      caries detection, orthodontic treatment planning, behavior management, and
      personalized oral hygiene education for pediatric patients. This paper presents
      an overview of AI's applications in pediatric dentistry, particularly behavior
      management, highlighting its potential to revolutionize traditional pediatric
      dental practices.

CI - ¬©2024 The Author(s). Published by MRE Press.
FAU - Acharya, Sonu
AU - Acharya S
AD - Pediatric and Preventive Dentistry, Institute of Dental Sciences, Siksha
      'O'Anusandhan (Deemed to be) University, 751016 Bhubaneswar, India.
FAU - Godhi, Brinda S
AU - Godhi BS
AD - Department of Pediatric and Preventive Dentistry, JSS Dental College, JSS Academy
      of Higher Education and research, 570015 Mysuru, India.
FAU - Saxena, Vrinda
AU - Saxena V
AD - Department of Public Health Dentistry, Government Dental College, 452001 Indore,
      India.
FAU - Assiry, Ali A
AU - Assiry AA
AD - Preventive Dental Science Department, Faculty of Dentistry, Najran University,
      1988 Najran, Kingdom of Saudi Arabia.
FAU - Alessa, Noura Abdulaziz
AU - Alessa NA
AD - Department of pediatric dentistry and orthodontics, dental college, King Saud
      University, 11454 Riyadh, Kingdom of Saudi Arabia.
FAU - Dawasaz, Ali Azhar
AU - Dawasaz AA
AD - Department of diagnostic dental sciences, College of dentistry, King Khalid
      University, 61421 Abha, Kingdom of Saudi Arabia.
FAU - Alqarni, Abdullah
AU - Alqarni A
AD - Department of Diagnostics Dental Sciences and Oral Biology, College of Dentistry,
      King Khalid University, 61421 Abha, Kingdom of Saudi Arabia.
FAU - Karobari, Mohmed Isaqali
AU - Karobari MI
AD - Dental Research Unit, Center for Global Health Research, Saveetha Medical College
      and Hospitals, Saveetha Institute of Medical and Technical Sciences, Saveetha
      University, 600077 Chennai, India.
LA - eng
PT - Journal Article
PT - Review
DEP - 20240503
PL - Singapore
TA - J Clin Pediatr Dent
JT - The Journal of clinical pediatric dentistry
JID - 9100079
SB - IM
MH - Humans
MH - *Artificial Intelligence

MH - Child
MH - *Pediatric Dentistry
MH - Dental Care for Children/methods
OTO - NOTNLM
OT - Artificial intelligence
OT - Behavior
OT - Children
OT - Management
OT - New technology
OT - Pediatric dentistry
COIS- The authors declare no conflict of interest.
EDAT- 2024/05/17 06:43
MHDA- 2024/05/17 06:44
CRDT- 2024/05/17 01:33
PHST- 2023/07/20 00:00 [received]
PHST- 2023/09/18 00:00 [accepted]
PHST- 2024/05/17 06:44 [medline]
PHST- 2024/05/17 06:43 [pubmed]
PHST- 2024/05/17 01:33 [entrez]
AID - S1053-4628(24)00138-3 [pii]
AID - 10.22514/jocpd.2024.055 [doi]
PST - ppublish
SO - J Clin Pediatr Dent. 2024 May;48(3):24-30. doi:
10.22514/jocpd.2024.055. Epub
      2024 May 3.

PMID- 38743125
OWN - NLM
STAT- MEDLINE
DCOM- 20241202
LR - 20241205
IS - 2948-2933 (Electronic)
IS - 2948-2925 (Print)
IS - 2948-2925 (Linking)
VI - 37
IP - 6
DP - 2024 Dec
TI - AI-Assisted Detection of Interproximal, Occlusal, and Secondary
Caries on
      Bite-Wing Radiographs: A Single-Shot Deep Learning Approach.
PG - 3146-3159
LID - 10.1007/s10278-024-01113-x [doi]
AB - Tooth decay is a common oral disease worldwide, but errors in
diagnosis can often
      be made in dental clinics, which can lead to a delay in treatment.
This study
      aims to use artificial intelligence (AI) for the automated
detection and
      localization of secondary, occlusal, and interproximal (D1, D2, D3)
caries types
      on bite-wing radiographs. The eight hundred¬†and sixty bite-wing
radiographs were
      collected from the School of Dentistry database. Pre-processing and
data

augmentation operations were performed. Interproximal (D1, D2, D3), secondary, and occlusal caries on bite-wing radiographs were annotated by two oral radiologists. The data were split into 80% for training, 10% for validation, and 10% for testing. The AI-based training process was conducted using the YOLOv8 algorithm. A clinical decision support system interface was designed using the Python PyQT5 library, allowing for the use of dental caries detection without the need for complex programming procedures. In the test images, the average precision, average sensitivity, and average F1 score values for secondary, occlusal, and interproximal caries were obtained as 0.977, 0.932, and 0.954, respectively. The AI-based dental caries detection system yielded highly successful results in the test, receiving full approval from dentists for clinical use. YOLOv8 has the potential to increase sensitivity and reliability while reducing the burden on dentists and can prevent diagnostic errors in dental clinics.
CI - ¬© 2024. The Author(s) under exclusive licence to Society for Imaging Informatics in Medicine.
FAU - Karaku≈ü, Rabia
AU - Karaku≈ü R
AUID- ORCID: 0000-0002-2294-8551
AD - Faculty of Dentistry, Department of Oral and Maxillofacial Radiology, Necmettin Erbakan University, Konya, Turkey.
FAU - √ñzi√ß, Muhammet √úsame
AU - √ñzi√ß M√ú
AUID- ORCID: 0000-0002-3037-2687
AD - Faculty of Technology, Department of Biomedical Engineering, Pamukkale University, Denizli, Turkey. muozic@pau.edu.tr.
FAU - Tassoker, Melek
AU - Tassoker M
AUID- ORCID: 0000-0003-2062-5713
AD - Faculty of Dentistry, Department of Oral and Maxillofacial Radiology, Necmettin Erbakan University, Konya, Turkey.
LA - eng
PT - Journal Article
DEP - 20240514
PL - Switzerland
TA - J Imaging Inform Med
JT - Journal of imaging informatics in medicine

JID - 9918663679206676
SB - IM
MH - Humans
MH - *Dental Caries/diagnostic imaging
MH - *Deep Learning
MH - Artificial Intelligence
MH - Radiography, Bitewing/methods
MH - Algorithms
MH - Adult
PMC - PMC11612078
OTO - NOTNLM
OT - Artificial intelligence
OT - Deep learning
OT - Dental caries
OT - Detection
OT - GUI
OT - YOLOv8
COIS- Declarations. Ethical Approval: This study was conducted at the Faculty of
      Dentistry, Necmettin Erbakan University, Department of Dentomaxillofacial
      Radiology, with the approval of the Ethics Committee (dated 30 June 2022 and
      numbered 12,Äì94) and was performed according to the stipulations laid out by the
      Declaration of Helsinki. Consent to Participate: It is a retrospective study.
      Consent for Publication: There is no need. Conflict of Interest: The authors
      declare no competing interests.
EDAT- 2024/05/15 05:43
MHDA- 2024/12/03 00:23
PMCR- 2024/05/14
CRDT- 2024/05/14 11:05
PHST- 2024/02/23 00:00 [received]
PHST- 2024/04/01 00:00 [accepted]
PHST- 2024/03/28 00:00 [revised]
PHST- 2024/12/03 00:23 [medline]
PHST- 2024/05/15 05:43 [pubmed]
PHST- 2024/05/14 11:05 [entrez]
PHST- 2024/05/14 00:00 [pmc-release]
AID - 10.1007/s10278-024-01113-x [pii]
AID - 1113 [pii]
AID - 10.1007/s10278-024-01113-x [doi]
PST - ppublish
SO - J Imaging Inform Med. 2024 Dec;37(6):3146-3159. doi: 10.1007/s10278-024-01113-x.
      Epub 2024 May 14.

PMID- 38711998
OWN - NLM
STAT- PubMed-not-MEDLINE
LR - 20240508
IS - 0973-2063 (Print)

IS - 0973-2063 (Electronic)
IS - 0973-2063 (Linking)
VI - 20
IP - 3
DP - 2024
TI - Artificial intelligence enabled smart phone app for real-time caries detection on
      bitewing radiographs.
PG - 243-247
LID - 10.6026/973206300200243 [doi]
AB - Diagnosis of proximal caries is a difficult task. Artificial intelligence (AI)
      enabled diagnosis is gaining momentum. Therefore, it is of interest to evaluate
      the effectiveness of an artificial intelligence (AI) smart phone application for
      bitewing radiography towards real-time caries lesion detection. The Efficient
      Det-Lite1 artificial neural network was used after training 100 radiographic
      images obtained from the department of Oral Medicine. Trained model was then
      installed in a Google Pixel 6 (GP6) smartphone as artificial intelligence app.
      The back-facing mobile phone video camera of GP6 was utilised to detect caries
      lesions on 100 bitewing radiographs (BWR) with 80 carious lesion in real-time.
      Two different techniques such as scanning the static BWR on laptop with a moving
      mobile and scanning the moving radiograph on the laptop with stationery mobile
      were used. The average value of sensitivity/precision/F1 scores for both the
      techniques was 0.75/0.846 and 0.795 respectively. AI programme using the
      rear-facing mobile phone video camera was found to detect 75% of caries lesions
      in real time on 100 BWR with a precision of 84.6%. Thus, the use of AI with smart
      phone app is useful for caries diagnosis which is readily accessible, easy to use
      and fast.
CI - ¬© 2024 Biomedical Informatics.
FAU - Dhanak, Nupur
AU - Dhanak N
AD - Department of Conservative Dentistry and Endodontics, Government Dental College
      and Hospital, Ahmadabad, Gujarat, India.
FAU - Chougule, Vaibhav T
AU - Chougule VT
AD - Department of Paediatric and Preventive Dentistry, Bharati Vidyapeeth (Deemed to

be University) Dental College and Hospital, Sangli, Maharashtra, India.
FAU - Nalluri, Keerthi
AU - Nalluri K
AD - Apex, North Carolina, USA.
FAU - Kakkad, Ankur
AU - Kakkad A
AD - Department of Oral Medicine and Radiology, Hitkarini Dental College and Hospital,
Jabalpur, MP, India.
FAU - Dhimole, Ankit
AU - Dhimole A
AD - Department of Oral Medicine and Radiology, Hitkarini Dental College and Hospital,
Jabalpur, MP, India.
FAU - Parihar, Anuj Singh
AU - Parihar AS
AD - Department of Periodontology, People's Dental Academy, Bhopal, Madhya Pradesh,
India.
LA - eng
PT - Journal Article
DEP - 20240331
PL - Singapore
TA - Bioinformation
JT - Bioinformation
JID - 101258255
PMC - PMC11069605
OTO - NOTNLM
OT - Artificial intelligence
OT - bitewing radiograph
OT - caries detection
OT - mobile phone
EDAT- 2024/05/07 06:42
MHDA- 2024/05/07 06:43
PMCR- 2024/01/01
CRDT- 2024/05/07 03:44
PHST- 2024/03/01 00:00 [received]
PHST- 2024/03/31 00:00 [revised]
PHST- 2024/03/31 00:00 [accepted]
PHST- 2024/05/07 06:43 [medline]
PHST- 2024/05/07 06:42 [pubmed]
PHST- 2024/05/07 03:44 [entrez]
PHST- 2024/01/01 00:00 [pmc-release]
AID - 973206300200243 [pii]
AID - 10.6026/973206300200243 [doi]
PST - epublish
SO - Bioinformation. 2024 Mar 31;20(3):243-247. doi: 10.6026/973206300200243.
eCollection 2024.

PMID- 38595520
OWN - NLM
STAT- PubMed-not-MEDLINE

LR - 20240411
IS - 0976-4879 (Print)
IS - 0975-7406 (Electronic)
IS - 0975-7406 (Linking)
VI - 16
IP - Suppl 1
DP - 2024 Feb
TI - AI-Based Detection of Dental Caries: Comparative Analysis with Clinical
Examination.
PG - S580-S582
LID - 10.4103/jpbs.jpbs_872_23 [doi]
AB - Dental caries pose a significant public health concern, affecting a vast
population globally. Traditional clinical examination methods, although reliable,
can be subject to human error and time-consuming. Artificial intelligence (AI)
technologies have emerged as promising tools to enhance diagnostic accuracy and
efficiency. This study explores the potential of AI in revolutionizing dental
caries detection. MATERIALS AND METHODS: A cohort of 50 patients with varying
degrees of dental caries participated in this comparative analysis. Clinical
examination by dental professionals served as the gold standard for caries
detection. AI algorithms were trained using dental images, and their performance
was evaluated against the clinical examination results. RESULTS: The AI-based
detection system demonstrated a sensitivity of 92% and a specificity of 85% in
identifying dental caries, with an overall accuracy of 88%. The clinical
examination yielded a sensitivity of 86% and a specificity of 90%, resulting in
an overall accuracy of 88%. Statistical analysis indicated no significant
difference between AI-based detection and clinical examination (P > 0.05).
CONCLUSION: AI technology exhibits promise as an adjunctive tool for dental
practitioners, potentially reducing diagnostic errors and improving efficiency.
Integrating AI into routine dental practice may aid in early caries detection and
promote better oral health outcomes.
CI - Copyright: ¬© 2024 Journal of Pharmacy and Bioallied Sciences.
FAU - Alam, Mohammad K
AU - Alam MK
AD - Department of Preventive Dentistry, College of Dentistry, Jouf University,

      Sakaka, Saudi Arabia.
AD - Department of Dental Research Cell, Saveetha Institute of Medical and Technical
      Sciences, Saveetha Dental College and Hospitals, Chennai, Tamil Nadu, India.
AD - Department of Public Health, Faculty of Allied Health Sciences, Daffodil
      International University, Dhaka, Bangladesh.
FAU - Alanazi, Nawadir H
AU - Alanazi NH
AD - Department of Preventive Dentistry, College of Dentistry, Jouf University,
      Sakaka, Saudi Arabia.
FAU - Alazmi, Mona S
AU - Alazmi MS
AD - Department of Preventive Dentistry, College of Dentistry, Jouf University,
      Sakaka, Saudi Arabia.
FAU - Nagarajappa, Anil K
AU - Nagarajappa AK
AD - Department of Oral Surgery and Maxillofacial Diagnostics, College of Dentistry,
      Jouf University, Sakaka, Saudi Arabia.
LA - eng
PT - Journal Article
DEP - 20240229
PL - India
TA - J Pharm Bioallied Sci
JT - Journal of pharmacy & bioallied sciences
JID - 101537209
PMC - PMC11000941
OTO - NOTNLM
OT - AI-based detection
OT - clinical examination
OT - comparative analysis
OT - dental caries
OT - diagnostic accuracy
OT - oral health
COIS- There are no conflicts of interest.
EDAT- 2024/04/10 06:42
MHDA- 2024/04/10 06:43
PMCR- 2024/02/01
CRDT- 2024/04/10 03:56
PHST- 2023/09/09 00:00 [received]
PHST- 2023/09/14 00:00 [revised]
PHST- 2023/09/23 00:00 [accepted]
PHST- 2024/04/10 06:43 [medline]
PHST- 2024/04/10 06:42 [pubmed]
PHST- 2024/04/10 03:56 [entrez]
PHST- 2024/02/01 00:00 [pmc-release]
AID - JPBS-16-580 [pii]
AID - 10.4103/jpbs.jpbs_872_23 [doi]
PST - ppublish
SO - J Pharm Bioallied Sci. 2024 Feb;16(Suppl 1):S580-S582. doi:

10.4103/jpbs.jpbs_872_23. Epub 2024 Feb 29.

PMID- 38317889
OWN - NLM
STAT- PubMed-not-MEDLINE
LR - 20240207
IS - 2405-8440 (Print)
IS - 2405-8440 (Electronic)
IS - 2405-8440 (Linking)
VI - 10
IP - 3
DP - 2024 Feb 15
TI - Applications of artificial intelligence in the utilisation of imaging modalities in dentistry: A systematic review and meta-analysis of in-vitro studies.
PG - e24221
LID - 10.1016/j.heliyon.2024.e24221 [doi]
LID - e24221
AB - BACKGROUND: In the past, dentistry heavily relied on manual image analysis and diagnostic procedures, which could be time-consuming and prone to human error. The advent of artificial intelligence (AI) has brought transformative potential to the field, promising enhanced accuracy and efficiency in various dental imaging tasks. This systematic review and meta-analysis aimed to comprehensively evaluate the applications of AI in dental imaging modalities, focusing on in-vitro studies. METHODS: A systematic literature search was conducted, in accordance with the PRISMA guidelines. The following databases were systematically searched: PubMed/MEDLINE, Embase, Web of Science, Scopus, IEEE Xplore, Cochrane Library, CINAHL (Cumulative Index to Nursing and Allied Health Literature), and Google Scholar. The meta-analysis employed fixed-effects models to assess AI accuracy, calculating odds ratios (OR) for true positive rate (TPR), true negative rate (TNR), positive predictive value (PPV), and negative predictive value (NPV) with 95¬†% confidence intervals (CI). Heterogeneity and overall effect tests were applied to ensure the reliability of the findings. RESULTS: 9 studies were selected that encompassed various objectives, such as tooth segmentation and classification, caries detection, maxillofacial bone segmentation, and 3D surface model creation. AI techniques included convolutional

neural networks (CNNs), deep learning algorithms, and AI-driven tools. Imaging
parameters assessed in these studies were specific to the respective dental
tasks. The analysis of combined ORs indicated higher odds of accurate dental
image assessments, highlighting the potential for AI to improve TPR, TNR, PPV,
and NPV. The studies collectively revealed a statistically significant overall
effect in favor of AI in dental imaging applications. CONCLUSION: In summary,
this systematic review and meta-analysis underscore the transformative impact of
AI on dental imaging. AI has the potential to revolutionize the field by
enhancing accuracy, efficiency, and time savings in various dental tasks. While
further research in clinical settings is needed to validate these findings and
address study limitations, the future implications of integrating AI into dental
practice hold great promise for advancing patient care and the field of
dentistry.
CI - ¬© 2024 The Author(s).
FAU - Alam, Mohammad Khursheed
AU - Alam MK
AD - Preventive Dentistry Department, College of Dentistry, Jouf University, Sakaka,
72345, Saudi Arabia.
AD - Department of Dental Research Cell, Saveetha Institute of Medical and Technical
Sciences, Saveetha Dental College and Hospitals, Chennai, 600077, India.
AD - Department of Public Health, Faculty of Allied Health Sciences, Daffodil
International University, Dhaka, 1207, Bangladesh.
FAU - Alftaikhah, Sultan Abdulkareem Ali
AU - Alftaikhah SAA
AD - Preventive Dentistry Department, College of Dentistry, Jouf University, Sakaka,
72345, Saudi Arabia.
FAU - Issrani, Rakhi
AU - Issrani R
AD - Preventive Dentistry Department, College of Dentistry, Jouf University, Sakaka,
72345, Saudi Arabia.
FAU - Ronsivalle, Vincenzo
AU - Ronsivalle V
AD - Department of Biomedical and Surgical and Biomedical Sciences, Catania
University, 95123, Catania, Italy.
FAU - Lo Giudice, Antonino

AU - Lo Giudice A
AD - Department of Biomedical and Surgical and Biomedical Sciences, Catania
      University, 95123, Catania, Italy.
FAU - Cicci√π, Marco
AU - Cicci√π M
AD - Department of Biomedical and Surgical and Biomedical Sciences, Catania
      University, 95123, Catania, Italy.
FAU - Minervini, Giuseppe
AU - Minervini G
AD - Multidisciplinary Department of Medical-Surgical and Odontostomatological
      Specialties, University of Campania "Luigi Vanvitelli", 80121, Naples, Italy.
AD - Saveetha Dental College and Hospitals, Saveetha Institute of Medical and
      Technical Science (SIMATS), Saveetha University, Chennai, Tamil Nadu, India.
LA - eng
PT - Journal Article
PT - Review
DEP - 20240114
PL - England
TA - Heliyon
JT - Heliyon
JID - 101672560
PMC - PMC10838702
OTO - NOTNLM
OT - Artificial intelligence
OT - Dental imaging
OT - Diagnostic accuracy
OT - In-vitro studies
OT - Meta-analysis
OT - Precision
OT - Systematic review
OT - Time efficiency
COIS- The authors declare that they have no known competing financial interests or
      personal relationships that could have appeared to influence the work reported in
      this paper.
EDAT- 2024/02/06 06:42
MHDA- 2024/02/06 06:43
PMCR- 2024/01/14
CRDT- 2024/02/06 03:49
PHST- 2023/09/30 00:00 [received]
PHST- 2024/01/02 00:00 [revised]
PHST- 2024/01/04 00:00 [accepted]
PHST- 2024/02/06 06:43 [medline]
PHST- 2024/02/06 06:42 [pubmed]
PHST- 2024/02/06 03:49 [entrez]
PHST- 2024/01/14 00:00 [pmc-release]
AID - S2405-8440(24)00252-4 [pii]

AID - e24221 [pii]
AID - 10.1016/j.heliyon.2024.e24221 [doi]
PST - epublish
SO - Heliyon. 2024 Jan 14;10(3):e24221. doi:
10.1016/j.heliyon.2024.e24221.
      eCollection 2024 Feb 15.

PMID- 38289629
OWN - NLM
STAT- MEDLINE
DCOM- 20240131
LR - 20240206
IS - 2158-1797 (Electronic)
IS - 1548-8578 (Linking)
VI - 45
IP - 2
DP - 2024 Feb
TI - Artificial Intelligence: A Mighty Adjunct for Caries Detection.
PG - 110-112
AB - Artificial intelligence (AI) is impacting many aspects of people's
lives today.
      In fields such as finance, manufacturing, agriculture, insurance,
education, and
      healthcare, AI has been commissioned to cut costs, increase
efficiency, and
      improve accuracy. Like many innovations, the use of AI can have
both positive and
      potentially negative consequences. As an example, if used in
dentistry to detect
      interproximal carious lesions, AI technology does not have the
ability (at least
      not yet) to recognize the individual uniqueness of each patient.
For instance,
      some patients are highly caries prone and may be best served by
prophylactic
      intervention of even small borderline lesions, while other patients
are very
      caries resistant and may simply require the monitoring of suspected
carious
      lesions.
FAU - Vakay, Rena
AU - Vakay R
AD - Clinical Instructor, Kois Center, Seattle, Washington; Accredited
Member,
      American Academy of Cosmetic Dentistry; Member, American Academy of
Restorative
      Dentistry; Private Practice, Centreville, Virginia.
FAU - Alex, Gary
AU - Alex G
AD - Accredited Member, American Academy of Cosmetic Dentistry; Member,
International
      Association for Dental Research; Private Practice, Huntington, New
York.
LA - eng

PT - Journal Article
PL - United States
TA - Compend Contin Educ Dent
JT - Compendium of continuing education in dentistry (Jamesburg, N.J. : 1995)
JID - 9600713
MH - Humans
MH - *Artificial Intelligence
MH - Dental Caries Susceptibility
MH - *Dental Caries/diagnosis/prevention & control
EDAT- 2024/01/30 12:43
MHDA- 2024/01/31 06:42
CRDT- 2024/01/30 11:42
PHST- 2024/01/31 06:42 [medline]
PHST- 2024/01/30 12:43 [pubmed]
PHST- 2024/01/30 11:42 [entrez]
PST - ppublish
SO - Compend Contin Educ Dent. 2024 Feb;45(2):110-112.

PMID- 37719493
OWN - NLM
STAT- PubMed-not-MEDLINE
LR - 20230919
IS - 2168-8184 (Print)
IS - 2168-8184 (Electronic)
IS - 2168-8184 (Linking)
VI - 15
IP - 8
DP - 2023 Aug
TI - The Impetus of Artificial Intelligence on Periodontal Diagnosis: A Brief
      Synopsis.
PG - e43583
LID - 10.7759/cureus.43583 [doi]
LID - e43583
AB - The current advances in digitized data additions, machine learning and computing
      framework, lead¬†to the swiftly emerging concept of "Artificial Intelligence"
      (AI), that are developing into areas that were formerly contemplated for human
      expertise.¬†AI is a relatively rapid paced mechanics wherein the computer
      technology is tuned to perform human tasks. An auxiliary domain of AI is machine
      learning (ML), and Deep learning, a subclass of¬†ML technique comprehends
      multi-layer mathematical operations. AI-based applications have tremendous
      potential to improve and systematize patient care thereby alleviating dentists
      from laborious regular tasks, and facilitate personalized, predictive and

preventive dentistry. In the dental clinic, AI can execute a variety of easy tasks with greater accuracy, minimal manpower, and with fewer mistakes over human equivalents. These tasks range from appointment scheduling and coordination to helping with clinical evaluation and therapy. Besides, this could assist in the early diagnosis of dental and maxillofacial abnormalities like periodontal ailments, root caries, bony lesions, and facial malformations in addition to automatically identifying and classifying dental restorations on digital radiographs. This brusque narrative review describes the AI-based systems, their respective applications in periodontal diagnosis, the multifarious studies, possible limitations and the predictable future of AI-based dental diagnostics and treatment planning.
CI - Copyright ¬© 2023, Cholan et al.
FAU - Cholan, Priyanka
AU - Cholan P
AD - Periodontics, Sri Ramaswamy Memorial (SRM) Dental College & Hospital, Chennai, IND.
FAU - Ramachandran, Lakshmi
AU - Ramachandran L
AD - Periodontics & Oral Implantology, Sri Ramaswamy Memorial (SRM) Dental College & Hospital, Chennai, IND.
FAU - Umesh, Santo G
AU - Umesh SG
AD - Periodontics, Sri Ramaswamy Memorial (SRM) Dental College, Chennai, IND.
FAU - P, Sucharitha
AU - P S
AD - Periodontics, Sri Ramaswamy Memorial (SRM) Dental College, Chennai, IND.
FAU - Tadepalli, Anupama
AU - Tadepalli A
AD - Periodontics & Oral Implantology, Sri Ramaswamy Memorial (SRM) Dental College & Hospital, Chennai, IND.
LA - eng
PT - Journal Article
PT - Review
DEP - 20230816
PL - United States
TA - Cureus
JT - Cureus
JID - 101596737
PMC - PMC10503663

OTO - NOTNLM
OT - art of diagnosis
OT - deep learning artificial intelligence
OT - diagnosis
OT - maxillofacial abnormalities
OT - oral health care
OT - periodontal diseases
COIS- The authors have declared that no competing interests exist.
EDAT- 2023/09/18 06:42
MHDA- 2023/09/18 06:43
PMCR- 2023/08/16
CRDT- 2023/09/18 04:22
PHST- 2023/08/16 00:00 [accepted]
PHST- 2023/09/18 06:43 [medline]
PHST- 2023/09/18 06:42 [pubmed]
PHST- 2023/09/18 04:22 [entrez]
PHST- 2023/08/16 00:00 [pmc-release]
AID - 10.7759/cureus.43583 [doi]
PST - epublish
SO - Cureus. 2023 Aug 16;15(8):e43583. doi: 10.7759/cureus.43583. eCollection 2023
      Aug.

PMID- 37665008
OWN - NLM
STAT- MEDLINE
DCOM- 20231002
LR - 20241003
IS - 0250-832X (Print)
IS - 1476-542X (Electronic)
IS - 0250-832X (Linking)
VI - 52
IP - 7
DP - 2023 Oct
TI - Applications of artificial intelligence in the analysis of dental panoramic
      radiographs: an overview of systematic reviews.
PG - 20230284
LID - 10.1259/dmfr.20230284 [doi]
LID - 20230284
AB - OBJECTIVES: This overview of systematic reviews aimed to establish the current
      state of knowledge on the suitability of artificial intelligence (AI) in dental
      panoramic radiograph analysis and illustrate its changes over time. METHODS:
      Medical databases covered by the Association for Computing Machinery, Bielefeld
      Academic Search Engine, Google Scholar, and PubMed engines were searched. The
      risk of bias was assessed using ROBIS tool. Ultimately, 12 articles were
      qualified for the qualitative synthesis. The results were visualized with

timelines, tables, and charts. RESULTS: In the years 1988-2023, a significant development of information technologies for the analysis of DPRs was observed. The latest analyzed AI models achieve high accuracy in detecting caries (91.5%), osteoporosis (89.29%), maxillary sinusitis (87.5%), periodontal bone loss (93.09%), and teeth identification and numbering (93.67%). The detection of periapical lesions is also characterized by high sensitivity (99.95%) and specificity (92%). However, due to the small number of heterogeneous source studies synthesized in systematic reviews, the results of this overview should be interpreted with caution. CONCLUSION: Currently, AI applications can significantly support dentists in dental panoramic radiograph analysis. As systematic reviews on AI become outdated quickly, their regular updating is recommended. PROSPERO registration number: CRD42023416048.
FAU - Turosz, Natalia
AU - Turosz N
AUID- ORCID: 0000-0001-8075-9989
AD - Institute of Public Health, Jagiellonian University Medical College, Skawi≈Ñska, Poland.
FAU - Chƒôci≈Ñska, Kamila
AU - Chƒôci≈Ñska K
AD - Department of Glass Technology and Amorphous Coatings, Faculty of Materials Science and Ceramics, AGH University of Science and Technology, Mickiewicza, Poland.
FAU - Chƒôci≈Ñski, Maciej
AU - Chƒôci≈Ñski M
AD - Department of Oral Surgery, Preventive Medicine Center, Komorowskiego, Poland.
FAU - Brzozowska, Anita
AU - Brzozowska A
AD - Preventive Medicine Center, Komorowskiego, Poland.
FAU - Nowak, Zuzanna
AU - Nowak Z
AD - Department of Temporomandibular Disorders, Medical University of Silesia in Katowice, Katowice, Poland.
FAU - Sikora, Maciej
AU - Sikora M
AD - Department of Maxillofacial Surgery, Hospital of the Ministry of Interior, Wojska Polskiego, Poland.

AD - Department of Biochemistry and Medical Chemistr, Pomeranian Medical University,
      Powsta≈Ñc√≥w Wielkopolskich, Poland.
LA - eng
PT - Journal Article
PT - Review
DEP - 20230904
PL - England
TA - Dentomaxillofac Radiol
JT - Dento maxillo facial radiology
JID - 7609576
MH - Humans
MH - *Alveolar Bone Loss
MH - Artificial Intelligence
MH - *Dental Caries
MH - Radiography, Panoramic
MH - Systematic Reviews as Topic
PMC - PMC10552133
OTO - NOTNLM
OT - Artificial Intelligence
OT - Deep learning
OT - Dental radiography
OT - Overview of reviews
OT - Panoramic radiographs
EDAT- 2023/09/04 12:42
MHDA- 2023/09/26 13:42
PMCR- 2024/10/01
CRDT- 2023/09/04 06:42
PHST- 2023/09/26 13:42 [medline]
PHST- 2023/09/04 12:42 [pubmed]
PHST- 2023/09/04 06:42 [entrez]
PHST- 2024/10/01 00:00 [pmc-release]
AID - 10.1259/dmfr.20230284 [doi]
PST - ppublish
SO - Dentomaxillofac Radiol. 2023 Oct;52(7):20230284. doi: 10.1259/dmfr.20230284. Epub
      2023 Sep 4.

PMID- 37575741
OWN - NLM
STAT- PubMed-not-MEDLINE
LR - 20230815
IS - 2168-8184 (Print)
IS - 2168-8184 (Electronic)
IS - 2168-8184 (Linking)
VI - 15
IP - 7
DP - 2023 Jul
TI - Transforming Dental Caries Diagnosis Through Artificial Intelligence-Based
      Techniques.
PG - e41694
LID - 10.7759/cureus.41694 [doi]
LID - e41694

AB - Diagnosing dental caries plays a pivotal role in preventing and treating tooth decay. However, traditional methods of diagnosing caries often fall short in accuracy and efficiency. Despite the endorsement of radiography as a diagnostic tool, the identification of dental caries through radiographic images can be influenced by individual interpretation. Incorporating artificial intelligence (AI) into diagnosing dental caries holds significant promise, potentially enhancing the precision and efficiency of diagnoses. This review introduces the fundamental concepts of AI, including machine learning and deep learning algorithms, and emphasizes their relevance and potential contributions to the diagnosis of dental caries. It further explains the process of gathering and pre-processing radiography data for AI examination. Additionally, AI techniques for dental caries diagnosis are explored, focusing on image processing, analysis, and classification models for predicting caries risk and severity. Deep learning applications in dental caries diagnosis using convolutional neural networks are presented. Furthermore, the integration of AI systems into dental practice is discussed, including the challenges and considerations for implementation as well as ethical and legal aspects. The breadth of AI technologies and their prospective utility in clinical scenarios for diagnosing dental caries from dental radiographs is presented. This review outlines the advancements of AI and its potential in revolutionizing dental caries diagnosis, encouraging further research and development in this rapidly evolving field.
CI - Copyright ¬© 2023, Anil et al.
FAU - Anil, Sukumaran
AU - Anil S
AD - Dentistry, Hamad Medical Corporation, Doha, QAT.
FAU - Porwal, Priyanka
AU - Porwal P
AD - Dentistry, Pushpagiri Institute of Medical Sciences and Research Centre, Tiruvalla, IND.
FAU - Porwal, Amit
AU - Porwal A
AD - Prosthetic Dental Sciences, College of Dentistry, Jazan University, Jazan, SAU.

LA - eng
PT - Journal Article
PT - Review
DEP - 20230711
PL - United States
TA - Cureus
JT - Cureus
JID - 101596737
PMC - PMC10413921
OTO - NOTNLM
OT - artificial intelligence
OT - clinical applications
OT - convolutional neural networks
OT - data acquisition
OT - dental caries diagnosis
OT - dental radiographs
OT - image analysis
OT - machine learning
OT - performance evaluation
COIS- The authors have declared that no competing interests exist.
EDAT- 2023/08/14 06:42
MHDA- 2023/08/14 06:43
PMCR- 2023/07/11
CRDT- 2023/08/14 04:33
PHST- 2023/07/11 00:00 [accepted]
PHST- 2023/08/14 06:43 [medline]
PHST- 2023/08/14 06:42 [pubmed]
PHST- 2023/08/14 04:33 [entrez]
PHST- 2023/07/11 00:00 [pmc-release]
AID - 10.7759/cureus.41694 [doi]
PST - epublish
SO - Cureus. 2023 Jul 11;15(7):e41694. doi: 10.7759/cureus.41694. eCollection 2023
      Jul.

PMID- 37563659
OWN - NLM
STAT- MEDLINE
DCOM- 20231127
LR - 20240724
IS - 1472-6831 (Electronic)
IS - 1472-6831 (Linking)
VI - 23
IP - 1
DP - 2023 Aug 11
TI - Detection of the pathological exposure of pulp using an artificial intelligence
      tool: a multicentric study over periapical radiographs.
PG - 553
LID - 10.1186/s12903-023-03251-0 [doi]
LID - 553
AB - BACKGROUND: Introducing artificial intelligence (AI) into the medical field

proved beneficial in automating tasks and streamlining the practitioners' lives.
Hence, this study was conducted¬†to design and evaluate an AI tool called¬†Make
Sure Caries Detector and Classifier (MSc)¬†for detecting pathological exposure of
pulp on digital periapical radiographs and to compare its performance with
dentists. METHODS: This study was a diagnostic, multi-centric study, with 3461
digital periapical radiographs from three countries and seven centers. MSc was
built using Yolov5-x model, and it was used for exposed and unexposed pulp
detection. The dataset was split into a train, validate, and test dataset;
the¬†ratio was 8-1-1 to prevent overfitting. 345 images with 752 labels were
randomly allocated to test MSc. The performance metrics used to test MSc
performance included mean average precision (mAP), precision, F1 score, recall,
and area under receiver operating characteristic curve (AUC). The metrics used to
compare the performance with that of 10 certified dentists were: right diagnosis
exposed (RDE), right diagnosis not exposed (RDNE), false diagnosis exposed (FDE),
false diagnosis not exposed (FDNE), missed diagnosis (MD), and over diagnosis
(OD). RESULTS: MSc achieved a performance of more than 90% in all metrics
examined: an average precision of 0.928, recall of 0.918, F1-score of 0.922, and
AUC of 0.956 (P<.05). The results showed a higher mean of 1.94 for all right
(correct) diagnosis parameters in MSc group, while a higher mean of 0.64 for all
wrong diagnosis parameters in the dentists group (P<.05). CONCLUSIONS: The
designed MSc tool proved itself reliable in the detection and differentiating
between exposed and unexposed pulp in the internally validated model. It also
showed a better performance for the detection of exposed and unexposed pulp when
compared to the 10 dentists' consensus.
CI - ¬© 2023. The Author(s).
FAU - Altukroni, A
AU - Altukroni A
AD - Ministry of Health, Medina, Saudi Arabia. research-manager@swc-dent.com.
FAU - Alsaeedi, A
AU - Alsaeedi A

AD - Department of Computer Science, College of Computer Science and Engineering,
      Taibah University, Medina, Saudi Arabia.
FAU - Gonzalez-Losada, C
AU - Gonzalez-Losada C
AD - School of Dentistry, Complutense University of Madrid, Madrid, Spain.
FAU - Lee, J H
AU - Lee JH
AD - Department of Periodontology, College of Dentistry and Institute of Oral
      Bioscience, Jeonbuk National University, Jeonju, Korea.
FAU - Alabudh, M
AU - Alabudh M
AD - Ministry of Health, Medina, Saudi Arabia.
FAU - Mirah, M
AU - Mirah M
AD - Department of Dental Materials, Taibah University, Medina, Saudi Arabia.
FAU - El-Amri, S
AU - El-Amri S
AD - Ministry of Health, Ankara, Turkey.
FAU - Ezz El-Deen, O
AU - Ezz El-Deen O
AD - Ministry of Health, Cairo, Egypt.
LA - eng
PT - Journal Article
PT - Multicenter Study
PT - Research Support, Non-U.S. Gov't
DEP - 20230811
PL - England
TA - BMC Oral Health
JT - BMC oral health
JID - 101088684
SB - IM
MH - Humans
MH - *Artificial Intelligence
MH - *Dental Pulp/diagnostic imaging
MH - ROC Curve
MH - Radiography
PMC - PMC10416487
OTO - NOTNLM
OT - Artificial Intelligence
OT - Caries
OT - Deep Learning
OT - Periapical Radiograph
OT - Pulp Exposure
OT - Yolov5x
COIS- The authors declare no competing interests.
EDAT- 2023/08/11 00:42
MHDA- 2023/11/27 12:42
PMCR- 2023/08/11
CRDT- 2023/08/10 23:46
PHST- 2023/02/26 00:00 [received]

PHST- 2023/07/25 00:00 [accepted]
PHST- 2023/11/27 12:42 [medline]
PHST- 2023/08/11 00:42 [pubmed]
PHST- 2023/08/10 23:46 [entrez]
PHST- 2023/08/11 00:00 [pmc-release]
AID - 10.1186/s12903-023-03251-0 [pii]
AID - 3251 [pii]
AID - 10.1186/s12903-023-03251-0 [doi]
PST - epublish
SO - BMC Oral Health. 2023 Aug 11;23(1):553. doi: 10.1186/s12903-023-03251-0.

PMID- 37392423
OWN - NLM
STAT- MEDLINE
DCOM- 20240531
LR - 20250626
IS - 1601-0825 (Electronic)
IS - 1354-523X (Linking)
VI - 30
IP - 4
DP - 2024 May
TI - Detecting dental caries on oral photographs using artificial intelligence: A
      systematic review.
PG - 1765-1783
LID - 10.1111/odi.14659 [doi]
AB - OBJECTIVES: This systematic review aimed at evaluating the performance of
      artificial intelligence (AI) models in detecting dental caries on oral
      photographs. METHODS: Methodological characteristics and performance metrics of
      clinical studies reporting on deep learning and other machine learning algorithms
      were assessed. The risk of bias was evaluated using the quality assessment of
      diagnostic accuracy studies 2 (QUADAS-2) tool. A systematic search was conducted
      in EMBASE, Medline, and Scopus. RESULTS: Out of 3410 identified records, 19
      studies were included with six and seven studies having low risk of biases and
      applicability concerns for all the domains, respectively. Metrics varied widely
      and were assessed on multiple levels. F1-scores for classification and detection
      tasks were 68.3%-94.3% and 42.8%-95.4%, respectively. Irrespective of the task,
      F1-scores were 68.3%-95.4% for professional cameras, 78.8%-87.6%, for intraoral
      cameras, and 42.8%-80% for smartphone cameras. Limited studies allowed assessing

AI performance for lesions of different severity. CONCLUSION: Automatic detection
of dental caries using AI may provide objective verification of clinicians'
diagnoses and facilitate patient-clinician communication and teledentistry.
Future studies should consider more robust study designs, employ comparable and
standardized metrics, and focus on the severity of caries lesions.
CI - ¬© 2023 The Authors. Oral Diseases published by Wiley Periodicals LLC.
FAU - Moharrami, Mohammad
AU - Moharrami M
AUID- ORCID: 0000-0002-4364-7969
AD - Faculty of Dentistry, University of Toronto, Toronto, Ontario, Canada.
AD - Topic Group Dental Diagnostics and Digital Dentistry, ITU/WHO Focus Group AI on
Health, Geneva, Switzerland.
FAU - Farmer, Julie
AU - Farmer J
AD - Faculty of Dentistry, University of Toronto, Toronto, Ontario, Canada.
FAU - Singhal, Sonica
AU - Singhal S
AD - Faculty of Dentistry, University of Toronto, Toronto, Ontario, Canada.
AD - Health Promotion, Chronic Disease and Injury Prevention Department, Public Health
Ontario, Toronto, Canada.
FAU - Watson, Erin
AU - Watson E
AUID- ORCID: 0000-0002-2096-7732
AD - Faculty of Dentistry, University of Toronto, Toronto, Ontario, Canada.
AD - Department of Dental Oncology, Princess Margaret Cancer Centre, Toronto, Ontario,
Canada.
FAU - Glogauer, Michael
AU - Glogauer M
AD - Faculty of Dentistry, University of Toronto, Toronto, Ontario, Canada.
AD - Department of Dental Oncology, Princess Margaret Cancer Centre, Toronto, Ontario,
Canada.
AD - Department of Dentistry, Centre for Advanced Dental Research and Care, Mount
Sinai Hospital, Toronto, Ontario, Canada.
FAU - Johnson, Alistair E W
AU - Johnson AEW
AUID- ORCID: 0000-0002-8735-3014
AD - Program in Child Health Evaluative Sciences, The Hospital for Sick Children,
Toronto, Ontario, Canada.

FAU - Schwendicke, Falk
AU - Schwendicke F
AD - Topic Group Dental Diagnostics and Digital Dentistry, ITU/WHO Focus Group AI on
      Health, Geneva, Switzerland.
AD - Oral Diagnostics, Digital Health and Health Services Research, Charit√©¬†-
      Universit√§tsmedizin Berlin, Berlin, Germany.
FAU - Quinonez, Carlos
AU - Quinonez C
AD - Faculty of Dentistry, University of Toronto, Toronto, Ontario, Canada.
AD - Schulich School of Medicine and Dentistry, Western University, London, Ontario,
      Canada.
LA - eng
GR - 001/WHO_/World Health Organization/International
PT - Journal Article
PT - Research Support, Non-U.S. Gov't
PT - Systematic Review
DEP - 20230701
PL - Denmark
TA - Oral Dis
JT - Oral diseases
JID - 9508565
SB - IM
CIN - Oral Dis. 2024 Jul;30(5):3549-3550. doi: 10.1111/odi.14794. PMID: 37890040
MH - Humans
MH - *Artificial Intelligence
MH - Deep Learning
MH - *Dental Caries/diagnosis
MH - Machine Learning
MH - Photography
MH - Photography, Dental
MH - Image Processing, Computer-Assisted
OTO - NOTNLM
OT - deep learning
OT - dental caries
OT - intraoral camera
OT - oral photograph
OT - smartphone
EDAT- 2023/07/01 21:09
MHDA- 2024/05/31 12:42
CRDT- 2023/07/01 14:43
PHST- 2023/05/19 00:00 [revised]
PHST- 2023/03/21 00:00 [received]
PHST- 2023/06/15 00:00 [accepted]
PHST- 2024/05/31 12:42 [medline]
PHST- 2023/07/01 21:09 [pubmed]
PHST- 2023/07/01 14:43 [entrez]
AID - 10.1111/odi.14659 [doi]
PST - ppublish

SO - Oral Dis. 2024 May;30(4):1765-1783. doi: 10.1111/odi.14659. Epub 2023 Jul 1.

PMID- 36381137
OWN - NLM
STAT- PubMed-not-MEDLINE
LR - 20240912
IS - 2767-3170 (Electronic)
IS - 2767-3170 (Linking)
VI - 1
IP - 6
DP - 2022
TI - Artificial intelligence-powered smartphone application, AICaries, improves
      at-home dental caries screening in children: Moderated and unmoderated usability
      test.
LID - e0000046 [pii]
LID - 10.1371/journal.pdig.0000046 [doi]
AB - Early Childhood Caries (ECC) is the most common childhood disease worldwide and a
      health disparity among underserved children. ECC is preventable and reversible if
      detected early. However, many children from low-income families encounter
      barriers to dental care. An at-home caries detection technology could potentially
      improve access to dental care regardless of patients' economic status and address
      the overwhelming prevalence of ECC. Our team has developed a smartphone
      application (app), AICaries, that uses artificial intelligence (AI)-powered
      technology to detect caries using children's teeth photos. We used mixed methods
      to assess the acceptance, usability, and feasibility of the AICaries app among
      underserved parent-child dyads. We conducted moderated usability testing (Step 1)
      with ten parent-child dyads using "Think-aloud" methods to assess the flow and
      functionality of the app and analyze the data to refine the app and procedures.
      Next, we conducted unmoderated field testing (Step 2) with 32 parent-child dyads
      to test the app within their natural environment (home) over two weeks. We
      administered the System Usability Scale (SUS) and conducted semi-structured
      individual interviews with parents and conducted thematic analyses. AICaries app
      received a 78.4 SUS score from the participants, indicating an excellent

acceptance. Notably, the majority (78.5%) of parent-taken photos of children's
teeth were satisfactory in quality for detection of caries using the AI app.
Parents suggested using community health workers to provide training to parents
needing assistance in taking high quality photos of their young child's teeth.
Perceived benefits from using the AICaries app include convenient at-home caries
screening, informative on caries risk and education, and engaging family members.
Data from this study support future clinical trial that evaluates the real-world
impact of using this innovative smartphone app on early detection and prevention
of ECC among low-income children.
FAU - Al-Jallad, Nisreen
AU - Al-Jallad N
AUID- ORCID: 0000-0002-5990-8123
AD - Eastman Institute for Oral Health, University of Rochester Medical Center,
Rochester, NY, United States of America.
FAU - Ly-Mapes, Oriana
AU - Ly-Mapes O
AUID- ORCID: 0000-0002-0859-921X
AD - Eastman Institute for Oral Health, University of Rochester Medical Center,
Rochester, NY, United States of America.
FAU - Hao, Peirong
AU - Hao P
AUID- ORCID: 0000-0002-2853-1562
AD - Department of Computer Science, University of Rochester, United States of
America.
FAU - Ruan, Jinlong
AU - Ruan J
AUID- ORCID: 0000-0003-0207-5463
AD - Department of Computer Science, University of Rochester, United States of
America.
FAU - Ramesh, Ashwin
AU - Ramesh A
AUID- ORCID: 0000-0002-7481-538X
AD - Department of Computer Science, University of Rochester, United States of
America.
FAU - Luo, Jiebo
AU - Luo J
AUID- ORCID: 0000-0002-4516-9729
AD - Department of Computer Science, University of Rochester, United States of
America.
FAU - Wu, Tong Tong

AU - Wu TT
AUID- ORCID: 0000-0002-1175-9923
AD - Department of Biostatistics and computational biology, University of Rochester
      Medical Center, Rochester, United States of America.
FAU - Dye, Timothy
AU - Dye T
AUID- ORCID: 0000-0002-9801-4712
AD - Department of Obstetrics and Gynecology, University of Rochester Medical Center,
      Rochester, United States of America.
FAU - Rashwan, Noha
AU - Rashwan N
AUID- ORCID: 0000-0001-7604-4227
AD - Eastman Institute for Oral Health, University of Rochester Medical Center,
      Rochester, NY, United States of America.
FAU - Ren, Johana
AU - Ren J
AD - University of Rochester, United States of America.
FAU - Jang, Hoonji
AU - Jang H
AD - Temple University School of Dentistry, Pennsylvania, United States of America.
FAU - Mendez, Luis
AU - Mendez L
AUID- ORCID: 0000-0002-8302-0474
AD - Eastman Institute for Oral Health, University of Rochester Medical Center,
      Rochester, NY, United States of America.
FAU - Alomeir, Nora
AU - Alomeir N
AUID- ORCID: 0000-0002-6893-3697
AD - Eastman Institute for Oral Health, University of Rochester Medical Center,
      Rochester, NY, United States of America.
FAU - Bullock, Sherita
AU - Bullock S
AUID- ORCID: 0000-0002-3727-8220
AD - Healthy Baby Network, Rochester, United States of America.
FAU - Fiscella, Kevin
AU - Fiscella K
AUID- ORCID: 0000-0003-3613-8012
AD - Department of Family Medicine, University of Rochester Medical Center, Rochester,
      NY, United States of America.
FAU - Xiao, Jin
AU - Xiao J
AUID- ORCID: 0000-0002-8776-2520
AD - Eastman Institute for Oral Health, University of Rochester Medical Center,
      Rochester, NY, United States of America.
LA - eng
GR - K23 DE027412/DE/NIDCR NIH HHS/United States

GR - R21 DE030251/DE/NIDCR NIH HHS/United States
PT - Journal Article
DEP - 20220602
PL - United States
TA - PLOS Digit Health
JT - PLOS digital health
JID - 9918335064206676
PMC - PMC9645586
MID - NIHMS1843040
COIS- Competing interests: The authors have declared that no competing interests exist.
EDAT- 2022/11/17 06:00
MHDA- 2022/11/17 06:01
PMCR- 2022/06/02
CRDT- 2022/11/16 02:48
PHST- 2022/11/16 02:48 [entrez]
PHST- 2022/11/17 06:00 [pubmed]
PHST- 2022/11/17 06:01 [medline]
PHST- 2022/06/02 00:00 [pmc-release]
AID - e0000046 [pii]
AID - PDIG-D-22-00066 [pii]
AID - 10.1371/journal.pdig.0000046 [doi]
PST - ppublish
SO - PLOS Digit Health. 2022;1(6):e0000046. doi: 10.1371/journal.pdig.0000046. Epub 2022 Jun 2.

PMID- 35996332
OWN - NLM
STAT- MEDLINE
DCOM- 20220928
LR - 20221101
IS - 1544-0591 (Electronic)
IS - 0022-0345 (Print)
IS - 0022-0345 (Linking)
VI - 101
IP - 11
DP - 2022 Oct
TI - Artificial Intelligence for Caries Detection: Value of Data and Information.
PG - 1350-1356
LID - 10.1177/00220345221113756 [doi]
AB - If increasing practitioners' diagnostic accuracy, medical artificial intelligence (AI) may lead to better treatment decisions at lower costs, while uncertainty remains around the resulting cost-effectiveness. In the present study, we assessed how enlarging the data set used for training an AI for caries detection on bitewings affects cost-effectiveness and also determined the value of information by reducing the uncertainty around other input parameters (namely,

the costs of AI and the population's caries risk profile). We employed a
convolutional neural network and trained it on 10%, 25%, 50%, or 100% of a
labeled data set containing 29,011 teeth without and 19,760 teeth with caries
lesions stemming from bitewing radiographs. We employed an established health
economic modeling and analytical framework to quantify cost-effectiveness and
value of information. We adopted a mixed public-private payer perspective in
German health care; the health outcome was tooth retention years. A Markov model,
allowing to follow posterior teeth over the lifetime of an initially 12-y-old
individual, and Monte Carlo microsimulations were employed. With an increasing
amount of data used to train the AI sensitivity and specificity increased
nonlinearly, increasing the data set from 10% to 25% had the largest impact on
accuracy and, consequently, cost-effectiveness. In the base-case scenario, AI was
more effective (tooth retention for a mean [2.5%-97.5%] 62.8 [59.2-65.5] y) and
less costly (378 [284-499] euros) than dentists without AI (60.4 [55.8-64.4] y;
419 [270-593] euros), with considerable uncertainty. The economic value of
reducing the uncertainty around AI's accuracy or costs was limited, while
information on the population's risk profile was more relevant. When developing
dental AI, informed choices about the data set size may be recommended, and
research toward individualized application of AI for caries detection seems
warranted to optimize cost-effectiveness.
FAU - Schwendicke, F
AU - Schwendicke F
AUID- ORCID: 0000-0003-1223-1669
AD - Department of Oral Diagnostics, Digital Health and Health Services Research,
Charit√©-Universit√§tsmedizin Berlin, Berlin, Germany.
FAU - Cejudo Grano de Oro, J
AU - Cejudo Grano de Oro J
AD - Department of Oral Diagnostics, Digital Health and Health Services Research,
Charit√©-Universit√§tsmedizin Berlin, Berlin, Germany.
FAU - Garcia Cantu, A
AU - Garcia Cantu A
AD - Department of Oral Diagnostics, Digital Health and Health Services Research,

      Charit√©-Universit√§tsmedizin Berlin, Berlin, Germany.
FAU - Meyer-Lueckel, H
AU - Meyer-Lueckel H
AD - Department of Restorative, Preventive and Pediatric Dentistry, zmk bern,
      University of Bern, Bern, Switzerland.
FAU - Chaurasia, A
AU - Chaurasia A
AD - Department of Oral Medicine and Radiology, King George's Medical University,
      Lucknow, India.
FAU - Krois, J
AU - Krois J
AUID- ORCID: 0000-0002-6010-8940
AD - Department of Oral Diagnostics, Digital Health and Health Services Research,
      Charit√©-Universit√§tsmedizin Berlin, Berlin, Germany.
LA - eng
PT - Journal Article
DEP - 20220822
PL - United States
TA - J Dent Res
JT - Journal of dental research
JID - 0354343
SB - IM
MH - Artificial Intelligence
MH - Cost-Benefit Analysis
MH - *Dental Caries/diagnostic imaging
MH - *Dental Caries Susceptibility
MH - Humans
MH - Monte Carlo Method
PMC - PMC9516598
OTO - NOTNLM
OT - AI
OT - caries detection/diagnosis/prevention
OT - computer simulation
OT - dental informatics
OT - economic evaluation
OT - radiology
COIS- Declaration of Conflicting Interests: The authors declared the following
      potential conflicts of interest with respect to the research, authorship, and/or
      publication of this article: F. Schwendicke and J. Krois are cofounders of a
      Charit√© startup on dental image analysis, dentalXrai Ltd. The conduct, analysis,
      and interpretation of this study and its findings were unrelated to this.
EDAT- 2022/08/24 06:00
MHDA- 2022/09/28 06:00
PMCR- 2022/09/28
CRDT- 2022/08/23 01:13
PHST- 2022/08/24 06:00 [pubmed]

PHST- 2022/09/28 06:00 [medline]
PHST- 2022/08/23 01:13 [entrez]
PHST- 2022/09/28 00:00 [pmc-release]
AID - 10.1177_00220345221113756 [pii]
AID - 10.1177/00220345221113756 [doi]
PST - ppublish
SO - J Dent Res. 2022 Oct;101(11):1350-1356. doi:
10.1177/00220345221113756. Epub 2022
      Aug 22.

PMID- 35626239
OWN - NLM
STAT- PubMed-not-MEDLINE
LR - 20220716
IS - 2075-4418 (Print)
IS - 2075-4418 (Electronic)
IS - 2075-4418 (Linking)
VI - 12
IP - 5
DP - 2022 Apr 26
TI - Application and Performance of Artificial Intelligence Technology in Detection,
      Diagnosis and Prediction of Dental Caries (DC)-A Systematic Review.
LID - 10.3390/diagnostics12051083 [doi]
LID - 1083
AB - Evolution in the fields of science and technology has led to the development of
      newer applications based on Artificial Intelligence (AI) technology that have
      been widely used in medical sciences. AI-technology has been employed in a wide
      range of applications related to the diagnosis of oral diseases that have
      demonstrated phenomenal precision and accuracy in their performance. The aim of
      this systematic review is to report on the diagnostic accuracy and performance of
      AI-based models designed for detection, diagnosis, and prediction of dental
      caries (DC). Eminent electronic databases (PubMed, Google scholar, Scopus, Web of
      science, Embase, Cochrane, Saudi Digital Library) were searched for relevant
      articles that were published from January 2000 until February 2022. A total of 34
      articles that met the selection criteria were critically analyzed based on
      QUADAS-2 guidelines. The certainty of the evidence of the included studies was
      assessed using the GRADE approach. AI has been widely applied for prediction of
      DC, for detection and diagnosis of DC and for classification of DC. These models

have demonstrated excellent performance and can be used in clinical practice for
enhancing the diagnostic performance, treatment quality and patient outcome and
can also be applied to identify patients with a higher risk of developing DC.
FAU - Khanagar, Sanjeev B
AU - Khanagar SB
AUID- ORCID: 0000-0002-4098-7773
AD - Preventive Dental Science Department, College of Dentistry, King Saud bin
Abdulaziz University for Health Sciences, Riyadh 11426, Saudi Arabia.
AD - King Abdullah International Medical Research Centre, Ministry of National Guard
Health Affairs, Riyadh 11481, Saudi Arabia.
FAU - Alfouzan, Khalid
AU - Alfouzan K
AD - King Abdullah International Medical Research Centre, Ministry of National Guard
Health Affairs, Riyadh 11481, Saudi Arabia.
AD - Restorative and Prosthetic Dental Sciences Department, College of Dentistry, King
Saud bin Abdulaziz University for Health Sciences, Riyadh 11426, Saudi Arabia.
FAU - Awawdeh, Mohammed
AU - Awawdeh M
AD - Preventive Dental Science Department, College of Dentistry, King Saud bin
Abdulaziz University for Health Sciences, Riyadh 11426, Saudi Arabia.
AD - King Abdullah International Medical Research Centre, Ministry of National Guard
Health Affairs, Riyadh 11481, Saudi Arabia.
FAU - Alkadi, Lubna
AU - Alkadi L
AUID- ORCID: 0000-0003-3206-8547
AD - King Abdullah International Medical Research Centre, Ministry of National Guard
Health Affairs, Riyadh 11481, Saudi Arabia.
AD - Restorative and Prosthetic Dental Sciences Department, College of Dentistry, King
Saud bin Abdulaziz University for Health Sciences, Riyadh 11426, Saudi Arabia.
FAU - Albalawi, Farraj
AU - Albalawi F
AD - Preventive Dental Science Department, College of Dentistry, King Saud bin
Abdulaziz University for Health Sciences, Riyadh 11426, Saudi Arabia.
AD - King Abdullah International Medical Research Centre, Ministry of National Guard
Health Affairs, Riyadh 11481, Saudi Arabia.
FAU - Alfadley, Abdulmohsen

AU - Alfadley A
AUID- ORCID: 0000-0002-5868-034X
AD - King Abdullah International Medical Research Centre, Ministry of National Guard
      Health Affairs, Riyadh 11481, Saudi Arabia.
AD - Restorative and Prosthetic Dental Sciences Department, College of Dentistry, King
      Saud bin Abdulaziz University for Health Sciences, Riyadh 11426, Saudi Arabia.
LA - eng
PT - Journal Article
PT - Review
DEP - 20220426
PL - Switzerland
TA - Diagnostics (Basel)
JT - Diagnostics (Basel, Switzerland)
JID - 101658402
PMC - PMC9139989
OTO - NOTNLM
OT - artificial intelligence
OT - dental caries
OT - detection
OT - diagnosis
OT - prediction
COIS- The authors declare no conflict of interest.
EDAT- 2022/05/29 06:00
MHDA- 2022/05/29 06:01
PMCR- 2022/04/26
CRDT- 2022/05/28 01:10
PHST- 2022/03/20 00:00 [received]
PHST- 2022/04/12 00:00 [revised]
PHST- 2022/04/25 00:00 [accepted]
PHST- 2022/05/28 01:10 [entrez]
PHST- 2022/05/29 06:00 [pubmed]
PHST- 2022/05/29 06:01 [medline]
PHST- 2022/04/26 00:00 [pmc-release]
AID - diagnostics12051083 [pii]
AID - diagnostics-12-01083 [pii]
AID - 10.3390/diagnostics12051083 [doi]
PST - epublish
SO - Diagnostics (Basel). 2022 Apr 26;12(5):1083. doi: 10.3390/diagnostics12051083.

PMID- 35626185
OWN - NLM
STAT- PubMed-not-MEDLINE
LR - 20220716
IS - 2075-4418 (Print)
IS - 2075-4418 (Electronic)
IS - 2075-4418 (Linking)
VI - 12
IP - 5
DP - 2022 Apr 19

TI - Artificial Intelligence in the Diagnosis of Oral Diseases: Applications and
      Pitfalls.
LID - 10.3390/diagnostics12051029 [doi]
LID - 1029
AB - Background: Machine learning (ML) is a key component of artificial intelligence
      (AI). The terms machine learning, artificial intelligence, and deep learning are
      erroneously used interchangeably as they appear as monolithic nebulous entities.
      This technology offers immense possibilities and opportunities to advance
      diagnostics in the field of medicine and dentistry. This necessitates a deep
      understanding of AI and its essential components, such as machine learning (ML),
      artificial neural networks (ANN), and deep learning (DP). Aim: This review aims
      to enlighten clinicians regarding AI and its applications in the diagnosis of
      oral diseases, along with the prospects and challenges involved. Review results:
      AI has been used in the diagnosis of various oral diseases, such as dental
      caries, maxillary sinus diseases, periodontal diseases, salivary gland diseases,
      TMJ disorders, and oral cancer through clinical data and diagnostic images.
      Larger data sets would enable AI to predict the occurrence of precancerous
      conditions. They can aid in population-wide surveillance and decide on referrals
      to specialists. AI can efficiently detect microfeatures beyond the human eye and
      augment its predictive power in critical diagnosis. Conclusion: Although studies
      have recognized the benefit of AI, the use of artificial intelligence and machine
      learning has not been integrated into routine dentistry. AI is still in the
      research phase. The coming decade will see immense changes in diagnosis and
      healthcare built on the back of this research. Clinical significance: This paper
      reviews the various applications of AI in dentistry and illuminates the
      shortcomings faced while dealing with AI research and suggests ways to tackle
      them. Overcoming these pitfalls will aid in integrating AI seamlessly into
      dentistry.
FAU - Patil, Shankargouda
AU - Patil S

AUID- ORCID: 0000-0001-7246-5497
AD - Department of Maxillofacial Surgery and Diagnostic Sciences, Division of Oral
      Pathology, College of Dentistry, Jazan University, Jazan 45142, Saudi Arabia.
FAU - Albogami, Sarah
AU - Albogami S
AUID- ORCID: 0000-0003-0774-5550
AD - Department of Biotechnology, College of Science, Taif University, Taif 21944,
      Saudi Arabia.
FAU - Hosmani, Jagadish
AU - Hosmani J
AUID- ORCID: 0000-0003-3579-0906
AD - Department of Diagnostic Dental Sciences, Oral Pathology Division, Faculty of
      Dentistry, College of Dentistry, King Khalid University, Abha 61411, Saudi
      Arabia.
FAU - Mujoo, Sheetal
AU - Mujoo S
AD - Division of Oral Medicine & Radiology, College of Dentistry, Jazan University,
      Jazan 45142, Saudi Arabia.
FAU - Kamil, Mona Awad
AU - Kamil MA
AD - Department of Preventive Dental Science, College of Dentistry, Jazan University,
      Jazan 45142, Saudi Arabia.
FAU - Mansour, Manawar Ahmad
AU - Mansour MA
AD - Department of Prosthetic Dental Sciences, College of Dentistry, Jazan University,
      Jazan 45142, Saudi Arabia.
FAU - Abdul, Hina Naim
AU - Abdul HN
AD - Department of Prosthetic Dental Sciences, College of Dentistry, Jazan University,
      Jazan 45142, Saudi Arabia.
FAU - Bhandi, Shilpa
AU - Bhandi S
AD - Department of Restorative Dental Sciences, Division of Operative Dentistry,
      College of Dentistry, Jazan University, Jazan 45142, Saudi Arabia.
FAU - Ahmed, Shiek S S J
AU - Ahmed SSSJ
AD - Multi-Omics and Drug Discovery Lab, Chettinad Academy of Research and Education,
      Chennai 600130, India.
LA - eng
PT - Journal Article
PT - Review
DEP - 20220419
PL - Switzerland

TA - Diagnostics (Basel)
JT - Diagnostics (Basel, Switzerland)
JID - 101658402
PMC - PMC9139975
OTO - NOTNLM
OT - artificial intelligence
OT - artificial neural network
OT - deep learning
OT - diagnosis
OT - machine learning
OT - oral diseases
COIS- The authors declare no conflict of interest.
EDAT- 2022/05/29 06:00
MHDA- 2022/05/29 06:01
PMCR- 2022/04/19
CRDT- 2022/05/28 01:10
PHST- 2022/03/29 00:00 [received]
PHST- 2022/04/12 00:00 [revised]
PHST- 2022/04/18 00:00 [accepted]
PHST- 2022/05/28 01:10 [entrez]
PHST- 2022/05/29 06:00 [pubmed]
PHST- 2022/05/29 06:01 [medline]
PHST- 2022/04/19 00:00 [pmc-release]
AID - diagnostics12051029 [pii]
AID - diagnostics-12-01029 [pii]
AID - 10.3390/diagnostics12051029 [doi]
PST - epublish
SO - Diagnostics (Basel). 2022 Apr 19;12(5):1029. doi:
10.3390/diagnostics12051029.

PMID- 35502299
OWN - NLM
STAT- PubMed-not-MEDLINE
LR - 20220716
IS - 2090-1941 (Print)
IS - 2090-195X (Electronic)
IS - 2090-195X (Linking)
VI - 2022
DP - 2022
TI - Oral Cancer Screening by Artificial Intelligence-Oriented
Interpretation of
      Optical Coherence Tomography Images.
PG - 1614838
LID - 10.1155/2022/1614838 [doi]
LID - 1614838
AB - Early diagnosis of oral cancer is critical to improve the survival
rate of
      patients. The current strategies for screening of patients for oral
premalignant
      and malignant lesions unfortunately miss a significant number of
involved
      patients. Optical coherence tomography (OCT) is an optical imaging
modality that

      has been widely investigated in the field of oncology for identification of
      cancerous entities. Since the interpretation of OCT images requires professional
      training and OCT images contain information that cannot be inferred visually,
      artificial intelligence (AI) with trained algorithms has the ability to quantify
      visually undetectable variations, thus overcoming the barriers that have
      postponed the involvement of OCT in the process of screening of oral neoplastic
      lesions. This literature review aimed to highlight the features of precancerous
      and cancerous oral lesions on OCT images and specify how AI can assist in
      screening and diagnosis of such pathologies.
CI - Copyright ¬© 2022 Kousar Ramezani and Maryam Tofangchiha.
FAU - Ramezani, Kousar
AU - Ramezani K
AUID- ORCID: 0000-0001-5416-5271
AD - Department of Oral and Maxillofacial Radiology, Dental Caries Prevention Research
      Center, Qazvin University of Medical Sciences, Qazvin, Iran.
FAU - Tofangchiha, Maryam
AU - Tofangchiha M
AUID- ORCID: 0000-0002-5515-2189
AD - Department of Oral and Maxillofacial Radiology, Dental Caries Prevention Research
      Center, Qazvin University of Medical Sciences, Qazvin, Iran.
LA - eng
PT - Journal Article
PT - Review
DEP - 20220423
PL - United States
TA - Radiol Res Pract
JT - Radiology research and practice
JID - 101566860
PMC - PMC9056242
COIS- The authors declare that they have no conflicts of interest.
EDAT- 2022/05/04 06:00
MHDA- 2022/05/04 06:01
PMCR- 2022/04/23
CRDT- 2022/05/03 02:08
PHST- 2021/11/26 00:00 [received]
PHST- 2022/03/23 00:00 [revised]
PHST- 2022/04/11 00:00 [accepted]
PHST- 2022/05/03 02:08 [entrez]
PHST- 2022/05/04 06:00 [pubmed]
PHST- 2022/05/04 06:01 [medline]
PHST- 2022/04/23 00:00 [pmc-release]
AID - 10.1155/2022/1614838 [doi]
PST - epublish

SO - Radiol Res Pract. 2022 Apr 23;2022:1614838. doi:
10.1155/2022/1614838.
      eCollection 2022.

PMID- 35245626
OWN - NLM
STAT- MEDLINE
DCOM- 20220422
LR - 20220422
IS - 1879-176X (Electronic)
IS - 0300-5712 (Linking)
VI - 119
DP - 2022 Apr
TI - Cost-effectiveness of AI for caries detection: randomized trial.
PG - 104080
LID - S0300-5712(22)00137-3 [pii]
LID - 10.1016/j.jdent.2022.104080 [doi]
AB - OBJECTIVES: We assessed the cost-effectiveness of AI-supported detection of proximal caries in a randomized controlled clustered cross-over superiority trial. METHODS: Twenty-three dentists were sampled to assess 20 bitewings; 10 were randomly evaluated supported by an AI-based software (dentalXrai¬†Pro 1.0.4, dentalXrai Ltd, Berlin, Germany) and the other 10 without AI support. The reference test had been established by four independent experts and an additional review. We evaluated the proportion of true and false positive and negative detections and the treatment decisions assigned to each detection (non-invasive, micro-invasive, invasive). Cost-effectiveness was assessed using a mixed public-private-payer perspective in German healthcare. Using the accuracy and treatment decision data from the trial, a Markov simulation model was populated and posterior permanent teeth in initially 31-years old individuals followed over their lifetime. The model allowed extrapolation from the initial detection and therapy to treatment success, re-treatments and, eventually, tooth loss and replacement, capturing long-term effectiveness (tooth retention) and costs (cumulative in Euro). Costs were estimated using the German public and private fee catalogues. Monte-Carlo microsimulations were used and incremental cost-effectiveness at different willingness-to-pay ceiling thresholds assessed.

RESULTS: In the trial, AI-supported detection was significantly more sensitive
than detection without AI. However, in the AI group, lesions were more often
treated invasively. As a result, AI and no AI showed identical effectiveness
(tooth retention for a mean (2.5-97.5%) 49 (48-51) years) and nearly identical
costs (AI: 330 (250-409) Euro, no AI: 330 (248-410) Euro). 41% simulations found
AI and 43% no AI to be more cost-effective. The resulting cost-effectiveness
remained uncertain regardless of a payer's willingness-to-pay. CONCLUSIONS:
Higher accuracy of AI did not lead to higher cost-effectiveness, as more invasive
treatment approaches generated costs and diminished possible effectiveness
advantages. CLINICAL SIGNIFICANCE: The cost-effectiveness of AI could be improved
by supporting not only caries detection, but also subsequent management.
CI - Copyright ¬© 2022. Published by Elsevier Ltd.
FAU - Schwendicke, Falk
AU - Schwendicke F
AD - Department of Oral Diagnostics, Digital Health and Health Services Research,
Charit√© - Universit√§tsmedizin Berlin, Germany. Electronic address:
falk.schwendicke@charite.de.
FAU - Mertens, Sarah
AU - Mertens S
AD - Department of Oral Diagnostics, Digital Health and Health Services Research,
Charit√© - Universit√§tsmedizin Berlin, Germany.
FAU - Cantu, Anselmo Garcia
AU - Cantu AG
AD - Department of Oral Diagnostics, Digital Health and Health Services Research,
Charit√© - Universit√§tsmedizin Berlin, Germany.
FAU - Chaurasia, Akhilanand
AU - Chaurasia A
AD - Faculty of Dental Sciences, King George's Medical University,India.
FAU - Meyer-Lueckel, Hendrik
AU - Meyer-Lueckel H
AD - Operative, Preventive and Paediatric Dentistry, zmk Bern, Switzerland.
FAU - Krois, Joachim
AU - Krois J
AD - Department of Oral Diagnostics, Digital Health and Health Services Research,
Charit√© - Universit√§tsmedizin Berlin, Germany.
LA - eng
PT - Journal Article

PT - Randomized Controlled Trial
DEP - 20220301
PL - England
TA - J Dent
JT - Journal of dentistry
JID - 0354422
SB - IM
MH - Adult
MH - Artificial Intelligence
MH - Computer Simulation
MH - Cost-Benefit Analysis
MH - *Dental Caries/diagnosis/therapy
MH - *Dental Caries Susceptibility
MH - Humans
OTO - NOTNLM
OT - Artificial Intelligence
OT - Caries detection/diagnosis/prevention
OT - Computer Simulation
OT - Decision-Making
OT - Dental
OT - Economic Evaluation
OT - Radiology
EDAT- 2022/03/05 06:00
MHDA- 2022/04/23 06:00
CRDT- 2022/03/04 20:10
PHST- 2022/02/10 00:00 [received]
PHST- 2022/02/25 00:00 [revised]
PHST- 2022/02/28 00:00 [accepted]
PHST- 2022/03/05 06:00 [pubmed]
PHST- 2022/04/23 06:00 [medline]
PHST- 2022/03/04 20:10 [entrez]
AID - S0300-5712(22)00137-3 [pii]
AID - 10.1016/j.jdent.2022.104080 [doi]
PST - ppublish
SO - J Dent. 2022 Apr;119:104080. doi: 10.1016/j.jdent.2022.104080. Epub 2022 Mar 1.

PMID- 34656656
OWN - NLM
STAT- MEDLINE
DCOM- 20220301
LR - 20220301
IS - 1879-176X (Electronic)
IS - 0300-5712 (Linking)
VI - 115
DP - 2021 Dec
TI - Artificial intelligence for caries detection: Randomized trial.
PG - 103849
LID - S0300-5712(21)00272-4 [pii]
LID - 10.1016/j.jdent.2021.103849 [doi]
AB - OBJECTIVES: We aimed to assess the impact of an artificial intelligence
(AI)-based diagnostic-support software for proximal caries detection on bitewing

radiographs. METHODS: A cluster-randomized cross-over controlled trial was
conducted. A commercially available software employing a fully convolutional
neural network for caries detection (dentalXrai Pro, dentalXrai Ltd.) was
randomly employed by 22 dentists, supporting their caries detection on 20
bitewings randomly chosen from a pool of 140 bitewings, with 10 bitewings
randomly being supported by AI and 10 not. The reference test had been
established by 4¬†+¬†1 independent experts in a pixelwise fashion. Caries was
subgrouped as enamel, early dentin and advanced dentin caries, and accuracy and
treatment decisions for each caries lesion assessed. RESULTS: Dentists with AI
showed a significantly higher mean (95% CI) area under the
Receiver-Operating-Characteristics curve (0.89; 0.87-0.90) than those without AI
(0.85; 0.83-0.86; p<0.05), mainly as their sensitivity was significantly higher
(0.81; 0.74-0.87 compared with 0.72; 0.64-0.79; p<0.05) while the specificity was
not significantly affected (p>0.05). This increase in sensitivity was found for
enamel, but not early or advanced dentin lesions. Higher sensitivity came with an
increase in non-invasive, but also invasive treatment decisions (p<0.05).
CONCLUSION: AI can increase dentists' diagnostic accuracy but may also increase
invasive treatment decisions. CLINICAL SIGNIFICANCE: AI can increase dentists'
diagnostic accuracy, mainly via increasing their sensitivity for detecting enamel
lesions, but may also increase invasive therapy decisions. Differences in the
effects of AI for different dentists should be explored, and dentists should be
guided as to which therapy to choose when detecting caries lesions using AI
support.
CI - Copyright ¬© 2021. Published by Elsevier Ltd.
FAU - Mertens, Sarah
AU - Mertens S
AD - Department of Oral Diagnostics, Digital Health and Health Services Research,
Charit√© - Universit√§tsmedizin Berlin, corporate member of Freie Universit√§t
Berlin and Humboldt-Universit√§t zu Berlin, Germany; Department of Operative and

      Preventive Dentistry, Charit√© - Universit√§tsmedizin Berlin,
corporate member of
      Freie Universit√§t Berlin and Humboldt-Universit√§t zu Berlin,
Germany.
FAU - Krois, Joachim
AU - Krois J
AD - Department of Oral Diagnostics, Digital Health and Health Services
Research,
      Charit√© - Universit√§tsmedizin Berlin, corporate member of Freie
Universit√§t
      Berlin and Humboldt-Universit√§t zu Berlin, Germany.
FAU - Cantu, Anselmo Garcia
AU - Cantu AG
AD - Department of Oral Diagnostics, Digital Health and Health Services
Research,
      Charit√© - Universit√§tsmedizin Berlin, corporate member of Freie
Universit√§t
      Berlin and Humboldt-Universit√§t zu Berlin, Germany.
FAU - Arsiwala, Lubaina T
AU - Arsiwala LT
AD - Department of Oral Diagnostics, Digital Health and Health Services
Research,
      Charit√© - Universit√§tsmedizin Berlin, corporate member of Freie
Universit√§t
      Berlin and Humboldt-Universit√§t zu Berlin, Germany.
FAU - Schwendicke, Falk
AU - Schwendicke F
AD - Department of Oral Diagnostics, Digital Health and Health Services
Research,
      Charit√© - Universit√§tsmedizin Berlin, corporate member of Freie
Universit√§t
      Berlin and Humboldt-Universit√§t zu Berlin, Germany. Electronic
address:
      falk.schwendicke@charite.de.
LA - eng
PT - Journal Article
PT - Randomized Controlled Trial
DEP - 20211014
PL - England
TA - J Dent
JT - Journal of dentistry
JID - 0354422
SB - IM
MH - Artificial Intelligence
MH - *Dental Caries/diagnostic imaging/pathology
MH - *Dental Caries Susceptibility
MH - Dental Enamel/diagnostic imaging/pathology
MH - Dentin/diagnostic imaging/pathology
MH - Humans
MH - Neural Networks, Computer
MH - Sensitivity and Specificity
OTO - NOTNLM
OT - Artificial intelligence
OT - Clinical studies/trials

OT - Computer vision
OT - Decision-making
OT - Deep learning
OT - Personalized medicine
EDAT- 2021/10/18 06:00
MHDA- 2022/03/03 06:00
CRDT- 2021/10/17 20:38
PHST- 2021/08/06 00:00 [received]
PHST- 2021/10/05 00:00 [revised]
PHST- 2021/10/08 00:00 [accepted]
PHST- 2021/10/18 06:00 [pubmed]
PHST- 2022/03/03 06:00 [medline]
PHST- 2021/10/17 20:38 [entrez]
AID - S0300-5712(21)00272-4 [pii]
AID - 10.1016/j.jdent.2021.103849 [doi]
PST - ppublish
SO - J Dent. 2021 Dec;115:103849. doi: 10.1016/j.jdent.2021.103849. Epub 2021 Oct 14.

PMID- 34413414
OWN - NLM
STAT- MEDLINE
DCOM- 20211112
LR - 20211112
IS - 2045-2322 (Electronic)
IS - 2045-2322 (Linking)
VI - 11
IP - 1
DP - 2021 Aug 19
TI - Deep learning for early dental caries detection in bitewing radiographs.
PG - 16807
LID - 10.1038/s41598-021-96368-7 [doi]
LID - 16807
AB - The early detection of initial dental caries enables preventive treatment, and
      bitewing radiography is a good diagnostic tool for posterior initial caries. In
      medical imaging, the utilization of deep learning with convolutional neural
      networks (CNNs) to process various types of images has been actively researched,
      with promising performance. In this study, we developed a CNN model using a
      U-shaped deep CNN (U-Net) for caries detection on bitewing radiographs and
      investigated whether this model can improve clinicians' performance. The research
      complied with relevant ethical regulations. In total, 304 bitewing radiographs
      were used to train the CNN model and 50 radiographs for performance evaluation.
      The diagnostic performance of the CNN model on the total test dataset was as

follows: precision, 63.29%; recall, 65.02%; and F1-score, 64.14%, showing quite
accurate performance. When three dentists detected caries using the results of
the CNN model as reference data, the overall diagnostic performance of all three
clinicians significantly improved, as shown by an increased sensitivity ratio
(D1, 85.34%; D1', 92.15%; D2, 85.86%; D2', 93.72%; D3, 69.11%; D3', 79.06%;
p,Äâ<,Äâ0.05). These increases were especially significant (p,Äâ<,Äâ0.05) in the initial
and moderate caries subgroups. The deep learning model may help clinicians to
diagnose dental caries more accurately.
CI - ¬© 2021. The Author(s).
FAU - Lee, Shinae
AU - Lee S
AD - Department of Conservative Dentistry, Gangnam Severance Hospital, College of
Dentistry, Yonsei University, 146-92 Dogok-dong, Gangnam-gu, Seoul, 135-720,
Korea.
FAU - Oh, Sang-Il
AU - Oh SI
AD - SELVAS AI Inc., Seoul, 08594, Korea.
FAU - Jo, Junik
AU - Jo J
AD - SELVAS AI Inc., Seoul, 08594, Korea.
FAU - Kang, Sumi
AU - Kang S
AD - Department of Conservative Dentistry, College of Dentistry, Yonsei University,
Seoul, Korea.
FAU - Shin, Yooseok
AU - Shin Y
AD - Department of Conservative Dentistry, College of Dentistry, Yonsei University,
Seoul, Korea.
FAU - Park, Jeong-Won
AU - Park JW
AD - Department of Conservative Dentistry, Gangnam Severance Hospital, College of
Dentistry, Yonsei University, 146-92 Dogok-dong, Gangnam-gu, Seoul, 135-720,
Korea. pjw@yuhs.ac.
LA - eng
PT - Journal Article
DEP - 20210819
PL - England
TA - Sci Rep
JT - Scientific reports
JID - 101563288
SB - IM

MH - *Deep Learning
MH - Dental Caries/*diagnosis/*diagnostic imaging
MH - Humans
MH - Neural Networks, Computer
MH - *Radiography, Bitewing
PMC - PMC8376948
COIS- The authors declare no competing interests.
EDAT- 2021/08/21 06:00
MHDA- 2021/11/16 06:00
PMCR- 2021/08/19
CRDT- 2021/08/20 06:37
PHST- 2021/04/05 00:00 [received]
PHST- 2021/08/06 00:00 [accepted]
PHST- 2021/08/20 06:37 [entrez]
PHST- 2021/08/21 06:00 [pubmed]
PHST- 2021/11/16 06:00 [medline]
PHST- 2021/08/19 00:00 [pmc-release]
AID - 10.1038/s41598-021-96368-7 [pii]
AID - 96368 [pii]
AID - 10.1038/s41598-021-96368-7 [doi]
PST - epublish
SO - Sci Rep. 2021 Aug 19;11(1):16807. doi: 10.1038/s41598-021-96368-7.

PMID- 33384840
OWN - NLM
STAT- PubMed-not-MEDLINE
LR - 20220419
IS - 2213-8862 (Electronic)
IS - 1991-7902 (Print)
IS - 1991-7902 (Linking)
VI - 16
IP - 1
DP - 2021 Jan
TI - Developments, application, and performance of artificial intelligence in
      dentistry - A systematic review.
PG - 508-522
LID - 10.1016/j.jds.2020.06.019 [doi]
AB - BACKGROUND/PURPOSE: Artificial intelligence (AI) has made deep inroads into
      dentistry in the last few years. The aim of this systematic review was to
      identify the development of AI applications that are widely employed in dentistry
      and evaluate their performance in terms of diagnosis, clinical decision-making,
      and predicting the prognosis of the treatment. MATERIALS AND METHODS: The
      literature for this paper was identified and selected by performing a thorough
      search in the electronic data bases like PubMed, Medline, Embase, Cochrane,
      Google scholar, Scopus, Web of science, and Saudi digital library published over

the past two decades (January 2000-March 15, 2020).After applying inclusion and
exclusion criteria, 43 articles were read in full and critically analyzed.
Quality analysis was performed using QUADAS-2. RESULTS: AI technologies are
widely implemented in a wide range of dentistry specialties. Most of the
documented work is focused on AI models that rely on convolutional neural
networks (CNNs) and artificial neural networks (ANNs). These AI models have been
used in detection and diagnosis of dental caries, vertical root fractures, apical
lesions, salivary gland diseases, maxillary sinusitis, maxillofacial cysts,
cervical lymph nodes metastasis, osteoporosis, cancerous lesions, alveolar bone
loss, predicting orthodontic extractions, need for orthodontic treatments,
cephalometric analysis, age and gender determination. CONCLUSION: These studies
indicate that the performance of an AI based automated system is excellent. They
mimic the precision and accuracy of trained specialists, in some studies it was
found that these systems were even able to outmatch dental specialists in terms
of performance and accuracy.
CI - ¬© 2020 Association for Dental Sciences of the Republic of China. Publishing
services by Elsevier B.V.
FAU - Khanagar, Sanjeev B
AU - Khanagar SB
AD - Preventive Dental Science Department, College of Dentistry, King Saud Bin
Abdulaziz University for Health Sciences, Riyadh, Saudi Arabia.
AD - King Abdullah International Medical Research Center, Riyadh, Saudi Arabia.
FAU - Al-Ehaideb, Ali
AU - Al-Ehaideb A
AD - Preventive Dental Science Department, College of Dentistry, King Saud Bin
Abdulaziz University for Health Sciences, Riyadh, Saudi Arabia.
AD - King Abdullah International Medical Research Center, Riyadh, Saudi Arabia.
AD - Dental Services, King Abdulaziz Medical City- Ministry of National Guard Health
Affairs, Riyadh, Saudi Arabia.
FAU - Maganur, Prabhadevi C
AU - Maganur PC
AD - Department of Preventive Dental Sciences, Division of Pedodontics, College of
Dentistry, Jazan University, Jazan, Saudi Arabia.

FAU - Vishwanathaiah, Satish
AU - Vishwanathaiah S
AD - Department of Preventive Dental Sciences, Division of Pedodontics, College of
      Dentistry, Jazan University, Jazan, Saudi Arabia.
FAU - Patil, Shankargouda
AU - Patil S
AD - Department of Maxillofacial Surgery and Diagnostic Sciences, Division of Oral
      Pathology, College of Dentistry, Jazan University, Jazan, Saudi Arabia.
FAU - Baeshen, Hosam A
AU - Baeshen HA
AD - Consultant in Orthodontics, Department of Orthodontics, College of Dentistry,
      King Abdulaziz University, Jeddah, Saudi Arabia.
FAU - Sarode, Sachin C
AU - Sarode SC
AD - Department of Oral and Maxillofacial Pathology, Dr. D.Y.Patil Dental College and
      Hospital, Dr. D. Y. Patil Vidyapeeth, Pimpri, Pune 411018, Maharashtra, India.
FAU - Bhandi, Shilpa
AU - Bhandi S
AD - Department of Restorative Dental Sciences, Division of Operative Dentistry,
      College of Dentistry, Jazan University, Saudi Arabia.
LA - eng
PT - Journal Article
PT - Review
DEP - 20200630
PL - Netherlands
TA - J Dent Sci
JT - Journal of dental sciences
JID - 101293181
PMC - PMC7770297
OTO - NOTNLM
OT - Artificial intelligence dentistry
OT - Artificial neural networks
OT - Computer-aided diagnosis
OT - Convolutional neural networks
OT - Deep learning models
OT - Machine learning
EDAT- 2021/01/02 06:00
MHDA- 2021/01/02 06:01
PMCR- 2020/06/30
CRDT- 2021/01/01 05:22
PHST- 2020/06/02 00:00 [received]
PHST- 2020/06/19 00:00 [revised]
PHST- 2021/01/01 05:22 [entrez]
PHST- 2021/01/02 06:00 [pubmed]
PHST- 2021/01/02 06:01 [medline]
PHST- 2020/06/30 00:00 [pmc-release]
AID - S1991-7902(20)30143-4 [pii]

AID - 10.1016/j.jds.2020.06.019 [doi]
PST - ppublish
SO - J Dent Sci. 2021 Jan;16(1):508-522. doi: 10.1016/j.jds.2020.06.019. Epub 2020 Jun
      30.

PMID- 33198554
OWN - NLM
STAT- MEDLINE
DCOM- 20210423
LR - 20210423
IS - 1544-0591 (Electronic)
IS - 0022-0345 (Print)
IS - 0022-0345 (Linking)
VI - 100
IP - 4
DP - 2021 Apr
TI - Cost-effectiveness of Artificial Intelligence for Proximal Caries Detection.
PG - 369-376
LID - 10.1177/0022034520972335 [doi]
AB - Artificial intelligence (AI) can assist dentists in image assessment, for
      example, caries detection. The wider health and cost impact of employing AI for
      dental diagnostics has not yet been evaluated. We compared the cost-effectiveness
      of proximal caries detection on bitewing radiographs with versus without AI.
      U-Net, a fully convolutional neural network, had been trained, validated, and
      tested on 3,293, 252, and 141 bitewing radiographs, respectively, on which 4
      experienced dentists had marked carious lesions (reference test). Lesions were
      stratified for initial lesions (E1/E2/D1, presumed noncavitated, receiving caries
      infiltration if detected) and advanced lesions (D2/D3, presumed cavitated,
      receiving restorative care if detected). A Markov model was used to simulate the
      consequences of true- and false-positive and true- and false-negative detections,
      as well as the subsequent decisions over the lifetime of patients. A German
      mixed-payers perspective was adopted. Our health outcome was tooth retention
      years. Costs were measured in 2020 euro. Monte-Carlo microsimulations and
      univariate and probabilistic sensitivity analyses were conducted. The incremental
      cost-effectiveness ratio (ICER) and the cost-effectiveness acceptability at

different willingness-to-pay thresholds were quantified. AI showed an accuracy of
0.80; dentists' mean accuracy was significantly lower at 0.71 (minimum-maximum:
0.61-0.78, P,Äâ<,Äâ0.05). AI was significantly more sensitive than dentists (0.75 vs.
0.36 [0.19-0.65]; P,Äâ=,Äâ0.006), while its specificity was not significantly lower
(0.83 vs. 0.91 [0.69-0.98]; P,Äâ>,Äâ0.05). In the base-case scenario, AI was more
effective (tooth retention for a mean 64 [2.5%-97.5%: 61-65] y) and less costly
(298 [244-367] euro) than assessment without AI (62 [59-64] y; 322 [257-394]
euro). The ICER was -13.9 euro/y (i.e., AI saved money at higher effectiveness).
In the majority (>77%) of all cases, AI was less costly and more effective.
Applying AI for caries detection is likely to be cost-effective, mainly as fewer
lesions remain undetected. Notably, this cost-effectiveness requires dentists to
manage detected early lesions nonrestoratively.
FAU - Schwendicke, F
AU - Schwendicke F
AUID- ORCID: 0000-0003-1223-1669
AD - Department of Oral Diagnostics, Digital Health and Health Services Research,
Charit√©-Universit√§tsmedizin Berlin, Berlin, Germany.
FAU - Rossi, J G
AU - Rossi JG
AD - Department of Oral Diagnostics, Digital Health and Health Services Research,
Charit√©-Universit√§tsmedizin Berlin, Berlin, Germany.
FAU - G√∂stemeyer, G
AU - G√∂stemeyer G
AD - Department of Operative and Preventive Dentistry, Charit√©-Universit√§tsmedizin
Berlin, Berlin, Germany.
FAU - Elhennawy, K
AU - Elhennawy K
AD - Department of Orthodontics, Dentofacial Orthopedics and Pedodontics,
Charit√©-Universit√§tsmedizin Berlin, Berlin, Germany.
FAU - Cantu, A G
AU - Cantu AG
AD - Department of Oral Diagnostics, Digital Health and Health Services Research,
Charit√©-Universit√§tsmedizin Berlin, Berlin, Germany.
FAU - Gaudin, R
AU - Gaudin R
AD - Department of Oral and Maxillofacial Surgery, Charit√©-Universit√§tsmedizin Berlin,
Berlin, Germany.

FAU - Chaurasia, A
AU - Chaurasia A
AD - Department of Oral Medicine and Radiology, King George's Medical University,
      Lucknow, India.
FAU - Gehrung, S
AU - Gehrung S
AD - Department of Oral Diagnostics, Digital Health and Health Services Research,
      Charit√©-Universit√§tsmedizin Berlin, Berlin, Germany.
FAU - Krois, J
AU - Krois J
AD - Department of Oral Diagnostics, Digital Health and Health Services Research,
      Charit√©-Universit√§tsmedizin Berlin, Berlin, Germany.
LA - eng
PT - Journal Article
DEP - 20201116
PL - United States
TA - J Dent Res
JT - Journal of dental research
JID - 0354343
SB - IM
MH - Artificial Intelligence
MH - Cost-Benefit Analysis
MH - *Dental Caries/diagnosis
MH - *Dental Caries Susceptibility
MH - Humans
MH - Monte Carlo Method
PMC - PMC7985854
OTO - NOTNLM
OT - caries diagnosis/prevention
OT - computer simulation
OT - decision making
OT - dental
OT - economic evaluation
OT - radiology
COIS- Declaration of Conflicting Interests: The authors declared the following
      potential conflicts of interest with respect to the research, authorship, and/or
      publication of this article: F. Schwendicke, R. Gaudin, and J. Krois are
      cofounders of a Charit√© startup on dental image analysis. The conduct, analysis,
      and interpretation of this study and its findings were unrelated to this.
EDAT- 2020/11/18 06:00
MHDA- 2021/04/24 06:00
PMCR- 2021/03/23
CRDT- 2020/11/17 05:41
PHST- 2020/11/18 06:00 [pubmed]
PHST- 2021/04/24 06:00 [medline]
PHST- 2020/11/17 05:41 [entrez]

PHST- 2021/03/23 00:00 [pmc-release]
AID - 10.1177_0022034520972335 [pii]
AID - 10.1177/0022034520972335 [doi]
PST - ppublish
SO - J Dent Res. 2021 Apr;100(4):369-376. doi: 10.1177/0022034520972335.
Epub 2020 Nov
      16.
